# A complex stereochemical relay approach to the antimalarial alkaloid ocimicide A_1_. Evidence for a structural revision†
†Electronic supplementary information (ESI) available: Supplementary schemes, figures, and tables, general experimental remarks, synthetic procedures, catalogues of experimental and calculated nuclear magnetic resonance spectra. CCDC 1536046. For ESI and crystallographic data in CIF or other electronic format see DOI: 10.1039/c7sc01127j



**DOI:** 10.1039/c7sc01127j

**Published:** 2017-05-04

**Authors:** Herman Nikolayevskiy, Maung Kyaw Moe Tun, Paul R. Rablen, Choukri Ben Mamoun, Seth B. Herzon

**Affiliations:** a Department of Chemistry , Yale University , New Haven , CT 06520 , USA . Email: seth.herzon@yale.edu; b Department of Chemistry and Biochemistry , Swarthmore College , Swarthmore , PA 19081 , USA; c Department of Internal Medicine , Yale School of Medicine , New Haven , CT 06520 , USA; d Department of Pharmacology , Yale School of Medicine , New Haven , CT 06520 , USA

## Abstract

Ocimicide A_1_ (**1**) and the semisynthetic derivative ocimicide A_2_ (**2**) are highly potent antimalarial agents efficacious against chloroquine-sensitive and -resistant *Plasmodium falciparum* strains with IC_50_ values in the nanomolar and picomolar range, respectively. Members of this family have demonstrated radical cure in rhesus monkeys, without detectable toxicity, but their structure–function relationships and mechanism of action are unknown. Herein we describe a twelve-step synthesis of an advanced *N*-acylated pentacyclic precursor to the proposed structure of **1** (11% overall yield). Instability and poor *P. falciparum* growth inhibition of the corresponding free donor–acceptor cyclopropylamine, and large discrepancies between reported and both experimental and DFT-calculated ^13^C chemical shifts and coupling constants, suggest that substantial revision of the proposed structures may be necessary.

## Abstract 2

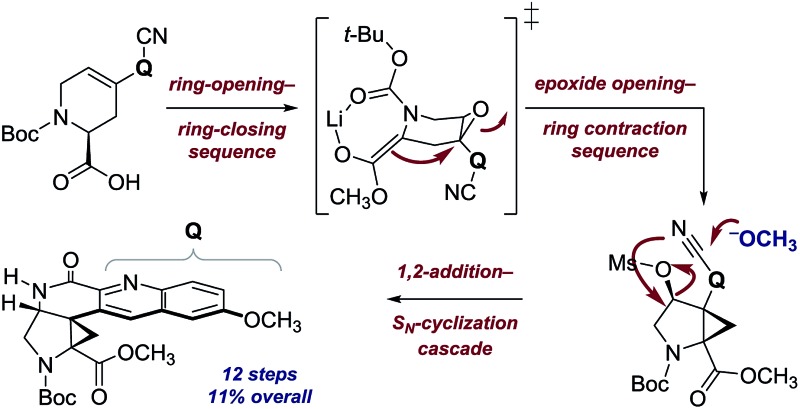
The core structure of the potent antimalarial alkaloid ocimicide A_1_ was prepared by a complex stereochemical relay. Computational studies suggest a structural revision of the metabolite is necessary.

## 

The structures **1** and **3**, designated ocimicide A_1_ and B_1_, respectively (Fig. 1), were disclosed in the patent literature^1^ as hexacyclic quinoline alkaloids found in the root bark of *Ocimum sanctum*. The semisynthetic derivatives ocimicide A_2_ (**2**) and ocimicide B_2_ (**4**) were prepared in one step from **1** and **3**, respectively. The structures of **1–4** were determined by NMR, UV, IR, and HRMS analyses. Tabulated NMR spectroscopic data for **2** and **4** were provided, but no spectroscopic data for the natural isolates **1** and **3** were disclosed.^1^ All four compounds contain a tetrasubstituted aminocyclopropane and oxidized pyrrolidine rings, but the orientation of the quinoline rings in **1**/**2** and **3**/**4** are, surprisingly, different. The alkaloids **1–4** demonstrated potent activity against chloroquine (**5**)-sensitive and -resistant *P. falciparum* strains (IC_50_s = 26–35 nM, 0.7–1.1 nM for natural and semisynthetic derivatives, respectively).^1^ The compounds were highly selective in cell culture, and efficacious in both prophylactic and treatment assays in the *P. berghei* malaria murine model. In a separate patent, structurally-related isolates were reported to effect radical cure in rhesus monkeys, without detectable toxicity.^2^

**Fig. 1:**
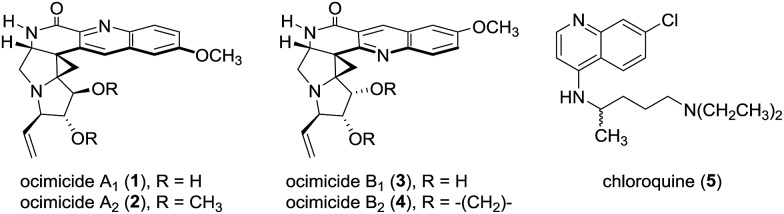
Structures of the alkaloids ocimicides A_1_ (**1**) and B_1_ (**3**), the semisynthetic derivatives ocimicides A_2_ (**2**) and B_2_ (**4**), and chloroquine (**5**).

With resistance to the front-line antimalarial artemisinin increasing,^3^ there is a pressing need for the development of novel agents with unique modes of action.^4^ We initiated synthetic studies toward ocimicide A_1_ (**1**), with the goal of elucidating the structure–function relationships and mechanism of action of this new class of antimalarials.

Since E-ring substitution influences activity,^1^ we targeted late-stage construction of this ring from the pentacyclic intermediate **6** (Scheme 1). A tandem epoxide-opening–ring contraction of **8**, followed by activation and invertive displacement of the alcohol **7**, was used to install the aminocyclopropane^5^ and lactam substituents of **6**. The epoxide **8** was simplified to the vinyl triflate **9** and the stannane **10**. The vinyl triflate **9** was prepared in racemic form in four steps and 57% overall yield from 4-methoxypyridine by a sequence developed by Comins^6^ for closely-related substrates (see ESI†). The stannane **10** was prepared in one step and 70% yield by site-selective metalation^7^ of 2-cyano-6-methoxyquinoline^8^ with lithium tetramethylpiperidide, followed by the addition of trimethyltin chloride (see ESI†).

**Scheme 1:**
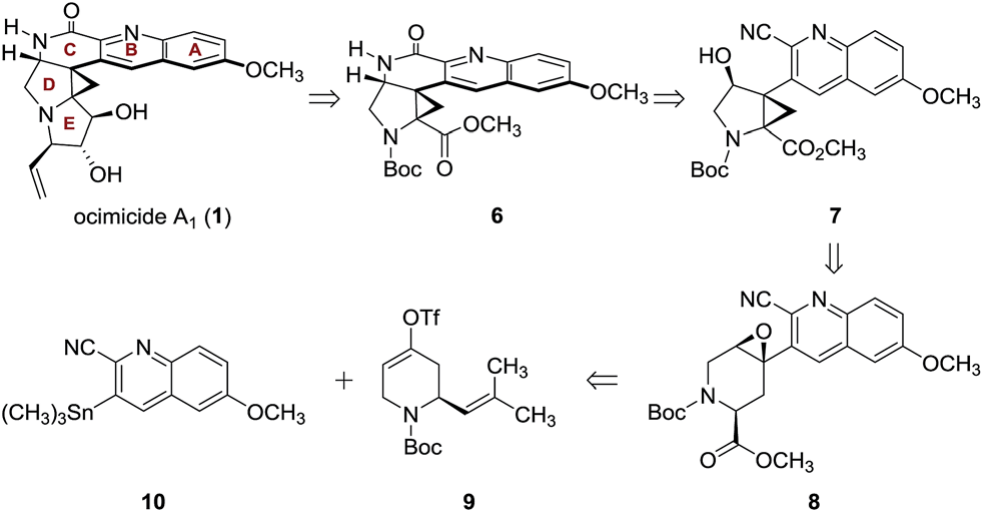
Retrosynthetic analysis of ocimicide A_1_ (**1**).

Stille coupling of the vinyl triflate **9** and the stannane **10** [tetrakis(triphenylphosphine)palladium, copper iodide, cesium fluoride]^9^ provided the coupling product **11** in >99% yield (Scheme 2A). Site-selective oxidative cleavage of the exocyclic alkene within **11** [osmium tetroxide, then bis(acetoxy)iodobenzene],^10^ followed by *in situ* oxidation under Pinnick–Lindgren conditions^11^ provided the acid **12**. The carboxylic acid function was employed in a carefully choreographed multistep relay to control the relative stereochemistry of the target. Treatment of **12** with *N*-bromosuccinimide and 4-dimethylaminopyridine provided the bromolactone **13** as a single regioisomer. The bromolactone **13** could be isolated, but in practice was treated directly with potassium carbonate in methanol to induce a ring-opening–ring-closing sequence, to form the epoxy ester **8** (45% from **11**). The remaining mass balance was attributed to formation of a cyclic imidate resulting from addition of the alkoxide intermediate to the nitrile.

**Scheme 2:**
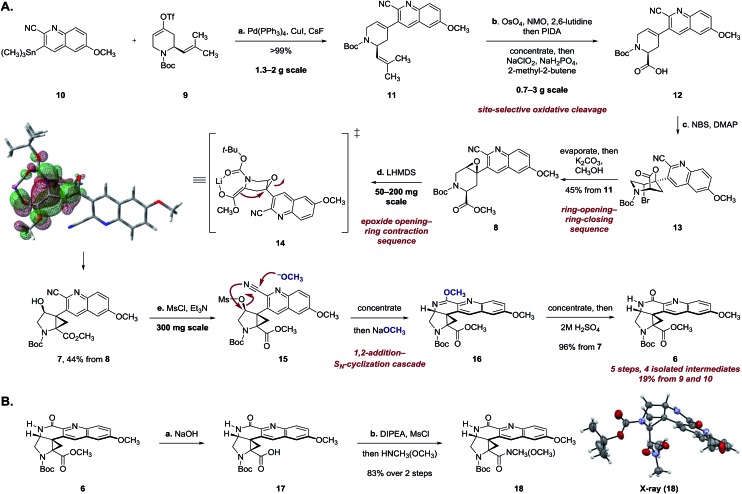
(A) Synthesis of the lactam **6**. Reaction conditions: (a) Pd(PPh_3_)_4_, CsF, CuI, DMF, 24 °C, >99%; (b) 2,6-lutidine, *N*-methylmorpholine *N*-oxide, OsO_4_, CH_3_COCH_3_–H_2_O (9 : 1), 24 °C, then bis(acetoxyiodo)benzene, 24 °C, evaporate, then NaClO_2_, NaH_2_PO_4_, 2-methyl-2-butene, THF–H_2_O–*t*-BuOH (4 : 3 : 1), 24 °C; (c) 4-dimethylaminopyridine, *N*-bromosuccinimide, CH_2_Cl_2_, 24 °C, evaporate, then K_2_CO_3_, CH_3_OH, 24 °C, 45% from **11**; (d) lithium bis(trimethylsilyl)amide, PhCH_3_, 103 °C, 44%; (e) methanesulfonyl chloride, triethylamine, CH_2_Cl_2_, 0 → 24 °C, then NaOCH_3_, CH_3_OH, 24 °C; evaporate, then NaOCH_3_, CH_3_OH, 65 °C; evaporate, then 2 M H_2_SO_4_, THF, 24 °C, 96% from **7**. The HOMO of the enolate **14** was calculated using M062X/6-31+G(2df,p) with simulated *tert*-butanol as solvent and a lithium counterion. (B) Synthesis of the Weinreb amide **18**. Reaction conditions: (a) NaOH, CH_3_OH, 80 °C; (b) methanesulfonyl chloride, *N*,*N*-di-*iso*-propylethylamine, THF, 0 °C, then *N*,*O*-dimethylhydroxylamine, 83% over 2 steps.

Extensive experimentation was required to develop conditions to effect the epoxide-opening–ring contraction sequence (Scheme 2A).^12^ Ultimately, we found that treatment of **8** with lithium hexamethyldisilazide (1.10 equiv.) in toluene at 103 °C provided the cyclopropyl alcohol **7** in 44% yield (+10% unreacted **8**). The temperature profile of this step was critical; although deprotonation occurred at 24 °C (as evidenced by deuterium incorporation experiments), heating to 103 °C for short amounts of time (2 h) was essential to obtain the product **7** in workable yields. Higher temperatures and longer reaction times led to degradation.

Density functional theory calculations [M062X/6-31+G(2df,p)] suggest that the enolate is required to adopt a boat-like conformation to effect invertive opening of the epoxide (see **14**). This conformation orients the quinoline ring in a pseudoaxial position, leading to destabilizing non-bonded interactions in the transition state. The calculated activation energy of 27.9 kcal mol^–1^ for this step is in agreement with the experimentally-determined reaction temperature (103 °C).

The synthesis of **6** was completed by a high-yielding cascade sequence. Exposure of the cyclopropyl alcohol **7** to methanesulfonyl chloride and triethylamine provided the mesylate **15**. Following concentration of the reaction mixture, the unpurified mesylate **15** was dissolved in anhydrous methanol and treated with sodium methoxide to effect 1,2-addition of methoxide to the nitrile and invertive displacement of the mesylate (**15** → **16**). Concentration of the reaction mixture, followed by dilution with sulfuric acid, resulted in smooth hydrolysis of the methyl imidate **16** to provide the key pentacycle **6** (96% from **7**). The facile addition of methoxide to **15** is likely a reflection of electrophilic activation of the nitrile substituent by the quinoline ring. By this route, the racemic lactam **6** was obtained in only five steps, four isolated intermediates, and 19% yield from the Stille coupling partners **9** and **10** (twelve steps and 11% yield overall from commercial reagents).

The structural assignment of **6** was confirmed by X-ray analysis of the crystalline Weinreb amide **18**,^13^ which was prepared by saponification, activation of the resulting acid **17** with methanesulfonyl chloride,^14^ and addition of *N*-methoxy-*N*-methylamine (Scheme 2B; 83% overall). While **6**, **18**, and related *N*-acylated intermediates were amenable to purification and handling, the corresponding free amines were unstable and underwent rapid decomposition. For example, attempted neutralization of the trifluoroacetate salt **19**, formed by exposure of **6** to trifluoroacetic acid, led to extensive decomposition (Scheme 3). Although the complexity of the decomposition mixtures precluded characterization, we believe that the conjugation of the secondary lactam through the electron-deficient quinoline ring effectively renders the cyclopropane within **19** a donor–acceptor system.^15^ Decomposition may occur by amine-initiated ring-opening.

**Scheme 3:**
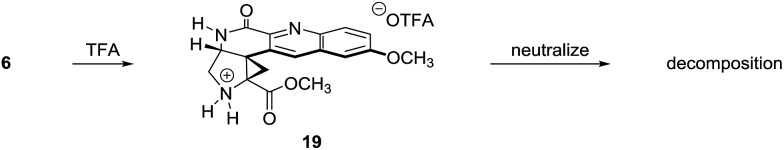
Synthesis of the trifluoroacetate salt **19**.

The instability of our synthetic intermediates prompted us to reexamine the original structural assignment using density functional theory.^16^ As spectroscopic data for **1** were not disclosed,^1^ we focused on the dimethyl ether derivative ocimicide A_2_ (**2**), for which tabulated spectroscopic shifts were presented. Following the protocol of Hoye and co-workers,^17^ 32 structures (corresponding to all possible diastereomers at nitrogen 15 and carbons 12, 13, 14 and 17 of **2**,^18^ Fig. 2) were generated. These structures were imported into BOSS^19^ and each was separately subjected to a conformational search. Conformers within 5.02 kcal mol^–1^ of the lowest energy isomer (8–30 conformers for each diastereomer) were advanced to density functional theory geometry optimization [gas phase, B3LYP/6-31+G(d,p)]. Geometry-optimized conformers were confirmed as real local-minima by the absence of imaginary frequencies. The chemical shifts of the optimized conformers were then calculated using a modified WC04 functional^20^ and 6-31G(d) basis set in methanol (an initial screen of several functional and basis set combinations indicated that this level of theory provided an acceptable compromise of computational time and accuracy). Finally, the ^13^C chemical shifts were Boltzmann-averaged to generate the heat maps shown in Tables S1 and S2.† The geometry optimization and NMR calculation methods selected were benchmarked against 19-(*E*)-hunteracine (**20**),^21^ the structure of which has been unequivocally established by X-ray analysis.^22^ The mean absolute error (MAE) for **20** was 2.8 ppm [absolute error (AE) = 0.2–5.7 ppm], which was within the error typically obtained from similar levels of theory under optimized conditions.^23^

**Fig. 2:**
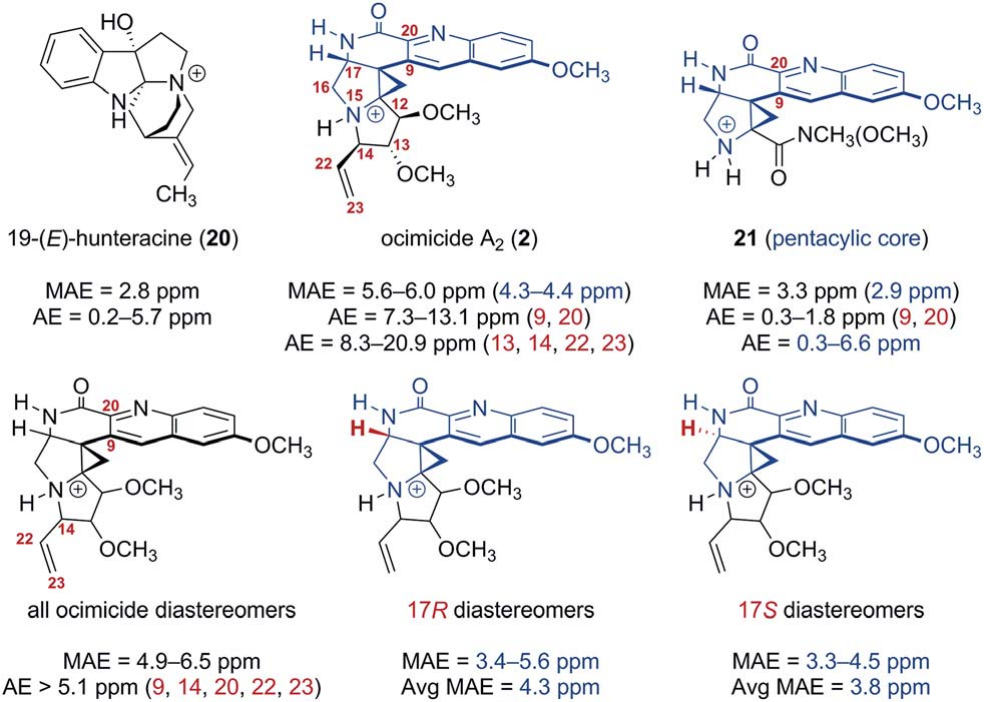
Mean absolute error (MAE) and absolute error (AE) of calculated ^13^C chemical shifts for the reference compound 19-(*E*)-hunteracine (**20**), ocimicide A_2_ (**2**; numbered as in patent^1^), the trifluoroacetate salt **21**, and all ocimicide diastereomers (varied configuration at carbons 12, 13, 14, 17, and nitrogen 15). Geometries were optimized using B3LYP/6-31+G(d,p). ^13^C NMR chemical shifts were calculated using modified WC04/6-31G(d). Data highlighted in blue corresponds to the pentacyclic core.

Large deviations, particularly in proximity to the lactam (carbons 9 and 20; AE = 7.3–13.1 ppm) and pyrrolidine rings (carbons 13, 14, 22, and 23; AE = 8.3–20.9 ppm), were observed between the calculated and reported^1^ ^13^C chemical shifts for ocimicide A_2_ (**2**) (MAE = 5.6–6.0 ppm). While 17 of the 30 diastereomers investigated achieved better agreement (MAE = 4.9–5.5 ppm), no diastereomer could, within acceptable error, replicate reported values at carbons 14, 22, or 23 (AE > 6.6 ppm).

For comparison, the trifluoroacetate salt **21** was synthesized (*via* treatment of **18** with trifluoroacetic acid) and computationally subjected to analogous geometry optimization and NMR calculation methods.^24^ A focused analysis of pentacyclic core MAE values for **2**, **21**, and alternate ocimicide diastereomers revealed that while **21** was comparable in error to the benchmark **20** (2.9 ppm *vs.* 2.8 ppm), ocimicide diastereomers (including **2**) had, on average, higher MAEs (4.3 ppm for 17*R*; 3.8 ppm for 17*S*). Moreover, calculated AE values for carbons 9 and 20 were significantly lower for **21** than any ocimicide diastereomer (0.3–1.8 ppm *vs.* 5.1–13.8 ppm). These data suggest our computational methods achieved an acceptable level of accuracy in this system.

The rigidity of the azabicyclo[3.1.0] ring system present within **6** and **2** suggests that a reasonable comparison of coupling constants may be made between the methine proton of carbon 17 and the methylene protons of carbon 16. For this three-proton spin system, the *J* values observed in **6** (5.2 and 0 Hz) poorly match those reported for **2** (9.2 and 4.4 Hz). For comparison, coupling constants were calculated for all diastereomers with the reported 17*R* and the alternate 17*S* configuration. Interestingly, while *J* values for the reported diastereomers (3.3–4.1 Hz and 0.1–0.3 Hz) closely resemble the experimental *J* values of **6**, the reported data for **2** is better approximated by the alternate diastereomer (7.6–8.1 Hz and 5.7–6.7 Hz). While not definitive, the computational studies described herein suggest that a structural revision of the ocimicides is required.

Finally, to probe the antimalarial activity of our synthetic intermediates, we examined the growth inhibitory potential of several compounds against the *P. falciparum* 3D7 isolate (Table 1). While the bromolactone **13**, the epoxy ester **8**, and the cyclopropyl alcohol **7** inhibited parasitic growth by 20–28% at 100 nM, more advanced synthetic intermediates were significantly less active. The pentacyclic lactam **6** completely failed to inhibit parasitic growth, while the amine **19** (Scheme 3) demonstrated only 15% inhibition at 500 nM. These data and in particular the low activity of **6** and **19** provide some circumstantial support for a structural revision of the metabolites.

**Table 1:** Percent inhibition of wild-type *P. falciparum* (3D7) growth by several synthetic intermediates at 100 nM and 500 nM

Compound	% inhibition, 100 nM	% inhibition, 500 nM
**13**	26	40
**8**	20	30
**7**	28	41
**6**	0	0
**19**	8	15

## Conclusions

In summary, we have described an expedient synthesis of the lactam **6**, an advanced pentacyclic intermediate that contains the tetrasubstituted aminocyclopropane of the ocimicide alkaloids. Our synthesis of **6** proceeds in five steps, four isolated intermediates, and 19% yield from the vinyl triflate **9** and the stannane **10** and twelve steps and 11% yield overall from commercial reagents. Notable features of the synthesis include a complex multistep stereochemical relay to establish the relative configuration of the target and the formation of a tetrasubstituted cyclopropylamine *via* an epoxide-opening–ring contraction sequence. Our NMR calculations suggest that the published structures of the ocimicides may require revision. Given the reported success of these alkaloids to achieve radical cure of malaria in rhesus monkeys,^2^ efforts to fully elucidate their structures are ongoing.^25^ These studies highlight the ability of modern computational methods to guide experimental synthesis.
