# Supplementary material for: A complex stereochemical relay approach to the antimalarial alkaloid ocimicide A1. Evidence for a structural revision
Source: Chem Sci. 2017 May 4;8(7):4867–71. doi: 10.1039/c7sc01127j (PMC5603897; doi:10.1039/c7sc01127j)
Supplement: Supplementary file 1 [file SC-008-C7SC01127J-s001.pdf]

Electronic Supplementary Material (ESI) for Chemical Science.
This journal is © The Royal Society of Chemistry 2017

A complex stereochemical relay approach to the antimalarial alkaloid ocimicide A_1. Evidence for a structural revision.

Herman Nikolayevskiy,[a] Maung Kyaw Moe Tun,[a] Paul R. Rablen,[b] Choukri Ben Mamoun,[c] and Seth B. Herzon*[a],[d]

[a]Department of Chemistry, Yale University, 225 Prospect Street, New Haven, CT 06520 (USA)

[b]Department of Chemistry and Biochemistry, Swarthmore College, Swarthmore, PA 19081 (USA)

[c]Department of Internal Medicine, Yale School of Medicine, New Haven, CT 06520 (USA)

[d]Department of Pharmacology, Yale School of Medicine, New Haven, CT 06520 (USA)

Submitted to Chem. Sci.

Supporting Information

Index

Supplementary Schemes **S2**
Supplementary Figures and Tables **S5**
General Experimental Methods **S14**
Materials **S15**
Instrumentation **S15**
Synthetic Procedures **S17**
Catalog of Nuclear Magnetic Resonance Spectra **S32**
Crystallographic data for the Weinreb amide 18 .... **S69**
Bibliography **S103**
Catalog of Calculated Coordinates, Energies, and ^1H and ^{13}C Chemical Shifts **SA1**

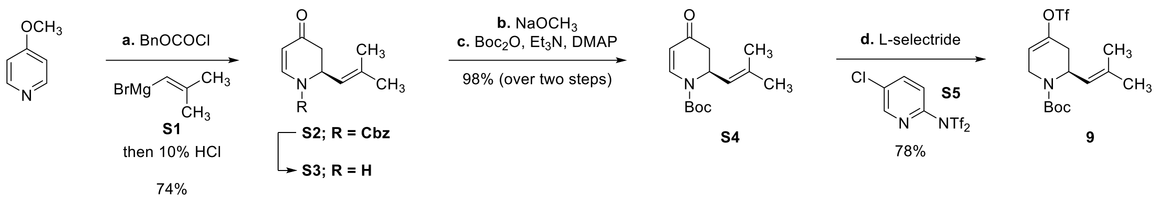

Scheme S1. Synthesis of the vinyl triflate **9**. Reaction conditions: (a) 2-methyl-1-propenylmagnesium bromide (**S1**), benzyl chloroformate, THF, –23 °C, then 10% HCl, –23→24 °C, 74%; (b) $NaOCH_3$, CH_3OH, 65 °C, >99%; (c) di-*tert*-butyl dicarbonate, 4-dimethylaminopyridine, triethylamine, THF, 0°C, 98%; (d) lithium tri-*sec*-butylborohydride, Comins' reagent (**S5**), THF, –78→24 °C, 78%.

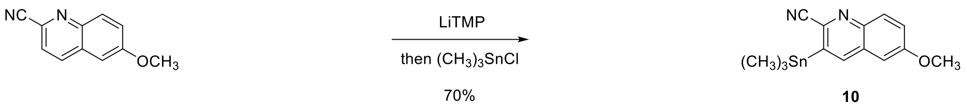

Scheme S2. Synthesis of the stannane **10**. Reaction conditions: lithium 2,2,6,6-tetramethylpiperidide, THF, –78 °C, then trimethyltin chloride, 70%.

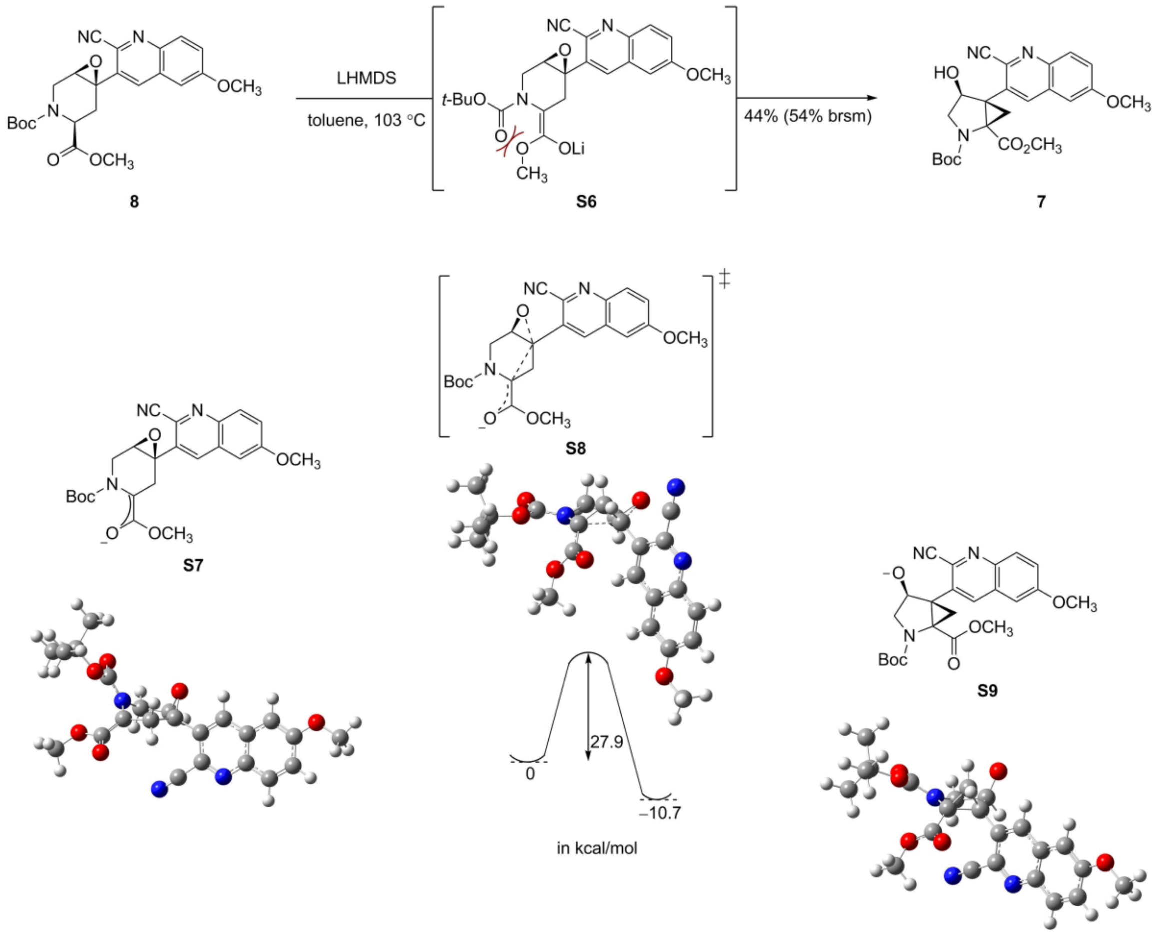

Scheme S3. Base-mediated ring opening–ring contraction of **8** to provide the cyclopropyl alcohol **7**. The reaction pathway and relative energies of the intermediates **S7** and **S9** and the transition state **S8** were calculated using M062X/6-31+G(2df,p) with simulated *tert*-butanol as solvent and no lithium counterion. The high energy barrier may be empirically explained by $A^{1,3}$ strain in the developing enolate **S6**.

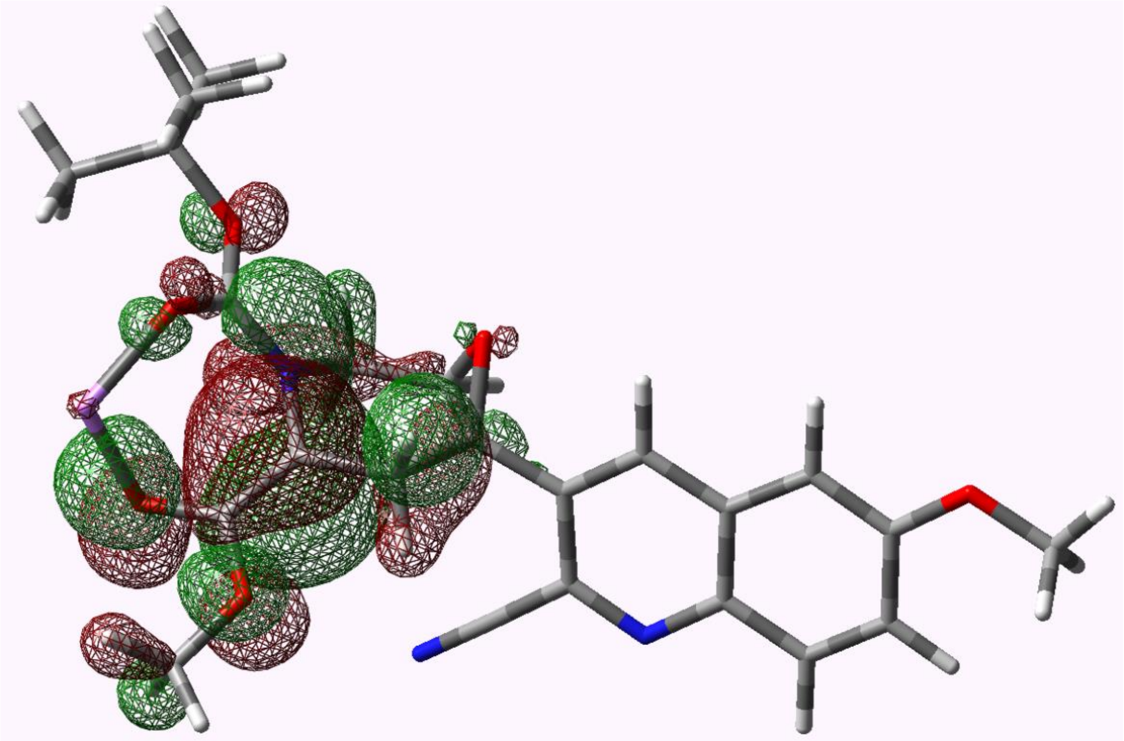

Figure S1. The enolate **14** HOMO, magnified from Scheme 2A, was calculated using M062X/6-31+G(2df,p) with simulated *tert*-butanol as solvent and lithium counterion.

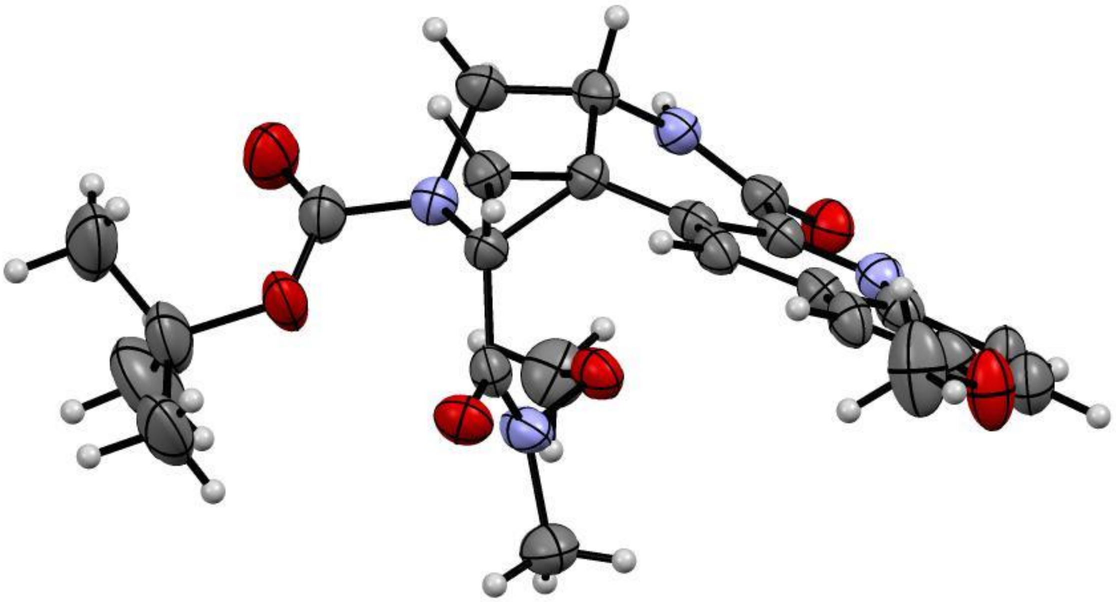

Figure S2. X-ray crystal structure of the Weinreb amide **18**, magnified from Scheme 2B.

12	*S*	*R*	*S*	*R*	*S*	*R*	*S*	*R*	*S*	*R*	*S*	*R*	*S*	*R*	*S*	*R*
13	*R*	*R*	*S*	*S*	*R*	*R*	*S*	*S*	*R*	*R*	*S*	*S*	*R*	*R*	*S*	*S*
14	*S*	*S*	*S*	*S*	*R*	*R*	*R*	*R*	*S*	*S*	*S*	*S*	*R*	*R*	*R*	*R*
15	*R*	*R*	*R*	*R*	*R*	*R*	*R*	*R*	*S*	*S*	*S*	*S*	*S*	*S*	*S*	*S*
17	*R*	*R*	*R*	*R*	*R*	*R*	*R*	*R*	*R*	*R*	*R*	*R*	*R*	*R*	*R*	*R*
2	-0.1	-0.2	-0.1	-0.2	0.1	-0.2	0.0	-0.1	0.2	0.4	0.3	-0.3	0.2	0.1	0.4	-0.3
3	-4.2	-4.2	-4.3	-4.2	-4.4	-4.3	-4.3	-4.2	-3.4	-4.1	-3.9	-4.0	-3.7	-4.1	-4.0	-4.0
4	-1.0	-1.0	-0.4	-0.8	0.4	-0.5	-0.3	-0.7	-0.7	-0.4	-0.3	-1.2	-0.6	0.2	-0.3	-1.0
5	-4.1	-4.1	-4.1	-4.1	-4.0	-4.1	-4.0	-4.1	-3.0	-3.9	-3.7	-4.1	-3.4	-3.8	-3.7	-4.0
6	5.1	5.1	4.6	4.8	3.9	4.7	4.6	4.9	4.4	4.7	4.5	4.7	4.7	3.7	4.7	4.6
7	-5.3	-5.4	-5.3	-5.3	-5.2	-5.4	-5.3	-5.4	-4.0	-4.8	-5.0	-5.1	-4.6	-4.8	-5.2	-5.1
8	-2.7	-3.0	-2.8	-3.6	-2.7	-2.9	-2.7	-3.2	-5.3	-2.2	-2.8	-3.7	-4.8	-3.5	-2.6	-4.1
9	12.3	12.5	12.5	12.9	12.5	12.5	12.3	12.6	11.9	11.6	11.4	13.8	12.3	13.1	10.3	13.2
10	10.4	8.3	11.2	10.7	11.3	9.8	12.3	11.0	3.2	4.3	3.2	4.8	3.8	4.2	3.8	5.2
11	-2.3	-3.9	-4.8	-0.7	-2.7	-2.2	-3.3	-0.3	0.8	-10.9	-3.6	-3.0	0.1	-4.6	-2.2	-1.6
12	7.1	8.2	5.4	7.4	8.0	7.1	4.0	9.2	2.5	6.7	2.2	7.4	5.4	3.8	4.4	3.3
13	-10.5	-9.9	-14.4	-10.8	-16.0	-11.0	-9.6	-7.5	-1.0	-5.8	-7.6	-9.1	-5.4	-8.3	-6.2	-4.5
14	-7.1	-9.3	-12.7	-12.8	-8.7	-10.7	-6.8	-6.0	-13.9	-11.0	-16.6	-13.9	-17.8	-20.9	-13.3	-13.6
16	-0.7	1.5	-0.6	-1.2	1.5	2.6	1.8	1.6	-5.9	-17.5	-4.7	-5.1	-7.5	-7.0	-2.2	-2.8
17	-0.7	-1.5	-2.0	-1.6	-0.6	-1.5	-1.7	-1.0	2.6	1.4	1.1	2.5	2.3	2.6	1.8	2.5
19	-1.9	-2.6	-2.1	-3.0	-2.0	-2.5	-2.1	-3.1	-1.0	2.5	0.0	-0.2	-1.7	-0.7	-1.6	-0.8
20	8.4	8.5	8.5	8.6	8.4	8.6	8.5	8.8	5.4	9.1	7.3	8.8	6.5	7.3	7.1	8.4
21	7.4	7.6	6.5	9.3	7.0	6.1	6.1	7.3	5.2	-8.9	0.3	4.3	5.1	5.8	1.9	4.5
22	11.6	14.5	10.5	10.9	11.7	16.8	14.1	14.9	12.0	15.6	12.4	11.5	11.1	10.2	14.3	14.1
23	-13.4	-14.7	-12.3	-12.7	-14.8	-16.6	-14.7	-15.7	-15.2	-16.0	-14.6	-17.0	-13.6	-12.8	-16.5	-16.3
24	3.0	3.0	3.0	3.0	3.0	3.0	3.0	3.0	2.9	3.0	3.1	3.0	3.1	3.0	3.1	3.0
25	-4.9	-2.4	-1.8	-3.9	-4.6	-2.6	-2.1	-4.5	-3.3	-2.6	-2.4	-4.6	-2.8	-2.4	-2.2	-3.5
26	-4.4	-2.4	-3.7	-5.1	-4.4	-2.3	-4.7	-4.9	-4.9	-2.9	-3.2	-4.9	-3.5	-2.6	-2.9	-5.8
MAE	5.6	5.8	5.8	6.0	6.0	6.0	5.6	5.8	4.9	6.5	5.0	6.0	5.4	5.6	5.0	5.5

Table S1. Heat map of ^{13}C chemical shift differences between reported patent data[1] and 14 calculated Boltzmann-averaged diastereomers (17*R* fixed, carbons 12, 13, 14, and nitrogen 15 variable) of ocimicide A_2 (**2**). Columns designate distinct diastereomers; rows designate carbon numbering (as in patent[1]). Mean absolute error (MAE) measures the average magnitude of errors for each diastereomer. Red coloration and blue coloration represent large positive and negative chemical shift differences, respectively.

12	*S*	*R*	*S*	*R*	*S*	*R*	*S*	*R*	*S*	*R*	*S*	*R*	*S*	*R*	*S*	*R*
13	*R*	*R*	*S*	*S*	*R*	*R*	*S*	*S*	*R*	*R*	*S*	*S*	*R*	*R*	*S*	*S*
14	*S*	*S*	*S*	*S*	*R*	*R*	*R*	*R*	*S*	*S*	*S*	*S*	*R*	*R*	*R*	*R*
15	*R*	*R*	*R*	*R*	*R*	*R*	*R*	*R*	*S*	*S*	*S*	*S*	*S*	*S*	*S*	*S*
17	*S*	*S*	*S*	*S*	*S*	*S*	*S*	*S*	*S*	*S*	*S*	*S*	*S*	*S*	*S*	*S*
2	0.4	0.5	0.4	0.4	0.4	0.5	0.4	0.5	0.0	0.0	-0.2	0.0	0.8	0.7	0.8	0.7
3	-4.6	-4.7	-4.7	-4.6	-4.6	-4.7	-4.7	-4.5	-4.3	-4.3	-4.3	-4.3	-4.2	-4.6	-4.4	-4.7
4	0.6	0.9	0.7	0.7	0.6	0.7	0.8	0.7	-0.1	-0.3	-0.5	-0.3	0.5	0.9	1.0	0.9
5	-4.1	-4.1	-4.1	-4.0	-4.1	-4.1	-4.1	-3.9	-4.0	-4.0	-4.1	-4.0	-3.7	-4.1	-3.8	-4.2
6	4.4	4.2	4.2	4.2	4.3	4.3	4.2	4.2	4.3	4.6	4.7	4.6	3.9	3.9	3.7	4.0
7	-5.1	-5.1	-5.1	-5.2	-5.1	-5.1	-5.1	-5.2	-5.3	-5.3	-5.4	-5.3	-5.1	-4.9	-4.9	-5.0
8	0.5	0.2	0.1	-1.3	0.4	0.4	0.2	-1.4	-3.5	-2.7	-2.9	-2.7	-2.8	0.9	-2.0	1.2
9	7.1	6.6	6.9	6.9	7.1	6.4	6.8	6.8	12.8	12.3	12.5	12.3	5.5	5.6	5.3	5.1
10	6.4	4.9	7.0	6.4	7.5	6.6	8.2	7.3	11.6	12.3	9.8	12.3	0.5	3.2	1.8	5.0
11	-1.3	-3.1	-3.2	-1.1	-1.6	-1.2	-1.8	0.1	0.5	-3.3	-2.2	-3.3	-2.2	-4.1	-4.7	-1.4
12	5.1	6.3	3.7	7.0	6.3	5.5	2.5	6.5	7.5	4.0	7.1	4.0	3.6	4.0	4.0	4.0
13	-10.8	-9.0	-14.3	-9.6	-16.1	-10.1	-9.6	-5.9	-7.0	-9.6	-11.0	-9.6	-4.7	-9.2	-6.0	-4.3
14	-7.0	-9.8	-12.6	-14.5	-8.9	-10.8	-7.0	-9.2	-6.6	-6.8	-10.7	-6.8	-18.1	-20.3	-11.1	-11.3
16	3.3	5.0	3.5	2.3	5.6	6.4	5.9	4.8	1.3	1.8	2.6	1.8	-2.3	-1.0	2.3	3.1
17	2.3	1.8	1.3	1.8	2.1	1.6	1.2	1.7	-1.2	-1.7	-1.5	-1.7	5.7	6.3	5.3	6.4
19	-2.8	-2.8	-2.7	-3.0	-2.8	-2.9	-2.7	-3.0	-3.1	-2.1	-2.5	-2.1	-2.9	-2.7	-2.7	-3.1
20	7.5	7.6	7.5	7.7	7.5	7.5	7.5	7.5	8.7	8.5	8.6	8.5	7.6	7.8	7.8	7.6
21	1.1	2.0	0.2	3.4	0.7	0.5	-0.2	2.9	8.2	6.1	6.1	6.1	-2.3	-2.1	-8.5	-4.1
22	12.3	15.0	11.0	11.1	11.8	17.0	14.3	14.2	14.2	14.1	16.8	14.1	11.4	10.3	14.6	14.6
23	-14.3	-15.5	-13.2	-13.3	-15.7	-17.1	-15.4	-15.4	-14.8	-14.7	-16.6	-14.7	-13.6	-12.6	-16.4	-16.3
24	3.1	3.1	3.1	3.1	3.1	3.1	3.1	3.1	3.0	3.0	3.0	3.0	3.1	3.1	3.1	3.1
25	-5.8	-3.0	-2.5	-3.7	-5.2	-3.2	-2.7	-3.4	-3.7	-2.1	-2.6	-2.1	-4.4	-3.2	-2.8	-4.2
26	-4.7	-3.1	-3.9	-4.8	-4.7	-2.8	-4.8	-6.1	-5.7	-4.7	-2.3	-4.7	-5.0	-3.6	-3.1	-6.1
MAE	5.0	5.1	5.0	5.2	5.5	5.3	4.9	5.1	5.7	5.6	6.0	5.6	5.0	5.2	5.2	5.2

Table S2. Heat map of ^{13}C chemical shift differences between reported patent data[1] and 14 calculated Boltzmann-averaged diastereomers (17*S* fixed, carbons 12, 13, 14, and nitrogen 15 variable) of ocimicide A_2 (**2**). Columns designate distinct diastereomers; rows designate carbon numbering (as in patent[1]). Mean absolute error (MAE) measures the average magnitude of errors for each diastereomer. Red coloration and blue coloration represent large positive and negative chemical shift differences, respectively.

Table S3: Comparison of ^{1}H and ^{13}C NMR data for ocimicide A$_2$ (**2**)[1] and the secondary amine **21** (CD_3OD, patent numbering).

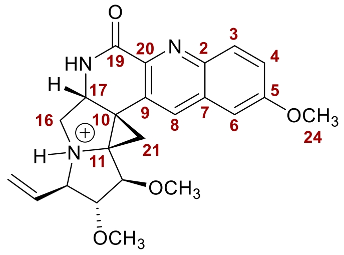

ocimicide A$_2$ (**2**)

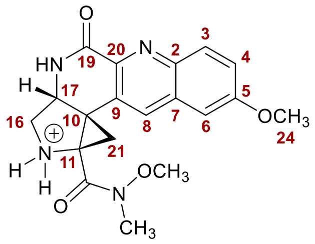

21

Position	δH (**2**)	δH (**21**)	δC (**2**)	δC (**21**)
2	—	—	145.1	143.5
3	8.12 (d, 6.8)	8.14 (d, 9.3)	133.1	131.2
4	7.33 (d, 6.8)	7.51 (d, 9.2)	122.4	126.2
5	—	—	159.9	161.6
6	7.09	7.34	108.5	106.1
7	—	—	128.9	132.3
8	8.48	8.25	131.4	134.7
9	—	—	142.3	126.7
10	—	—	33.1	38.5
11	—	—	54.1	54.5
16	2.55 (dd, 16.8, 9.2)	3.86 (d, 13.4)	48.5	50.5
	2.47 (dd, 16.8, 4.4)	3.73 (dd, 13.4, 4.6)		
17	4.17 (dd, 9.2, 4.4)	4.83 (d, 4.6)	56.0	55.8
19	—	—	168.4	164.5
20	—	—	153.8	143.6
21	0.51 (d, 8.5)	3.04 (d, 9.0)	15.2	14.9
	0.46 (d, 8.5)	2.36 (d, 9.0)		
24	3.85	3.97	56.9	56.5

Table S4: Calculated and reported ^{1}H NMR chemical shift values for 19-(*E*)-hunteracine (**20**).[2] Calculation methods: Gas-phase geometry optimization using B3LYP/6-31+G(d,p); ^{1}H NMR chemical shift values using modified WC04/6-31G(d).[3]

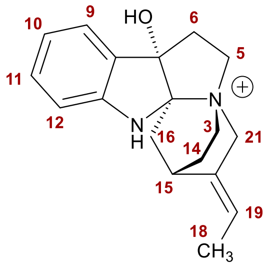

19-(E)-hunteracine (**20**)

Proton	Reported ^{1}H δ (ppm)	Calculated ^{1}H δ (ppm)	Δδ (ppm)	|Δδ| (ppm)
9	7.30	7.56	–0.26	0.26
11	7.20	7.43	–0.23	0.23
10	6.92	6.97	–0.05	0.05
12	6.75	6.75	0.00	0.00
19	5.50	5.67	–0.17	0.17
21α	4.55	4.24	0.31	0.31
21β	3.95	3.45	0.50	0.50
3α	3.75	3.60	0.15	0.15
5α	3.50	3.20	0.30	0.30
5β	3.37	3.03	0.34	0.34
15	3.37	2.91	0.46	0.46
3β	3.00	3.13	–0.13	0.13
16α	2.71	2.57	0.14	0.14
6α	2.63	2.55	0.08	0.08
6β	2.50	2.36	0.14	0.14
14α	2.42	2.04	0.38	0.38
16β	2.07	1.73	0.34	0.34
14β	1.98	1.90	0.08	0.08
18	1.74	1.81	–0.07	0.07

MAE (Mean absolute error) = 0.22 ppm

Table S5: Calculated and reported ^{13}C NMR chemical shift values for 19-(*E*)-hunteracine (**20**).[2] Calculation methods: Gas-phase geometry optimization using B3LYP/6-31+G(d,p); ^{13}C NMR chemical shift values using modified WC04/6-31G(d).[4]

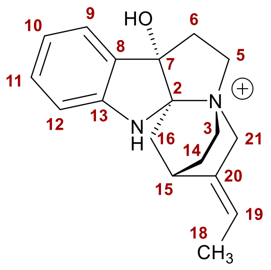

19-(E)-hunteracine (**20**)

Carbon	Reported ^{13}C δ (ppm)	Calculated ^{13}C δ (ppm)	Δδ (ppm)	|Δδ| (ppm)
13	147.3	149.9	–2.6	2.6
8	133.2	131.1	2.1	2.1
20	131.7	131.4	0.3	0.3
11	131.2	134.9	–3.7	3.7
9	123.9	127.2	–3.3	3.3
10	122.4	121.9	0.5	0.5
19	119.8	124.8	–5.0	5.0
12	112.2	111.4	0.8	0.8
2	101.5	96.2	5.3	5.3
7	88.3	82.6	5.7	5.7
21	60.1	55.9	4.2	4.2
5	57.6	53.8	3.8	3.8
3	53.7	50.1	3.6	3.6
6	43.2	41.8	1.4	1.4
16	34.5	32.7	1.8	1.8
15	28.0	24.0	4.0	4.0
14	24.5	24.3	0.2	0.2
18	12.9	14.5	–1.6	1.6

MAE (Mean absolute error) = 2.8 ppm

Table S6: Experimental and calculated ^{1}H NMR chemical shift values for the Weinreb amide **21**. Calculation methods: Gas-phase geometry optimization using B3LYP/6-31+G(d,p); ^{1}H NMR chemical shift values using modified WC04/6-31G(d).[5]

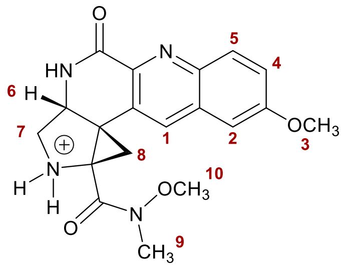

21

Proton	Experimental ^{1}H δ (ppm)	Calculated ^{1}H δ (ppm)	Δδ (ppm)	|Δδ| (ppm)
1	8.25	7.97	0.28	0.28
5	8.14	8.23	–0.09	0.09
4	7.51	7.43	0.08	0.08
2	7.34	6.90	0.44	0.44
6	4.83	4.26	0.57	0.57
3	3.97	3.99	–0.02	0.02
7α	3.86	3.56	0.30	0.30
7β	3.73	3.38	0.35	0.35
10	3.71	3.87	–0.16	0.16
9	3.05	2.81	0.24	0.24
8α	3.04	2.89	0.15	0.15
8β	2.36	1.91	0.45	0.45

MAE (Mean absolute error) = 0.26 ppm

Table S7: Experimental and calculated ^{13}C NMR chemical shift values for the Weinreb amide **21**. Calculation methods: Gas-phase geometry optimization using B3LYP/6-31+G(d,p); ^{13}C NMR chemical shift values using modified WC04/6-31G(d).[6]

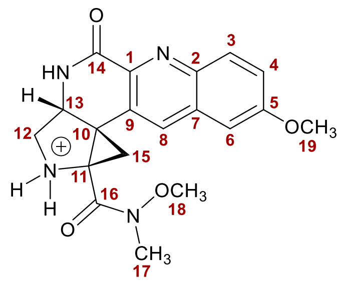

21

Carbon	Experimental ^{13}C δ (ppm)	Calculated ^{13}C δ (ppm)	Δδ (ppm)	|Δδ| (ppm)
14	164.5	170.1	–5.6	5.6
16	162.9	171.8	–8.9	8.9
5	161.6	163.9	–2.3	2.3
1	143.6	145.4	–1.8	1.8
2	143.5	145.9	–2.4	2.4
8	134.7	138.1	–3.4	3.4
7	132.3	133.5	–1.2	1.2
3	131.2	136.6	–5.4	5.4
9	126.7	126.4	0.3	0.3
4	126.2	125.8	0.4	0.4
6	106.1	102.2	3.9	3.9
18	63.1	57.9	5.2	5.2
19	56.5	53.9	2.6	2.6
13	55.8	51.9	3.9	3.9
11	54.5	47.9	6.6	6.6
12	50.5	52.6	–2.1	2.1
10	38.5	35.7	2.8	2.8
17	33.4	31.6	1.8	1.8
15	14.9	13.2	1.7	1.7

MAE (Mean absolute error) = 3.3 ppm

General Experimental Methods.

Parasite ring stage synchronization. A typical parasite culture is not synchronous and has multiple stages of the parasite: ring, trophozoite and schizont stage. An asynchronous *P. falciparum* culture was treated with 5% Sorbitol (Sigma) for 10 min at 37 °C followed by washes with complete RPMI in order to obtain a ring stage synchronized culture of the parasite.

SYBR Green-Based Parasite Growth Assay. This proliferation assay was adapted from the malaria SYBR Green I-based fluorescence assay.[7] The intermediates to be tested were added to a 96-well plate with final concentrations of 100 nM, 500 nM, 2 µM/5 µM. A highly synchronized early ring stage parasite culture was added to the plate containing conjugate. Controls were performed using non-infected erythrocytes, infected erythrocytes without conjugate, and infected erythrocytes treated with 2.5 µg/mL and 0.035 µg/mL of blasticidin. Plates were incubated for 72 and 96 h at 37 °C in a gas chamber. After 72 h and 96 h, erythrocytes were lysed with 20 mM Tris (pH 7.5), 5 mM EDTA, 0.008% saponin, 0.08% Triton-X 100, 1× SYBR Green I and incubated for 1 h in the dark at room temperature. Plates were read at 497/520 nm on a Synergy MX, Biotek fluorescent plate reader. The percent inhibition was calculated with no treatment as no inhibition and Blasticidin 2.5 µg/mL treatment as 100% inhibition.

NMR Calculations. Following the protocol reported by Hoye and co-workers,[8] structures were generated in GaussView for all diastereomers of protonated ocimicide A_2 (**2**) (at nitrogen 15 and carbons 12, 13, 14 and 17 based on patent numbering[1]). This produced 32 initial structures, which were imported into BOSS[9] and subjected to a conformational search. Rotamers within 5.02 kcal/mol of the lowest energy structure (8–30 conformers for each diastereomer) were advanced to density functional theory geometry optimization [gas phase, B3LYP/6-31+G(d,p)] in Guassian 09[10]. Geometry optimized conformers were confirmed as real local-minima by the absence of imaginary frequencies. The chemical shifts of the optimized conformers were calculated at the modified[3] WC04/6-31G(d) level of theory in methanol. Cartesian coordinates (numbering is unique for each conformer, and does not correspond to patent numbering), energy values (in A.U.), and NMR data [^{1}H and ^{13}C (ppm, δ scale); numbering is consistent with patent[1]] are presented for each unique conformer (supplementary appendix).

General Experimental Procedures. All reactions were performed in single-neck, flame-dried, round-bottomed flasks fitted with rubber septa under a positive pressure of argon, unless otherwise noted. Air and moisture-sensitive liquids were transferred via syringe or stainless steel cannula, or were handled in a nitrogen-filled drybox (working oxygen level <1 ppm). Organic solutions were concentrated by rotary evaporation at 30–33 °C. Flash-column chromatography was performed as described by Still et al,[11] employing silica gel (60 Å, 40–63 µm particle size) purchased from Sorbent Technologies (Atlanta, GA). Analytical thin-layered chromatography (TLC) was performed using glass plates precoated with silica gel (1.0 mm, 60 Å pore size) impregnated with a fluorescent indicator (254 nm). TLC plates were visualized by

exposure to ultraviolet light (UV) or/and submersion in aqueous ceric ammonium molybdate solution (CAM), acidic *p*-anisaldehyde (PAA), or aqueous potassium permanganate solution ($KMnO_4$), followed by brief heating on a hot plate (120 °C, 10–15 s).

Materials. Commercial solvents and reagents were used as received with the following exceptions. Benzene, dichloromethane, ether, and *N*,*N*-dimethylformamide were purified according to the method of Pangborn et al.[12] Triethylamine was distilled from calcium hydride under an atmosphere of nitrogen immediately before use. Tetrahydrofuran was distilled from sodium–benzophenone under an atmosphere of nitrogen immediately before use. Methanol was distilled from magnesium methoxide under an atmosphere of nitrogen immediately before use. 4 Å Molecular sieves were activated by heating overnight *in vacuo* (200 °C, 200 mTorr), stored in a gravity oven (120 °C), and were flame-dried *in vacuo* (100 mTorr) immediately before use. Toluene was purified according to the method of Pangborn et al.,[12] stored over 4 Å molecular sieves, deoxygenated by sparging with dry nitrogen for 15 min, and was stored in a nitrogen-filled drybox before use. *N,N*-Dimethylformamide was deoxygenated by purging with nitrogen for 30 min and dried over 3 Å molecular sieves for 12 h prior to use. 2,2,6,6-Tetramethylpiperidine was distilled from calcium hydride and stored under nitrogen. *N*-Bromosuccinimide was recrystallized from water.[13] 2-Cyano-6-methoxyquinoline[14] and *N*,*O*-dimethylhydroxylamine[15] were prepared according to published procedures.

Cell Culture and Materials. *Plasmodium falciparum strain* 3D7 was used and cultured by the method of Trager and Jensen[16] by using a gas mixture of 3% O_2, 3% CO_2, and 94% N_2. Complete medium (CM) used for propagation of *P. falciparum* cultures consists of RPMI medium 1640 supplemented with 30 mg/L hypoxanthine (Sigma), 25 mM Hepes (Sigma), 0.225% NaHCO3 (Sigma), 0.5% Albumax I (Life Technologies, Grand Island, NY), and 10 μg/mL gentamycin (Life Technologies). Blasticidin S was purchased from Invitrogen.

Instrumentation. Proton nuclear magnetic resonance spectra (^1H NMR) were recorded at 400 or 500 MHz at 24 °C, unless otherwise noted. Chemical shifts are expressed in parts per million (ppm, δ scale) downfield from tetramethylsilane and are referenced to residual protium in the NMR ($CDCl_3$, δ 7.26; $(CD_3)_2SO$, δ 39.5). Data are represented as follows: chemical shift, multiplicity (s = singlet, d = doublet, t = triplet, q = quartet, m = multiplet and/or multiple resonances, br = broad, app = apparent), integration, coupling constant in Hertz, and assignment. Proton-decoupled carbon nuclear magnetic resonance spectra (^{13}C NMR) were recorded at 100 or 125 MHz at 24 °C, unless otherwise noted. Chemical shifts are expressed in parts per million (ppm, δ scale) downfield from tetramethylsilane and are referenced to the carbon resonances of the solvent [$CDCl_3$, δ 77.0; $(CD_3)_2SO$, δ 39.5]. Heteronuclear single quantum correlation (HSQC) and heteronuclear multiple bond correlation (HMBC) spectra were recorded at 400/500 MHz and 100/125 MHz at 24 °C, unless otherwise noted. Fluorine nuclear magnetic resonance spectra (^{19}F NMR) were recorded at 282 or 375 MHz at 24 °C, unless otherwise noted. Chemical shifts are expressed in parts per million (ppm, δ scale), and are referenced indirectly to the ^1H frequency of $CHCl_3$. Attenuated total reflectance Fourier transform

infrared spectra (ATR-FTIR) were obtained using a Thermo Electron Corporation Nicolet 6700 FTIR spectrometer referenced to a polystyrene standard. Data are represented as follows: frequency of absorption (cm^{-1}), intensity of absorption (s = strong, m = medium, w = weak, br = broad). High-resolution mass spectrometry (HRMS) data were obtained using a Waters UPLC/HRMS instrument equipped with a dual API/ESI high-resolution mass spectrometry detector and photodiode array detector. Unless otherwise noted, samples were eluted over a reverse-phase C_{18} column (1.7 µm particle size, 2.1 × 50 mm) with a linear gradient of 5% acetonitrile–water containing 0.1% formic acid→95% acetonitrile–water containing 0.1% formic acid over 4 min, followed by 100% acetonitrile containing 0.1% formic acid for 1 min, at a flow rate of 800 µL/min.

Synthetic Procedures

*Synthesis of the enaminone **S2**:*

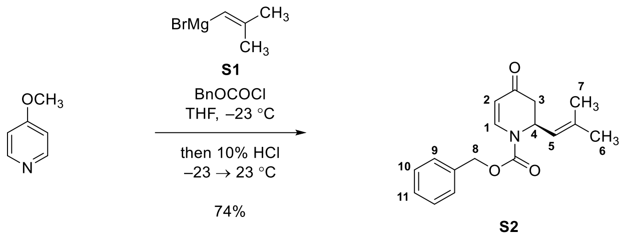

4-Methoxypyridine (10.0 mL, 98.5 mmol, 1 equiv) was added via syringe to a solution of 2-methyl-1-propenylmagnesium bromide (**S1**) in tetrahydrofuran (0.50 M, 197 mL, 98.5 mmol, 1.00 equiv) at 24 °C. The reaction mixture was cooled to –23 °C. Benzyl chloroformate (15.5 mL, 108 mmol, 1.10 equiv) was added via syringe to the cold reaction mixture. Upon completion of the addition, the mixture was stirred for 1 h at –23 °C. Aqueous hydrochloric acid solution (10% v/v, 100 mL) was added via syringe to the cold reaction mixture. The reaction mixture was allowed to warm over 30 min to 24 °C, with stirring. The warmed reaction mixture was stirred for 30 min at 24 °C. The product mixture was diluted with ethyl acetate (500 mL). The diluted product mixture was transferred to a separatory funnel that had been charged with saturated aqueous sodium chloride solution (300 mL) and the layers that formed were separated. The aqueous layer was extracted with ethyl acetate (2 × 300 mL). The organic layers were combined and the combined organic layers were dried over sodium sulfate. The dried solution was filtered and the filtrate was concentrated. The residue obtained was dissolved in dichloromethane (20 mL). Silica gel (25.0 g) was added and the suspension was concentrated to afford a free-flowing powder. The dried powder was transferred to a column of silica gel. Purification by flash-column chromatography (eluting with 30% ethyl acetate–hexanes) afforded the enaminone **S2** as a pale, yellow solid (20.9 g, 74%).

R_f = 0.44 (30% ethyl acetate–hexanes, UV). ^{1}H NMR (400 MHz, $CDCl_3$): δ 7.80 (d, 1H, H_1), 7.42–7.32 (m, 5H, H_9, H_{10}, H_{11}), 5.41–5.36 (m, 1H, H_5), 5.34 (d, 1H, J = 8.4 Hz, H_2), 5.32–5.24 (m, 2H, H_4, H_8), 5.20 (d, 1H, J = 12.0 Hz, H_8), 2.91 (dd, 1H, J = 16.0, 6.4 Hz, H_3), 2.32 (d, 1H, J = 16.0 Hz, H_3), 1.66 (br s, 3H, H_6), 1.63 (br s, 3H, H_7). ^{13}C NMR (125 MHz, $CDCl_3$): δ 192.9 (C), 152.5 (C), 142.0 (CH), 136.8 (C), 134.9 (C), 128.7 (CH), 128.7 (CH), 128.4 (CH), 119.5 (CH), 107.1 (CH), 69.0 (CH_2), 51.8 (CH), 41.7 (CH_2), 25.7 (CH_3), 17.9 (CH_3). IR (ATR-FTIR), cm^{-1}: 1721 (s), 1667 (s), 1600 (s), 1382 (m), 1321 (s), 1283 (s), 1257 (m), 1224 (m), 1192 (s), 1110 (m), 1084 (m), 1007 (m), 759 (m), 698 (m). HRMS-CI (m/z): $[M + H]^+$ calculated for $C_{17}H_{20}NO_3$, 286.1438; found, 286.1438.

*Synthesis of the vinylogous amide **S3**:*

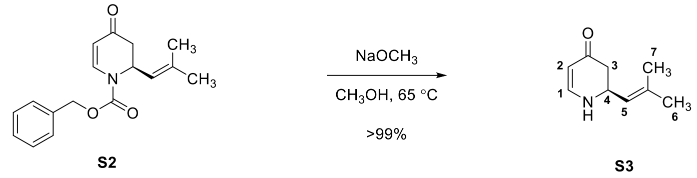

Sodium methoxide (1.90 g, 35.1 mmol, 10.0 equiv) was added to a solution of the enaminone **S2** (1.00 g, 3.51 mmol, 1 equiv) in methanol (35 mL) in a 100-mL flask that had been fused to a Teflon-coated valve at 24 °C. The reaction vessel was sealed and the sealed vessel was placed in an oil bath that had been preheated to 65 °C. The reaction mixture was stirred and heated for 12 h at 65 °C. The reaction vessel was removed from the oil bath and the product mixture was allowed to cool over 10 min to 24 °C. The cooled product mixture was concentrated to dryness to afford a brown residue. The residue obtained was diluted with ethyl acetate (200 mL). The diluted product mixture was transferred to a separatory funnel that had been charged with water (100 mL) and the layers that formed were separated. The aqueous layer was extracted with ethyl acetate (3 × 150 mL). The organic layers were combined and the combined organic layers were dried over sodium sulfate. The dried solution was filtered and the filtrate was concentrated. The residue obtained was purified by flash-column chromatography (eluting with 30% ethyl acetate–hexanes initially, grading to 20% methanol–ethyl acetate) to afford the vinylogous amide **S3** as a yellow solid (529 mg, >99%).

R_f = 0.56 (10% methanol–ethyl acetate, UV, $KMnO_4$). ^{1}H NMR (500 MHz, $CDCl_3$): δ 7.15 (t, 1H, *J* = 7.0 Hz, H_1), 5.28–5.23 (m, 1H, H_5), 5.03 (d, 1H, *J* = 7.5 Hz, H_2), 6.67 (br s, 1H, NH), 4.47–4.40 (m, 1H, H_4), 2.45 (dd, 1H, *J* = 16.0, 14.0 Hz, H_3), 2.34 (dd, 1H, *J* = 16.5, 5.0 Hz, H_3), 1.75 (s, 3H, H_6), 1.69 (s, 3H, H_7). ^{13}C NMR (125 MHz, $CDCl_3$): δ 193.0 (C), 151.1 (CH), 137.4 (C), 123.0 (CH), 99.1 (CH), 51.8 (CH), 42.5 (CH_2), 25.7 (CH_3), 18.1 (CH_3). IR (ATR-FTIR), cm^{-1}: 3232 (br), 3023 (w), 2970 (w), 2930 (w), 1617 (m), 1564 (s), 1401 (w), 1320 (w), 1278 (w), 1236 (m), 1200 (m), 765 (w). HRMS-CI (m/z): $[M + H]^+$ calculated for $C_9H_{14}NO$, 152.1070; found, 152.1071.

*Synthesis of the carbamate **S4**:*

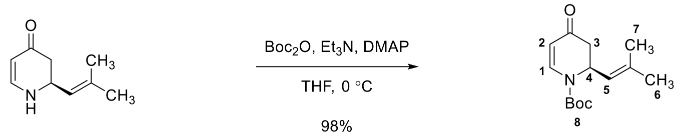

4-Dimethylaminopyridine (42.8 mg, 350 µmol, 0.10 equiv) was added to a solution of the enaminone **S3** (529 mg, 3.50 mmol, 1 equiv) in tetrahydrofuran (7.0 mL) at 24 °C. The reaction mixture was cooled to 0 °C. Triethylamine (2.93 mL, 21.0 mmol, 6.00 equiv) was added dropwise via syringe to the reaction mixture. Upon completion of the addition, the reaction mixture was stirred for 10 min at 0 °C. Di-*tert*-butyl dicarbonate (965 µL, 4.20 mmol, 1.20 equiv) was added dropwise via syringe to the cold reaction mixture. Upon completion of the addition, the reaction mixture was stirred for 4 h at 0 °C. The product mixture was diluted with ethyl acetate (50 mL). The diluted product mixture was transferred to a separatory funnel that had been charged with water (100 mL) and the layers that formed were separated. The aqueous layer was extracted with ethyl acetate (3 × 50 mL). The organic layers were combined and the combined organic layers were dried over sodium sulfate. The dried solution was filtered and the filtrate was concentrated to afford the carbamate **S4** was a pale, yellow solid (859 mg, 98%).

R_f = 0.43 (20% ethyl acetate–pentane, $KMnO_4$). ^{1}H NMR (400 MHz, $CDCl_3$): δ 7.76 (d, 1H, J = 8.4 Hz, H_1), 5.39–5.34 (m, 1H, H_5), 5.29 (dd, 1H, J = 8.8, 1.6 Hz, H_2), 5.25–5.18 (m, 1H, H_4), 2.89 (dd, 1H, J = 16.0, 6.4 Hz, H_3), 2.29 (dt, 1H, J = 16.2, 1.6 Hz, H_3), 1.71 (d, 3H, J = 1.3 Hz, H_6), 1.67 (d, 3H, J = 1.4 Hz, H_7), 1.51 (s, 9H, H_8). ^{13}C NMR (100 MHz, $CDCl_3$): δ 193.2 (C), 151.2 (C), 142.6 (CH), 136.0 (C), 120.0 (CH), 106.1 (CH), 83.2 (C), 51.5 (CH), 41.8 (CH_2), 28.1 (CH_3), 25.6 (CH_3), 18.1 (CH_3). IR (ATR-FTIR), cm^{-1}: 2978 (w), 1721 (m), 1672 (m), 1600 (m), 1327 (s), 1289 (m), 1153 (s), 1006 (w), 856 (w), 770 (m). HRMS-CI (m/z): $[M + H]^+$ calculated for $C_{14}H_{22}NO_3$, 252.1594; found, 252.1599.

*Synthesis of the vinyl triflate **9**:*

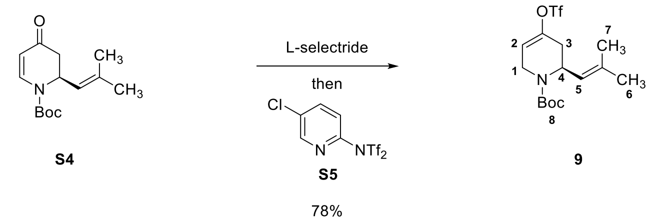

A solution of lithium tri-*sec*-butylborohydride in tetrahydrofuran (1.0 M, 67.0 mL, 67.0 mmol, 1.05 equiv) was added dropwise over 15 min via syringe pump to a solution of the carbamate **S4** [16.0 g, 63.8 mmol, 1 equiv, dried by azeotropic distillation with benzene (10 mL)] and Comins' reagent (**S5**, 27.6 g, 70.2 mmol, 1.10 equiv) in tetrahydrofuran (320 mL) at −78 °C. Upon completion of the addition, the reaction mixture was stirred for 1 h at −78 °C. The cold reaction mixture was warmed over 30 min to 24 °C. The warmed reaction mixture was stirred for 2.5 h at 24 °C. The warmed product mixture was transferred to a separatory funnel that had been charged with aqueous sodium hydroxide solution (10%, 500 mL) and ethyl acetate (500 mL). The layers that formed were separated. The aqueous layer was extracted with ethyl acetate (2 × 400 mL). The organic layers were combined and the combined organic layers were washed sequentially with 1 N aqueous sulfuric acid solution (300 mL), saturated aqueous sodium bicarbonate solution (300 mL), and saturated aqueous sodium chloride solution (300 mL). The organic layer was dried over sodium sulfate and the dried solution was filtered. The filtrate was concentrated and the residue obtained was suspended in dichloromethane (50 mL). The heterogeneous mixture was filtered through a pad of celite. The filtrate was concentrated and the residue obtained was purified by flash-column chromatography (eluting with 10% acetone–pentane) to afford a sample of the vinyl triflate **9** contaminated with reagent-derived byproducts. Further purification of this sample by flash-column chromatography (eluting with 5% ethyl acetate-dichloromethane) provided the vinyl triflate **9** as a yellow solid (19.2 g, 78%).

R_f = 0.78 (2% ethyl acetate–dichloromethane, UV, $KMnO_4$). ^{1}H NMR (600 MHz, $CDCl_3$): δ 5.77 (br s, 1H, H_2), 5.30–5.10 (m, 2H, H_4, H_5), 4.41 (d, 1H, J = 17.4 Hz, H_1), 3.61 (d, 1H, J = 18.0 Hz, H_1), 2.88–2.80 (m, 1H, H_3), 2.09 (dd, 1H, J = 16.8, 1.2 Hz, H_3), 1.72 (s, 3H, H_6), 1.71 (s, 3H, H_7), 1.47 (s, 9H, H_8). ^{13}C NMR (125 MHz, $CDCl_3$): δ 155.7 (C), 145.7 (C), 137.5 (C), 121.0 (CH), 118.4 ($J_{C\text{-}F}$ = 318.6 Hz), 115.3 (CH), 80.4 (C), 46.7 (CH), 38.6 (CH_2), 33.5 (CH_2), 28.4 (CH_3), 25.7 (CH_3), 18.4 (CH_3). ^{19}F NMR (471 MHz, $CDCl_3$): δ −73.9. IR (ATR-FTIR), cm^{-1}: 2978 (w), 1694 (m), 1408 (m), 1367 (m), 1245 (m), 1205 (s), 1169 (m), 1140 (s), 1111 (m), 1060 (s), 868 (s), 767 (w), 714 (w), 617 (m), 521 (w). HRMS-CI (m/z): $[M + H - Boc]^+$ calculated for $C_{10}H_{15}F_3NO_3S$, 286.0720; found, 286.0723.

*Synthesis of 2-cyano-6-methoxy-3-(trimethylstannyl)quinoline (**10**):*

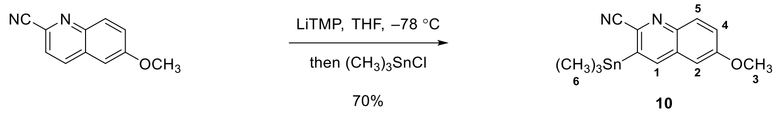

A solution of *n*-butyllithium in hexanes (2.49 M, 3.93 mL, 9.77 mmol, 1.20 equiv) was added dropwise via syringe to a solution of 2,2,6,6-tetramethylpiperidine (1.52 mL, 8.96 mmol, 1.10 equiv) in tetrahydrofuran (25 mL) at −78 °C. The resulting solution was warmed to 0 °C. The warmed solution was stirred at 0 °C for 30 min, and then was cooled to −78 °C. A separate flask was charged with 2-cyano-6-methoxyquinoline [1.50 g, 8.13 mmol, 1 equiv, dried by azeotropic distillation with benzene (3 × 5.0 mL)]. The dried residue was dissolved in tetrahydrofuran (30 mL) and the resulting solution was cooled to −78 °C. The lithium tetramethylpiperidide solution prepared above was transferred via cannula over 5 min to the flask containing 2-cyano-6-methoxyquinoline at −78 °C. The reaction mixture was stirred for 2 h at −78 °C. A solution of trimethyltin chloride (1.95 g, 9.78 mmol, 1.20 equiv) in tetrahydrofuran (6.0 mL) was then added dropwise via cannula to the cold reaction mixture. The resulting solution was stirred for 1 h at −78 °C. The product mixture was allowed to warm over 1 h to 24 °C. The warmed product mixture was concentrated to dryness (***Caution:*** this operation should be performed in a well-ventilated fume hood). The residue obtained was purified by flash-column chromatography (eluting with 5% ethyl acetate−hexanes) to yield 2-cyano-6-methoxy-3-(trimethylstannyl)quinoline (**10**) as a pale, yellow solid (1.97 g, 70%).

R_f = 0.64 (20% ethyl acetate–pentane, UV, $KMnO_4$). ^1H NMR (500 MHz, $CDCl_3$): δ 8.20 (s, 1H, H_1), 7.97 (d, 1H, J = 9.5 Hz, H_5), 7.41 (dd, 1H, J = 9.5, 3.0 Hz, H_4), 7.04 (d, 1H, J = 2.5 Hz, H_2), 3.95 (s, 3H, H_3), −0.53 (s, 9H, H_6). ^{13}C NMR (125 MHz, $CDCl_3$): δ 159.6 (C), 144.1 (C), 143.5 (CH), 137.8 (C), 136.8 (C), 131.1 (CH), 130.1 (C), 124.2 (CH), 119.2 (C), 104.2 (CH), 55.7 (CH_3), −9.00 (CH_3). IR (ATR-FTIR), cm^{-1}: 3056 (w), 2912 (w), 1619 (m), 1486 (s), 1406 (w), 1235 (s), 1167 (w), 1030 (m), 911 (w), 848 (m), 824 (m), 780 (m), 536 (m). HRMS-CI (m/z): $[M + H]^+$ calculated for $C_{14}H_{17}N_2OSn$, 349.0357; found, 349.0359.

*Synthesis of the cyclohexenylquinoline **11**:*

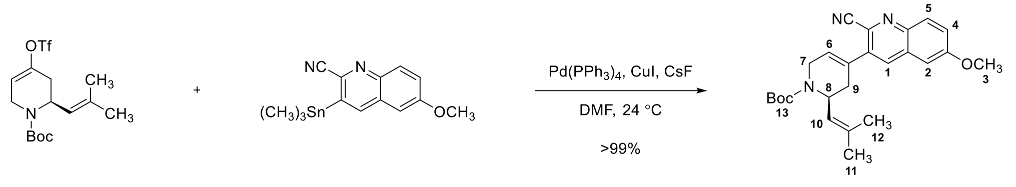

9 **10** **11**

A 100-mL round-bottomed flask fused to a Teflon-coated valve was charged with the vinyl triflate **9** (1.36 g, 3.90 mmol, 1 equiv) and 2-cyano-6-methoxy-3-(trimethylstannyl)quinoline (**10**, 1.50 g, 3.90 mmol, 1.00 equiv). Benzene (5.0 mL) was added and the solution was concentrated to dryness. This process was repeated twice. The vessel was sealed and the sealed vessel was transferred to a nitrogen-filled drybox. *N*,*N*-dimethylformamide (19.5 mL), tetrakis(triphenylphosphine)palladium (225 mg, 195 μmol, 0.05 equiv), cesium fluoride (1.18 g, 7.79 mmol, 2.00 equiv), and copper iodide (74.2 mg, 390 μmol, 0.10 equiv) were added in sequence. The vessel was sealed and the sealed vessel was removed from the drybox. The reaction mixture was stirred for 1 h at 24 °C. The product mixture was diluted with ethyl acetate (50 mL) and water (50 mL). The diluted product mixture was eluted through a pad of celite (length/diameter = 6/4 cm). The celite pad was washed sequentially with water (50 mL) and ethyl acetate (400 mL). The biphasic filtrate was transferred to a separatory funnel and the layers that formed were separated. The organic layer was washed sequentially with water (3 × 100 mL) and saturated aqueous sodium chloride solution (100 mL). The organic layer was dried over sodium sulfate. The dried solution was filtered and the filtrate was concentrated. The residue obtained was purified by flash-column chromatography (eluting with 2% acetone−dichloromethane) to afford the cyclohexenylquinoline **11** as a pale yellow solid (1.63 g, >99%).

R_f = 0.60 (10% ethyl acetate–dichloromethane, UV, $KMnO_4$). ^1H NMR (500 MHz, $CDCl_3$): δ 8.01 (d, 1H, *J* = 9.0 Hz, H_5), 7.93 (s, 1H, H_1), 7.42 (dd, 1H, *J* = 9.0 Hz, H_4), 7.05 (d, 1H, *J* = 2.5 Hz, H_2), 6.16–6.12 (m, 1H, H_6), 5.48 (d, 1H, *J* = 9.0 Hz, H_{10}), 5.26 (br s, 1H, H_8), 4.47 (d, 1H, *J* = 19.5 Hz, H_7), 3.96 (s, 3H, H_3), 3.80 (d, 1H, *J* = 19.0 Hz, H_7), 3.06–2.98 (m, 1H, H_9), 2.29 (d, 1H, *J* = 16.5 Hz, H_9), 1.78 (s, 3H, H_{12}), 1.74 (s, 3H, H_{11}), 1.51 (s, 9H, H_{13}). ^{13}C NMR (125 MHz, $CDCl_3$): δ 160.1 (C), 154.2 (C), 143.5 (C), 138.1 (C), 136.2 (C), 133.5 (CH), 131.2 (CH), 130.1 (C), 130.0 (C), 129.5 (C), 127.5 (CH), 124.3 (CH), 122.2 (CH), 117.4 (C), 104.3 (CH), 79.9 (C), 55.8 (CH_3), 46.3 (CH), 40.6 (CH_2), 34.5 (CH_2), 28.5 (CH_3), 25.8 (CH_3), 18.5 (CH_3). IR (ATR-FTIR), cm^{-1}: 2974 (w), 2930 (w), 1688 (s), 1619 (m), 1489 (m), 1452 (w), 1412 (m), 1364 (m), 1307 (w), 1233 (s), 1169 (s), 1110 (m), 1027 (m), 991 (w), 834 (m). HRMS-CI (m/z): $[M + H]^+$ calculated for $C_{25}H_{30}N_3O_3$, 420.2282; found, 420.2281.

*Synthesis of the carboxylic acid **12**:*

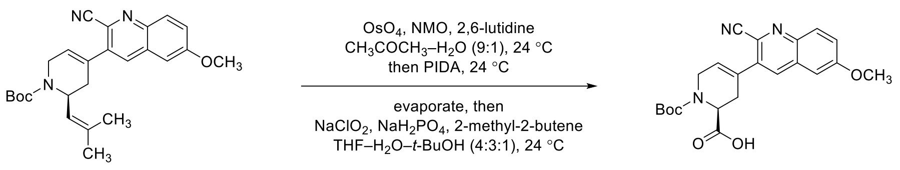

2,6-Lutidine (363 µL, 3.13 mmol, 2.00 equiv) and *N*-methylmorpholine *N*-oxide (220 mg, 1.88 mmol, 1.20 equiv) were added in sequence to a solution of the cyclohexenylquinoline **11** (656 mg, 1.57 mmol, 1 equiv) in acetone (28 mL) and water (3.1 mL) at 24 °C. A solution of aqueous osmium tetroxide (2% w/w, 491 µL, 31.3 µmol, 0.025 equiv) was added to the reaction mixture at 24 °C. Upon completion of the addition, the resulting reaction mixture was stirred for 12 h at 24 °C. (Diacetoxyiodo)benzene (605 mg, 1.88 mmol, 1.20 equiv) was then added to the reaction mixture. The resulting reaction mixture was stirred for 3 h at 24 °C. An additional portion of (diacetoxyiodo)benzene (302 mg, 0.94 mmol, 0.60 equiv) was added to the reaction mixture and the reaction mixture was stirred for 1 h at 24 °C. The reaction mixture was concentrated to remove acetone (***Caution:*** this operation should be performed in a well-ventilated fume hood). The oily residue in water was diluted with tetrahydrofuran (15.7 mL), *tert*-butyl alcohol (3.9 mL), and water (12.6 mL). 2-Methyl-2-butene (3.9 mL), monosodium phosphate (1.88 g, 15.7 mmol, 10.0 equiv), and sodium chlorite (991 mg, 11.0 mmol, 7.00 equiv) were added in sequence to the reaction mixture at 24 °C. The reaction mixture was stirred for 1 h at 24 °C. The product mixture was diluted with ether (300 mL). The diluted product mixture was transferred to a separatory funnel that had been charged with 5% aqueous sodium hydroxide solution (200 mL) and the layers that formed were separated. The organic layer was extracted with 5% aqueous sodium hydroxide solution (3 × 150 mL) and water (2 × 100 mL). The aqueous layers were combined and the combined aqueous layers were acidified to pH 4 with concentrated hydrochloric acid solution, to afford an aqueous suspension. The aqueous suspension was transferred to a separatory funnel that had been charged with ethyl acetate (300 mL) and the layers that formed were separated. The aqueous layer was extracted with ethyl acetate (3 × 300 mL). The organic layers were combined and the combined organic layers were dried over sodium sulfate. The dried solution was filtered and the filtrate was concentrated to afford the unpurified carboxylic acid **12** as a brown solid. ^{1}H NMR analysis (400 MHz, $CDCl_3$) indicated >95% conversion to the carboxylic acid **12**. The product so obtained was used directly in subsequent steps.

The carboxylic acid **12** was not amenable to purification by flash-column chromatography. Therefore, further characterization was not attempted.

*Synthesis of the bromolactone **13**:*

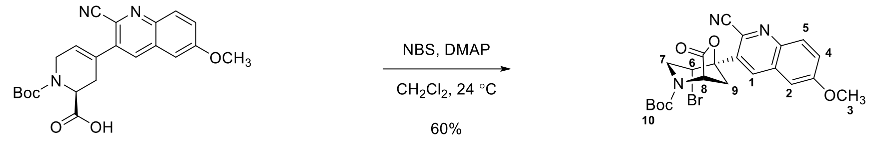

4-Dimethylaminopyridine (35.5 mg, 291 μmol, 0.19 equiv) and *N*-bromosuccinimide (311 mg, 1.75 mmol, 1.12 equiv) were added in sequence to a solution of the unpurified acid **12** [1.57 mmol, 1 equiv; assuming quantitative yield in the previous step, dried by azeotropic distillation with benzene (3 × 10 mL)] in dichloromethane (58 mL) at 24 °C. The reaction mixture was stirred for 1 h at 24 °C. The reaction mixture was concentrated to dryness and the residue obtained was purified by flash-column chromatography (eluting with 3% acetone–dichloromethane) to afford the bromolactone **13** as an off-white solid (402 mg, 60%).

Note: The bromolactone **13** was found to be unstable towards prolonged exposure to silica gel (as indicated by two-dimensional TLC analysis). Therefore, the purification step should be performed as rapidly as possible.

R_f = 0.40 (2% acetone–dichloromethane, UV, $KMnO_4$). ^{1}H NMR (500 MHz, $(CD_3)_2SO$): δ 8.65 (s, 1H, H_1), 8.11 (d, 1H, *J* = 9.0 Hz, H_5), 7.65 (dd, 1H, *J* = 9.0, 2.5 Hz, H_4), 7.60 (d, 1H, *J* = 2.5 Hz, H_2), 5.04 (br s, 1H, H_6), 4.95 (br s, 1H, H_8), 4.39 (d, 1H, *J* = 17.0 Hz, H_7), 3.97 (s, 3H, H_3), 3.85 (br s, 1H, H_7), 3.28 (d, 1H, *J* = 12.5 Hz, H_9), 2.92 (dd, 1H, *J* = 12.5, 2.5 Hz, H_9), 1.48 (s, 9H, H_{10}). ^{13}C NMR (125 MHz, $(CD_3)_2SO$): δ 170.6 (C), 160.2 (C), 153.2 (C), 143.2 (C), 136.9 (CH), 130.6 (C), 130.5 (CH), 129.2 (C), 127.1 (C), 125.7 (CH), 116.9 (C), 106.0 (CH), 86.5 (C), 81.2 (C), 56.0 (CH_3), 54.6 CH, detected by HSQC), 49.9 (CH, detected by HSQC), 48.0 (CH_2, detected by HSQC), 35.3 (CH_2), 27.8 (CH_3). IR (ATR-FTIR), cm^{-1}: 1815 (s), 1704 (s), 1619 (m), 1492 (m), 1457 (w), 1393 (s), 1367 (w), 1301 (w), 1246 (s), 1156 (s), 1083 (m), 1035 (m), 930 (w), 899 (m), 837 (m). HRMS-CI (m/z): $[M + H]^+$ calculated for $C_{22}H_{23}{}^{79/81}BrN_3O_5$, 488.0816/490.0795; found, 488.0815/490.0797.

One-pot Synthesis of the epoxide ***8:***

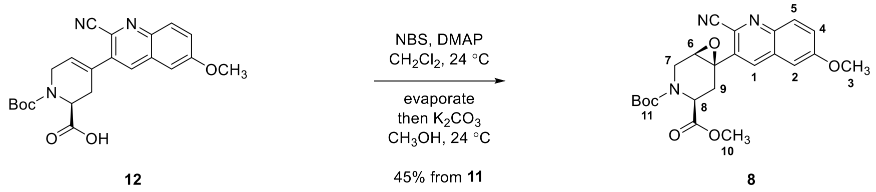

4-Dimethylaminopyridine (35.5 mg, 291 µmol, 0.19 equiv) and *N*-bromosuccinimide (311 mg, 1.75 mmol, 1.12 equiv) were added in sequence to a solution of the unpurified acid **12** [1.56 mmol, 1 equiv; assuming quantitative yield in the saponification step, dried by azeotropic distillation with benzene (3 × 10 mL)] in dichloromethane (58 mL) at 24 °C. The reaction mixture was stirred for 1 h at 24 °C. The reaction mixture was concentrated to dryness. The residue so obtained was dissolved in tetrahydrofuran (24 ml) and methanol (48 mL). Potassium carbonate (1.00 g, 7.27 mmol, 4.65 equiv) was added to the reaction mixture at 24 °C. The resulting suspension was vigorously stirred for 1 h at 24 °C. The product mixture was diluted with ethyl acetate (250 mL). The diluted product mixture was transferred to a separatory funnel that had been charged with 100 mM aqueous sodium phosphate buffer solution (pH 7, 200 mL) and the layers that formed were separated. The aqueous layer was extracted with ethyl acetate (3 × 100 mL). The organic layers were combined and the combined organic layers were dried over sodium sulfate. The dried organic solution was filtered and the filtrate was concentrated. The residue obtained was purified by flash-column chromatography (eluting with 10% acetone–dichloromethane) to afford the epoxide **8** as an off-white solid (308 mg, 45% from **11**).

R_f = 0.37 (10% ethyl acetate–dichloromethane, $KMnO_4$). ^1H NMR (400 MHz, $CDCl_3$, 2:1 mixture of rotamers): major rotamer, δ 8.24 (s, 1H, H_1), 8.03 (d, 1H, *J* = 9.2 Hz, H_5), 7.47 (dd, 1H, *J* = 9.6, 2.8 Hz, H_4), 7.09 (br s, 1H, H_2), 4.94–4.90 (m, 1H, H_8), 4.21 (dd, 1H, *J* = 15.2, 3.6 Hz, H_7), 3.96 (s, 3H, H_3), 3.97–3.89 (m, 1H, H_7), 3.81 (s, 3H, H_{10}), 3.49 (dd, 1H, *J* = 17.2, 2.8 Hz, H_6), 2.99–2.90 (m, 1H, H_9), 2.80 (dd, 1H, *J* = 14.8, 6.0 Hz, H_9), 1.52 (s, 9H, H_{11}); minor rotamer, δ 8.22 (s, 1H, H_1), 8.03 (d, 1H, *J* = 9.2 Hz, H_5), 7.47 (dd, 1H, *J* = 9.6, 2.8 Hz, H_4), 7.09 (br s, 1H, H_2), 4.72–4.68 (m, 1H, H_8), 4.17 (dd, 1H, *J* = 14.8, 3.2 Hz, H_7), 3.96 (s, 3H, H_3), 3.97–3.89 (m, 1H, H_7), 3.82 (s, 3H, H_{10}), 3.49 (dd, 1H, *J* = 17.2, 2.8 Hz, H_6), 2.99–2.90 (m, 2H, H_9), 1.46 (s, 9H, H_{11}). ^{13}C NMR (100 MHz, $CDCl_3$, 2:1 mixture of rotamers): major rotamer, δ 171.6 (C), 160.4 (C), 155.4 (C), 144.1 (C), 135.1 (C), 134.1 (CH), 131.3 (CH), 130.1 (C), 128.6 (C), 125.1 (CH), 116.3 (C), 104.5 (CH), 81.0 (C), 58.0 (C), 56.9 (CH), 55.8 (CH_3), 52.5 (CH_3), 49.9 (CH), 40.3 (CH_2), 32.3 (CH_2), 28.3 (CH_3); minor rotamer, δ 171.9 (C), 160.4 (C), 154.7 (C), 144.0 (C), 135.0 (C), 134.0 (CH), 131.3 (CH), 130.0 (C), 128.6 (C), 125.0 (CH), 116.4 (C), 104.5 (CH), 80.9 (C), 57.7 (C), 57.3 (CH), 55.8 (CH_3), 52.4 (CH_3), 51.3 (CH), 40.0 (CH_2), 32.2 (CH_2), 28.2 (CH_3). IR (ATR-FTIR), cm^{-1}: 2975 (m), 1744 (m), 1697 (s), 1620 (m), 1367 (m), 1331 (m), 1237 (s), 1168 (m), 1120 (m), 1050 (m), 1024 (w), 990 (w), 862 (m), 834 (m). HRMS-CI (m/z): $[M + H]^+$ calculated for $C_{23}H_{26}N_3O_6$, 440.1816; found, 440.1818.

*Synthesis of the alcohol **7**:*

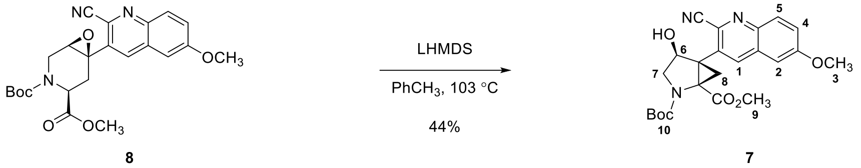

In a nitrogen-filled drybox, toluene (11 mL) and lithium bis(trimethylsilyl)amide (20.6 mg, 123 μmol, 1.10 equiv) were added in sequence to a 25-mL round-bottomed flask fused to a Teflon-coated valve that had been charged with the epoxide **8** [49.1 mg, 112 μmol, 1 equiv, dried by azeotropic distillation with benzene (4 × 1.0 mL)]. The vessel was sealed and the sealed vessel was removed from the drybox. The reaction vessel was placed in an oil bath that had been preheated to 103 °C. The reaction mixture was stirred and heated for 2 h at 103 °C. The reaction vessel was removed from the oil bath and the product mixture was allowed to cool over 1 min to 24 °C. The cooled product mixture was transferred to a separatory funnel that had been charged with saturated aqueous ammonium chloride solution (20 mL) and ethyl acetate (30 mL). The layers that formed were separated. The aqueous layer was extracted with ethyl acetate (4 × 30 mL). The organic layers were combined and the combined organic layers were dried over sodium sulfate. The dried solution was filtered and the filtrate was concentrated to afford a red viscous oil. ^{1}H NMR analysis (400 MHz, $CDCl_3$) indicated 75% conversion to the alcohol **7**. Purification by flash-column chromatography (eluting with 30% ethyl acetate−dichloromethane, grading to 50% ethyl acetate−dichloromethane, one step) afforded the alcohol **7** as an off-white solid (21.4 mg, 44%) and the recovered epoxide **8** as a pale, yellow solid (9.2 mg, 19%).

R_f = 0.26 (30% ethyl acetate–dichloromethane, UV, $KMnO_4$). ^{1}H NMR (500 MHz, $CDCl_3$): δ 8.02 (d, 1H, J = 9.0 Hz, H_5), 8.00 (s, 1H, H_1), 7.46 (dd, 1H, J = 9.0, 3.0 Hz, H_4), 7.04 (s, 1H, H_2), 4.91 (br s, 1H, H_6), 4.36 (dd, 1H, J = 12.5, 7.0 Hz, H_7), 3.96 (s, 3H, H_3), 3.63 (s, 3H, H_9), 3.51 (br s, 1H, H_7), 2.43 (d, 1H, J = 5.5 Hz, H_8), 2.25 (d, 1H, J = 6.0 Hz, H_8), 1.47 (s. 9H, H_{10}). ^{13}C NMR (150 MHz, $CDCl_3$): δ 168.3 (C), 160.2 (C), 154.7 (C), 143.4 (C), 136.7 (CH), 136.1 (C, detected by HMBC), 133.1 (C), 130.8 (CH), 130.1 (C), 124.8 (CH), 116.5 (C), 104.4 (CH), 80.9 (C), 75.9 (CH), 57.3 (CH_2), 55.8 (CH_3), 54.4 (C), 52.4 (CH_3), 47.6 (C), 28.3 (CH_3), 24.6 (CH_2). IR (ATR-FTIR), cm^{-1}:3405 (br), 2976 (m), 1706 (s), 1682 (s), 1620 (s), 1493 (m), 1392 (s), 1338 (m), 1240 (s), 1166 (s), 1090 (w), 1024 (w), 834 (m). HRMS-CI (m/z): $[M + H]^+$ calculated for $C_{23}H_{26}N_3O_6$, 440.1816; found, 440.1819.

*Synthesis of the ester **6**:*

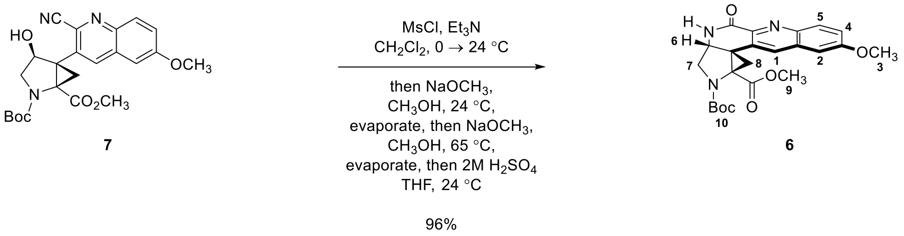

Triethylamine (1.06 mL, 7.59 mmol, 10.0 equiv) and methanesulfonyl chloride (70.1 µL, 910 µmol, 1.20 equiv) were added in sequence to a solution of the alcohol **7** [333 mg, 759 µmol, 1 equiv, dried by azeotropic distillation with benzene (10 mL)] in dichloromethane (10 mL) in a 50-mL round-bottomed flask fused to a Teflon-coated valve at 24 °C. Upon completion of the addition, the reaction mixture was stirred for 1 h at 24 °C. An additional portion of methanesulfonyl chloride (10.0 µL, 130 µmol, 0.17 equiv) was then added at 24 °C. Upon completion of the addition, the reaction mixture was stirred for 30 min at 24 °C. Sodium methoxide (150 mg, 2.78 mmol, 3.67 equiv) and methanol (5.0 mL) were added in sequence to the reaction mixture at 24 °C. The resulting mixture was stirred for 1 min at 24 °C, and then the reaction mixture was concentrated to dryness. Sodium methoxide (328 mg, 6.07 mmol, 8.00 equiv) and methanol (15 mL) were added in sequence to the reaction vessel at 24 °C. The reaction vessel was sealed and the sealed vessel was placed in an oil bath that had been preheated to 65 °C. The reaction mixture was stirred and heated for 12 h at 65 °C. The reaction mixture was concentrated to dryness and the residue obtained was dissolved in tetrahydrofuran (76 mL). Aqueous sulfuric acid solution (2 N, 15 mL) was added to the reaction mixture at 24 °C. The reaction mixture was stirred for 2 h at 24 °C. The product mixture was diluted with dichloromethane (300 mL). The diluted product mixture was poured slowly into a separatory funnel that had been charged with saturated aqueous sodium bicarbonate solution (300 mL) and the layers that formed were separated. The aqueous layer was extracted with dichloromethane (3 × 250 mL). The organic layers were combined and the combined organic layers were dried over sodium sulfate. The dried solution was filtered and the filtrate was concentrated to afford the ester **6** as an off-white solid (321 mg, 96%).

R_f = 0.45 (5% methanol–ethyl acetate, UV, $KMnO_4$). ^{1}H NMR (400 MHz, $(CD_3)_2SO$): δ 8.50 (br s, 1H, NH), 8.10 (s, 1H, H_1), 8.01 (d, 1H, J = 9.2 Hz, H_5), 7.45 (dd, 1H, J = 9.2, 2.8 Hz, H_4), 7.40 (br s, 1H, H_2), 4.55 (d, 1H, J = 5.2 Hz, H_6), 3.93 (s, 3H, H_3), 3.85 (d, 1H, J = 12.0 Hz, H_7), 3.46 (dd, 1H, J = 12.0, 5.2 Hz, H_7), 3.27 (s, 3H, H_9), 2.65 (d, 1H, J = 7.2 Hz, H_8), 2.01 (d, 1H, J = 7.2 Hz, H_8), 1.38 (s, 9H, H_{10}). ^{13}C NMR (100 MHz, $(CD_3)_2SO$): δ 166.3 (C), 161.6 (C), 158.7 (C), 155.0 (C, detected by HMBC), 144.1 (C), 142.6 (C), 131.9 (CH), 131.2 (CH), 129.6 (C), 125.9 (C), 123.2 (CH), 105.3 (CH), 80.3 (C), 55.7 (CH_3), 54.8 (C), 52.8 (CH, detected by HSQC), 51.9 (CH_2), 51.8 (CH_3), 39.4 (C, detected by HMBC), 27.8 (CH_3), 16.2 (CH_2). IR (ATR-FTIR), cm^{-1}: 3217 (br), 2979 (m), 1686 (s), 1622 (m), 1498 (m), 1369 (s), 1339 (m), 1170 (m) 833 (w). HRMS-CI (m/z): $[M + H]^+$ calculated for $C_{23}H_{26}N_3O_6$, 440.1816; found, 440.1818.

*Synthesis of the acid **17**:*

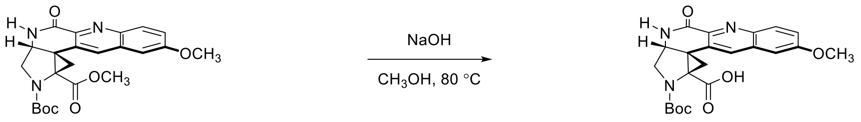

6 **17**

Aqueous sodium hydroxide solution (1.25 M, 2.3 mL) was added to a solution of the ester **6** (20.4 mg, 46.4 μmol, 1 equiv) in methanol (4.6 mL) in a 10-mL round-bottomed flask fused to a Teflon-coated valve at 24 °C. The reaction vessel was sealed and the sealed vessel was placed in an oil bath that had been preheated to 80 °C. The reaction mixture was stirred and heated for 14 h at 80 °C. The product mixture was allowed to cool over 10 minutes to 24 °C. The cooled product mixture was concentrated to remove methanol. The residue was diluted with water (8.0 mL) and the diluted mixture was acidified to pH 6 with concentrated hydrochloric acid solution. The resulting mixture was loaded onto a column of reverse-phase silica (length/diameter = 15/1 cm). The column was washed with water (20 mL) to remove inorganic salts and the washed column was dried under a stream of nitrogen. The product was then eluted from the dried reverse-phase silica column with methanol (50 mL). The methanol collected and concentrated to afford unpurified acid **17**. The product so obtained was used directly in the following step.

*Synthesis of the Weinreb amide **18**:*

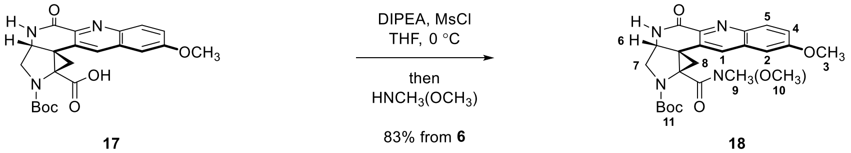

Methanesulfonyl chloride (165 µL, 2.13 mmol, 10.0 equiv) was added dropwise to a solution of the unpurified acid **17** [213 µmol, 1 equiv; assuming quantitative yield in the preceding step, dried by azeotropic distillation with benzene (3 × 4.0 mL)] and *N*,*N*-di-*iso*-propylethylamine (743 µL, 4.26 mmol, 20.0 equiv) in tetrahydrofuran (18 mL) at 0 °C. The reaction mixture was stirred for 30 min at 0 °C. *N,O*-Dimethylhydroxylamine (385 µL, 5.02 mmol, 23.6 equiv) was added to the reaction mixture at 0 °C. The reaction mixture was stirred for 3 h at 0 °C. The product mixture was diluted with water (5.0 mL). The diluted product mixture was transferred to a separatory funnel that had been charged with saturated aqueous sodium bicarbonate solution (10 mL) and dichloromethane (30 mL). The layers that formed were separated. The aqueous layer was extracted with dichloromethane (4 × 30 mL). The organic layers were combined and the combined organic layers were dried over sodium sulfate. The dried solution was filtered and the filtrate was concentrated. The residue obtained was dissolved in dichloromethane (6.0 mL), and silica gel (800 mg) was added to the diluted product mixture. The resulting suspension was concentrated to afford a free-flowing powder. The dried powder was loaded onto a column of silica gel. Flash-column chromatography (eluting with 10% methanol−ethyl acetate) afforded the Weinreb amide **18** as a white solid (82.9 mg, 83% from **6**).

R_f = 0.27 (10% methanol–ethyl acetate, UV, $KMnO_4$). ^{1}H NMR (400 MHz, $CDCl_3$): δ 8.21 (d, 1H, *J* = 9.2 Hz, H_5), 7.61 (br s, 1H, H_1), 7.37 (dd, 1H, *J* = 9.0, 3.0 Hz, H_4), 7.05 (d, 1H, *J* = 3.0 Hz, H_2), 4.68 (d, 1H, *J* = 5.5 Hz, H_6), 3.99 (d, 1H, *J* = 13.0 Hz, H_7), 3.93 (s, 3H, H_3), 3.64 (dd, 1H, *J* = 12.5, 5.5 Hz, H_7), 3.54 (br s, 3H, H_{10}), 3.60 (br s, 4H, H_9, H_8), 1.60 (d, 1H, *J* = 7.0 Hz, H_8), 1.47 (s, 9H, H_{11}). ^{13}C NMR (150 MHz, $CDCl_3$): δ 167.3 (C), 163.6 (C), 159.5 (C), 155.1 (C), 143.6 (C), 143.3 (C), 132.2 (CH), 130.1 (CH), 130.0 (C, detected by HMBC), 126.4 (C), 123.6 (CH), 104.5 (CH), 81.9 (C), 60.6 (CH_3), 57.5 (C), 55.6 (CH_3), 54.6 (CH_2), 53.7 (CH), 35.6 (C, detected by HMBC), 32.8 (CH_3), 28.2 (CH_3), 16.8 (CH_2). IR (ATR-FTIR), cm^{-1}: 3461 (br), 3232 (br), 2977 (m), 2932 (m), 1681 (s), 1621 (m), 1497 (m), 1368 (s), 1242 (m), 1170 (m), 1141 (m), 1117 (m), 1023 (m), 836 (m). HRMS-CI (m/z): $[M + H]^+$ calculated for $C_{24}H_{29}N_4O_6$, 469.2082; found, 469.2081.

*Synthesis of the amine **19**:*

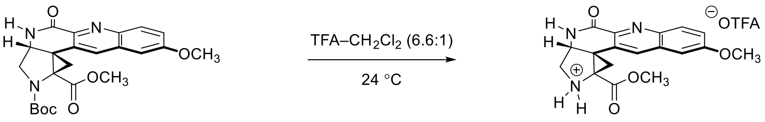

6

19

Trifluoroacetic acid (209 μL) was added to a solution of the ester **6** (6.0 mg, 13.7 μmol, 1 equiv) in dichloromethane (1.4 mL) at 24 °C. Upon completion of the addition, the reaction mixture was stirred for 12 h at 24 °C. The product mixture was concentrated to provide the amine **19** as an off white solid. ^{1}H NMR analysis (400 MHz, $CDCl_3$) indicated >95% conversion to the secondary amine **19**.

The secondary amine **19** was found to be unstable towards neutralization and purification by flash-column chromatography. Therefore, further characterization was not attempted.

Synthesis of the amine ***21****:*

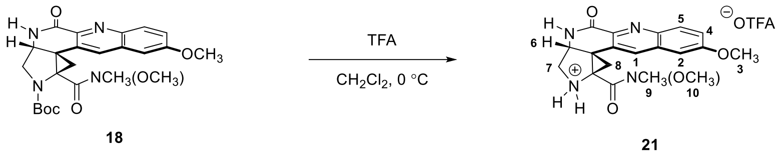

Trifluoroacetic acid (52.4 µL, 68.5 µmol, 30.0 equiv) was added to a solution of the Weinreb amide **18** (10.7 mg, 22.8 µmol, 1 equiv) in dichloromethane (200 µL) at 0 °C. Upon completion of the addition, the reaction mixture was stirred for 12 h at 0 °C. The product mixture was concentrated to provide the secondary amine **21** as an off white solid. ^{1}H NMR analysis (500 MHz, CD_3OD) indicated >95% conversion to the secondary amine **21**.

The secondary amine **21** was found to be unstable towards neutralization and purification by flash-column chromatography. Therefore, further characterization was not attempted.

^{1}H NMR (500 MHz, CD_3OD) δ 8.25 (s, 1H, H_1), 8.14 (d, *J* = 9.3 Hz, 1H, H_5), 7.51 (d, *J* = 9.2 Hz, 1H, H_4), 7.34 (s, 1H, H_2), 4.83 (d, *J* = 4.6 Hz, 1H, H_6), 3.97 (s, 3H, H_3), 3.86 (d, *J* = 13.4 Hz, 1H, H_7), 3.76 – 3.68 (m, 4H, H_7, H_{10}), 3.10 – 3.00 (m, 4H, H_8, H_9), 2.36 (d, *J* = 9.0 Hz, 1H, H_8). ^{13}C NMR (126 MHz, CD_3OD) δ 164.5 (C), 162.9 (C), 161.6 (C), 143.6 (C), 143.5 (C), 134.7 (CH), 132.3 (C), 131.2 (CH), 126.7 (C), 126.2 (CH), 106.1 (CH), 63.1 (CH_3), 56.5 (CH_3), 55.8 (CH), 54.5 (C), 50.5 (CH_2), 38.5 (C), 33.4 (CH_3), 14.9 (CH_2). ^{19}F NMR (376 MHz, CD_3OD) δ –77.20. HRMS-CI (m/z): $[M – OTFA]^+$ calculated for $C_{19}H_{21}N_4O_4$, 369.1557; found, 369.1553.

Catalog of Nuclear Magnetic Resonance Spectra.

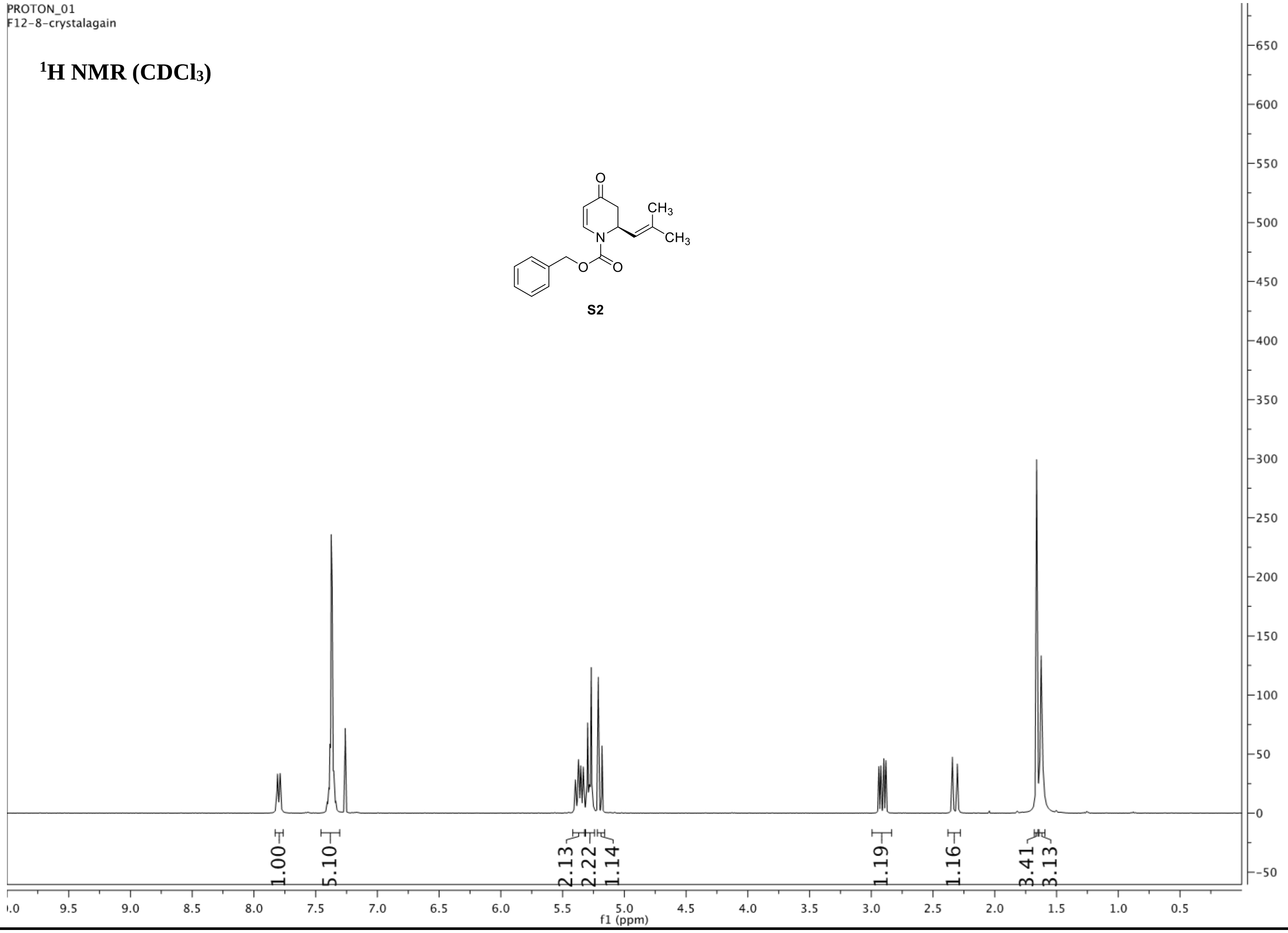

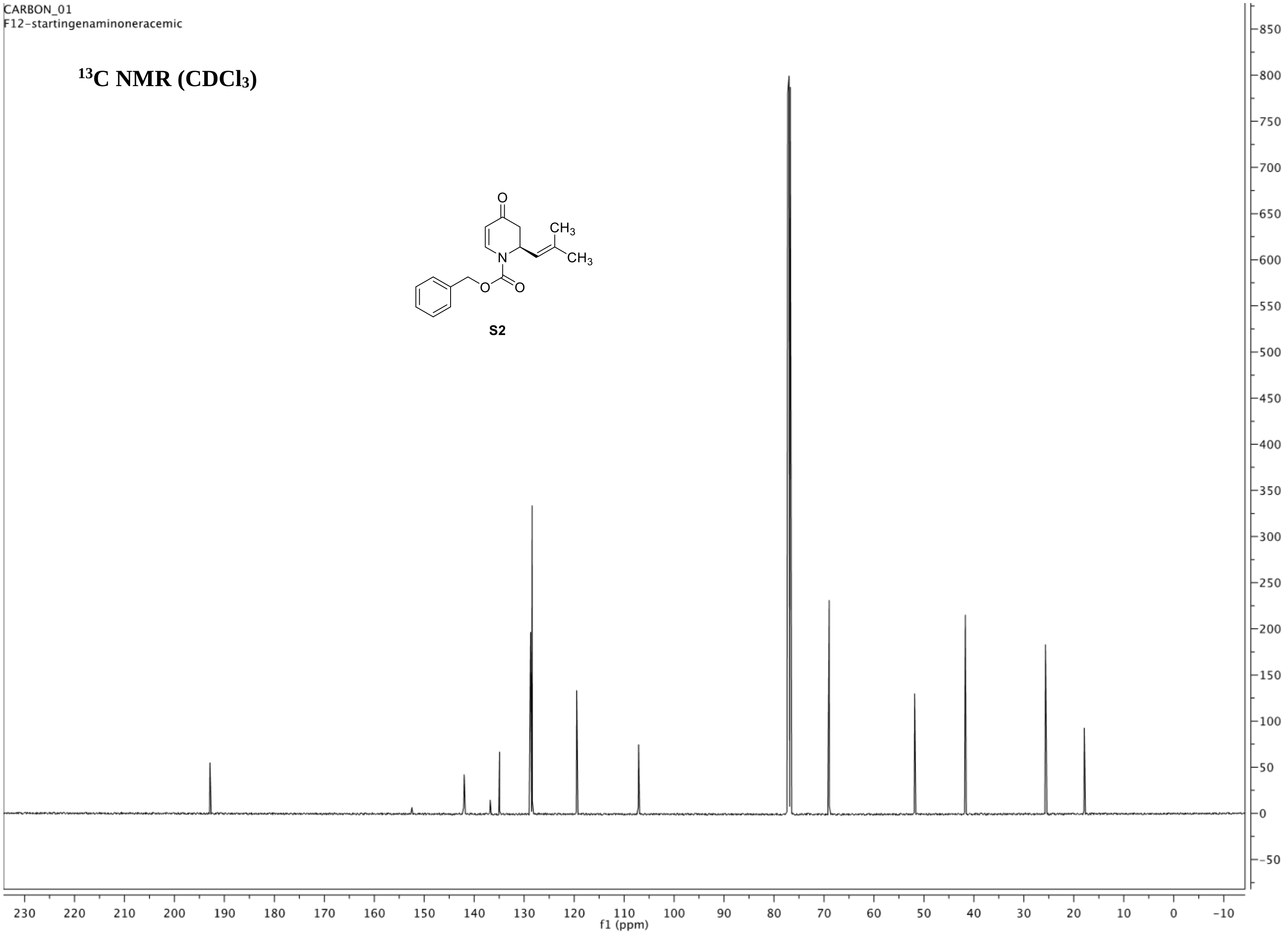
CARBON_01
F12-startingenaminoneracemic
13C NMR (CDCl3)
O
CH3
N
CH3
O
O
S2
f1 (ppm)

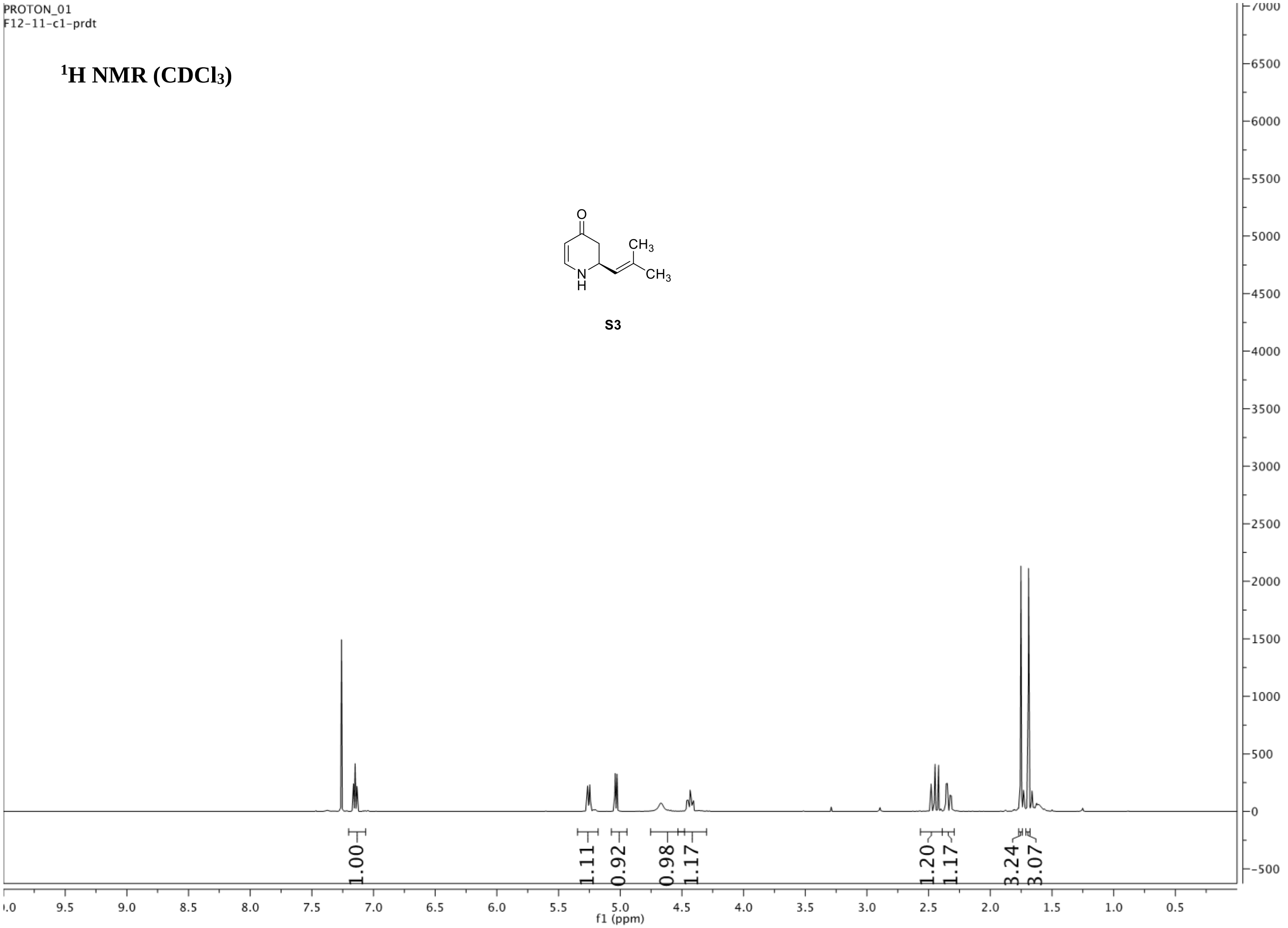
PROTON_01
F12-11-c1-prdt
1H NMR (CDCl3)
S3
1.00
1.11
0.92
0.98
1.17
1.20
1.17
3.24
3.07
f1 (ppm)

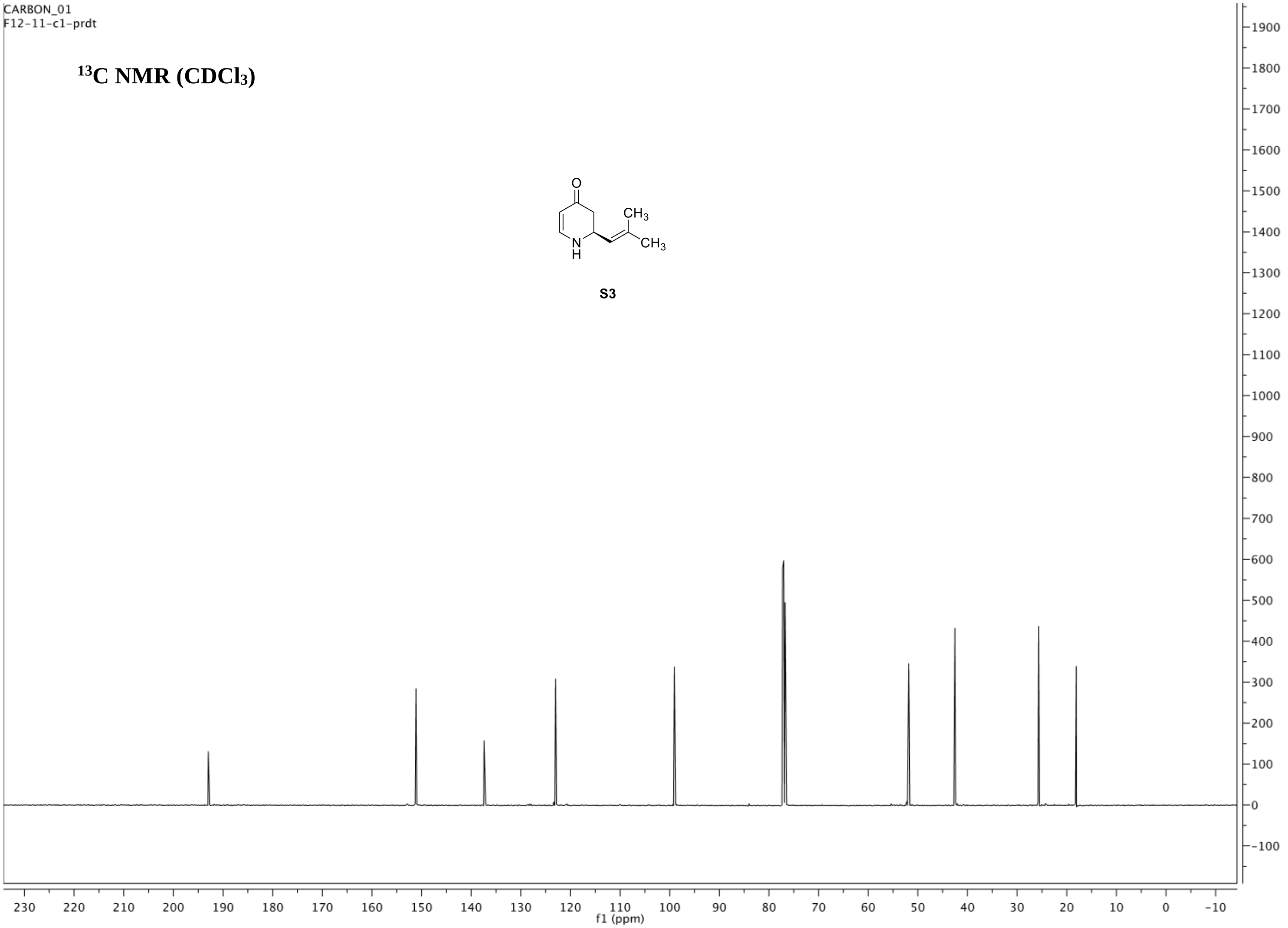
CARBON_01
F12-11-c1-prdt
13C NMR (CDCl3)
S3
f1 (ppm)

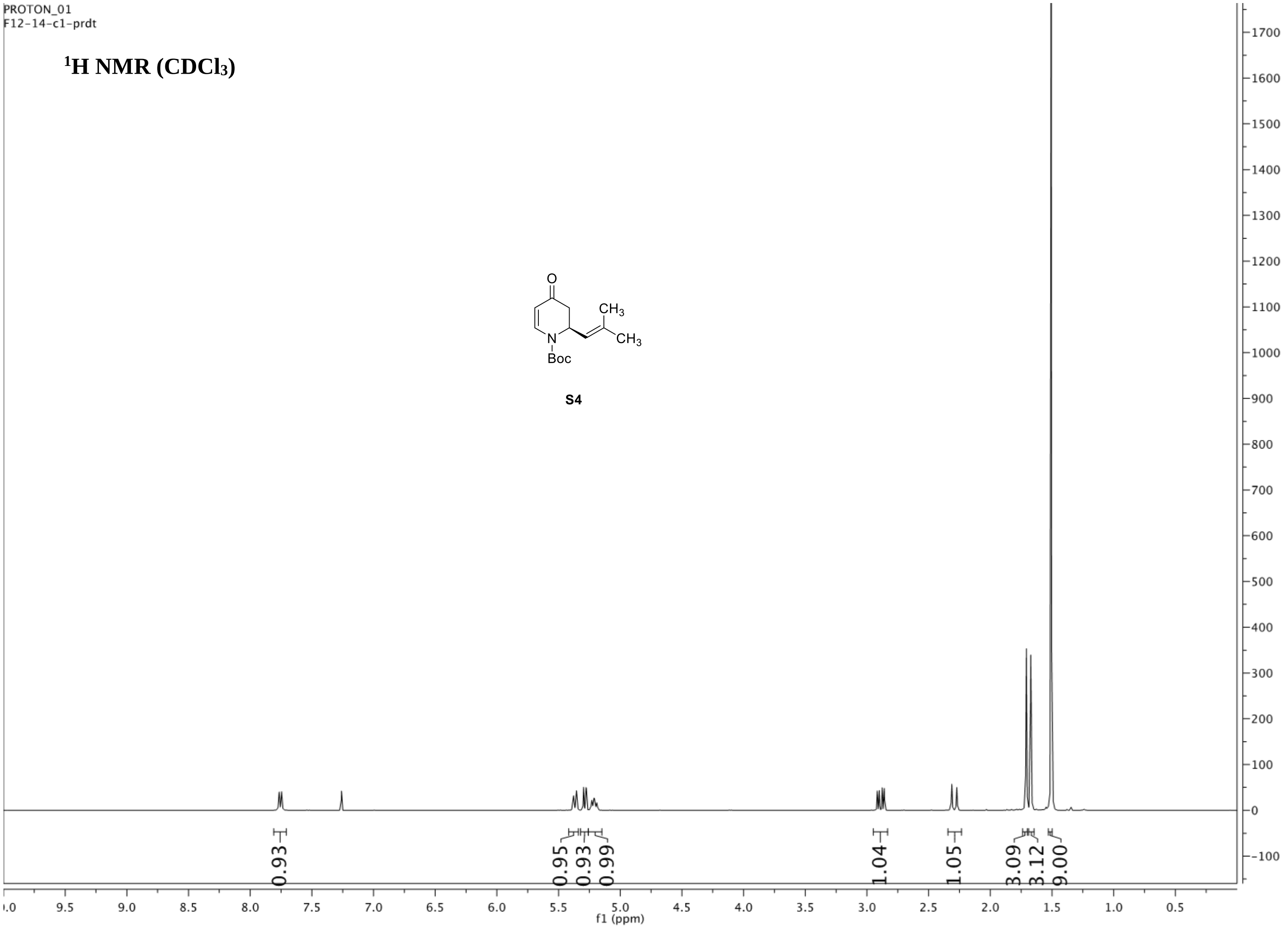
PROTON_01
F12-14-c1-prdt
^{1}H NMR (CDCl$_3$)
O
CH3
N
CH3
Boc
S4
0.93
0.95
0.93
0.99
1.04
1.05
3.09
3.12
9.00
f1 (ppm)

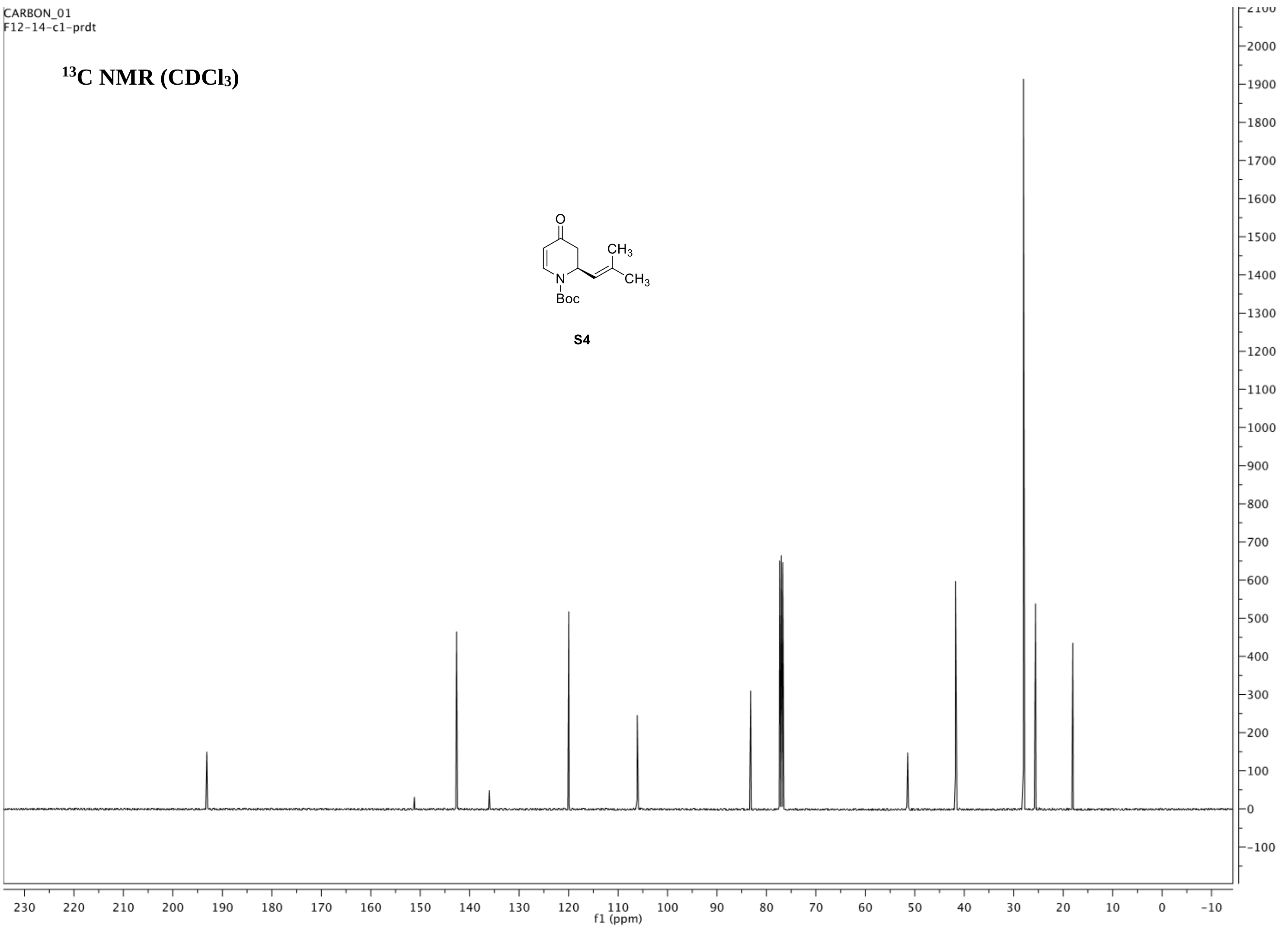
CARBON_01
F12-14-c1-prdt
13C NMR (CDCl3)
O
CH3
N
CH3
Boc
S4
f1 (ppm)

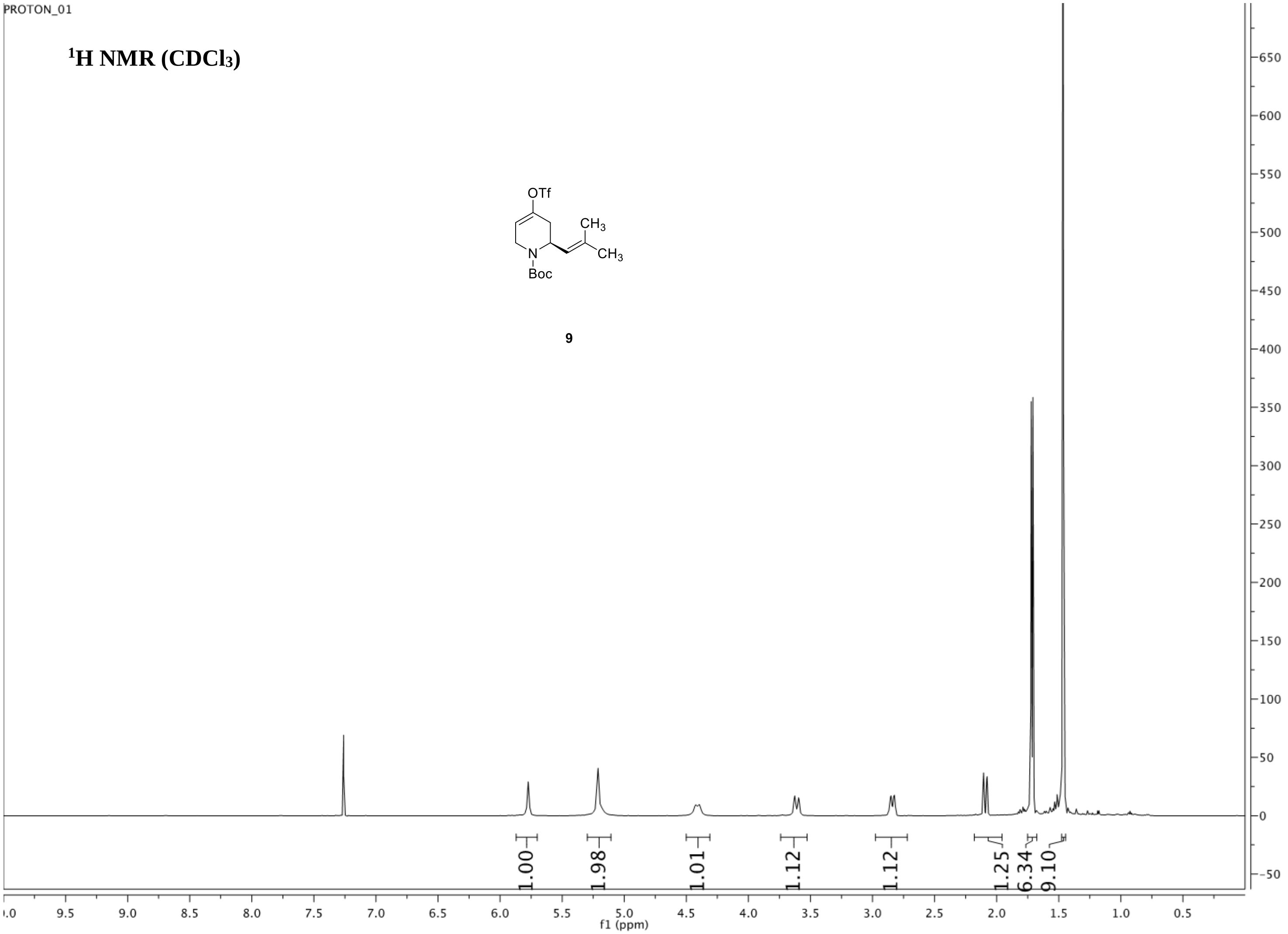
PROTON_01
¹H NMR (CDCl3)
OTf
CH3
CH3
N
Boc
9
1.00
1.98
1.01
1.12
1.12
1.25
6.34
9.10
f1 (ppm)

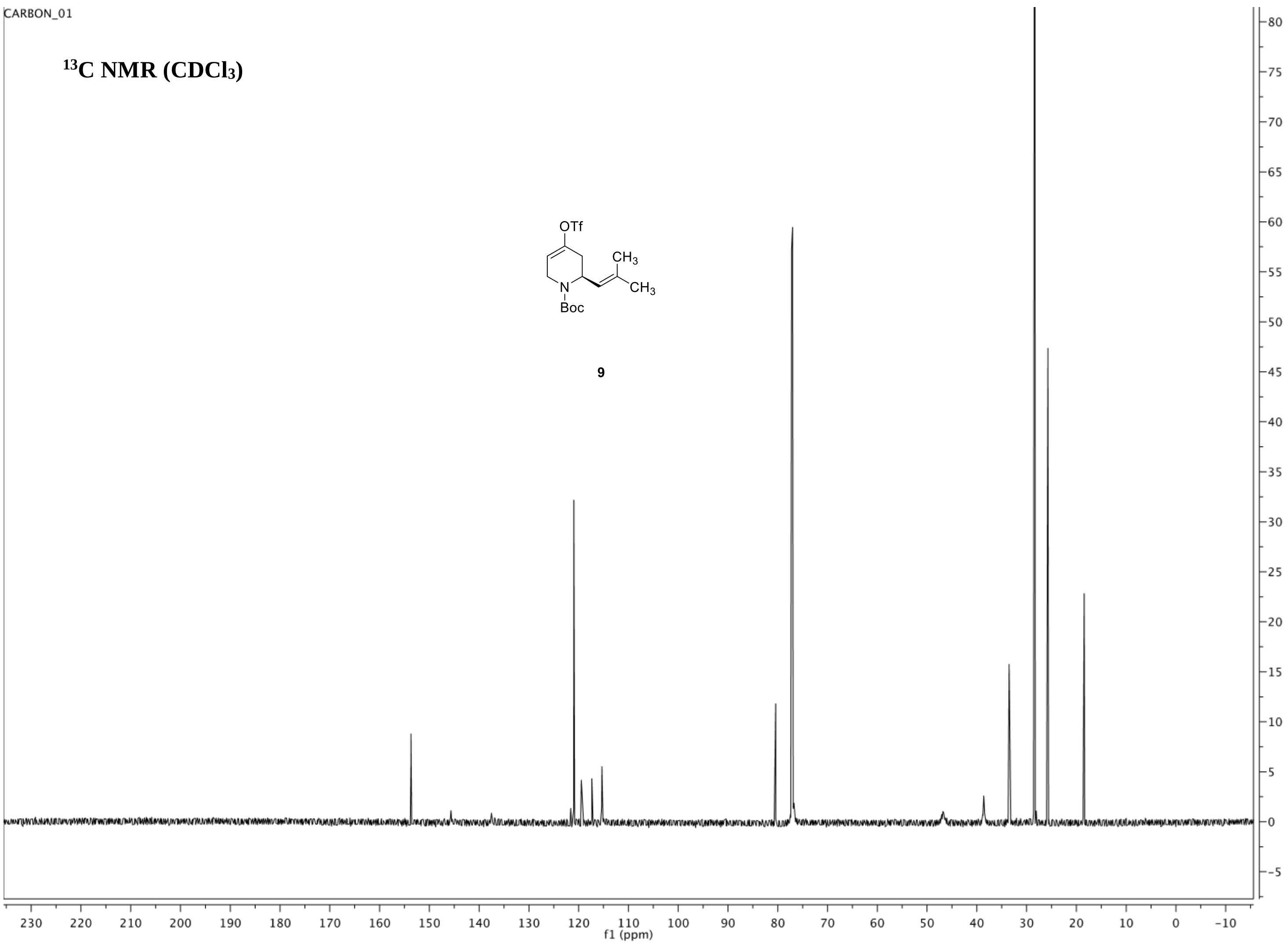
CARBON_01
^{13}C NMR ($CDCl_3$)
OTf
CH_3
N
Boc
9
f1 (ppm)

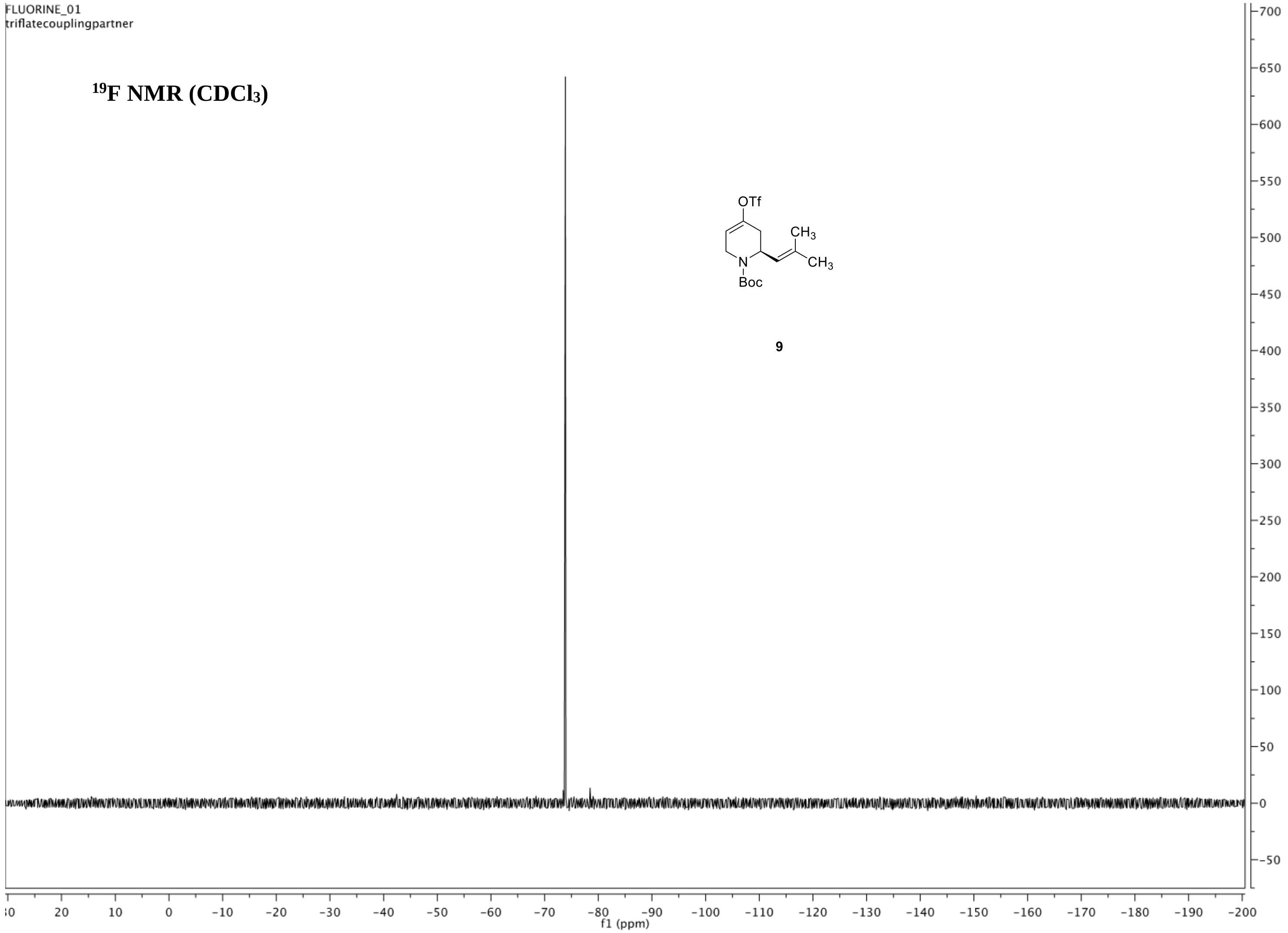
FLUORINE_01
triflatecouplingpartner
19F NMR (CDCl3)
OTf
CH3
CH3
N
Boc
9
f1 (ppm)

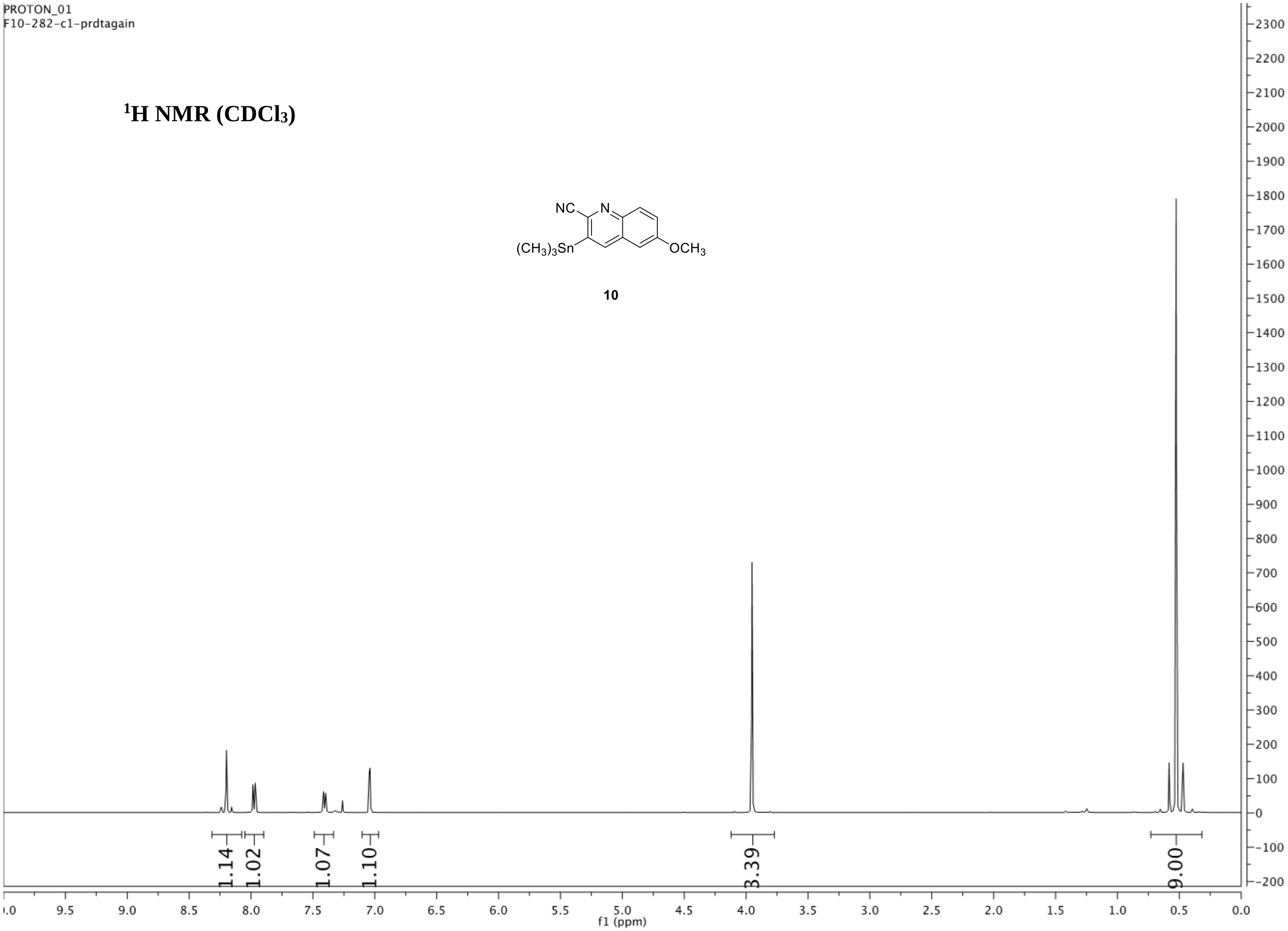
PROTON_01
F10-282-c1-prdtagain
¹H NMR (CDCl3)
NC
N
(CH3)3Sn
OCH3
10
1.14
1.02
1.07
1.10
3.39
9.00
f1 (ppm)

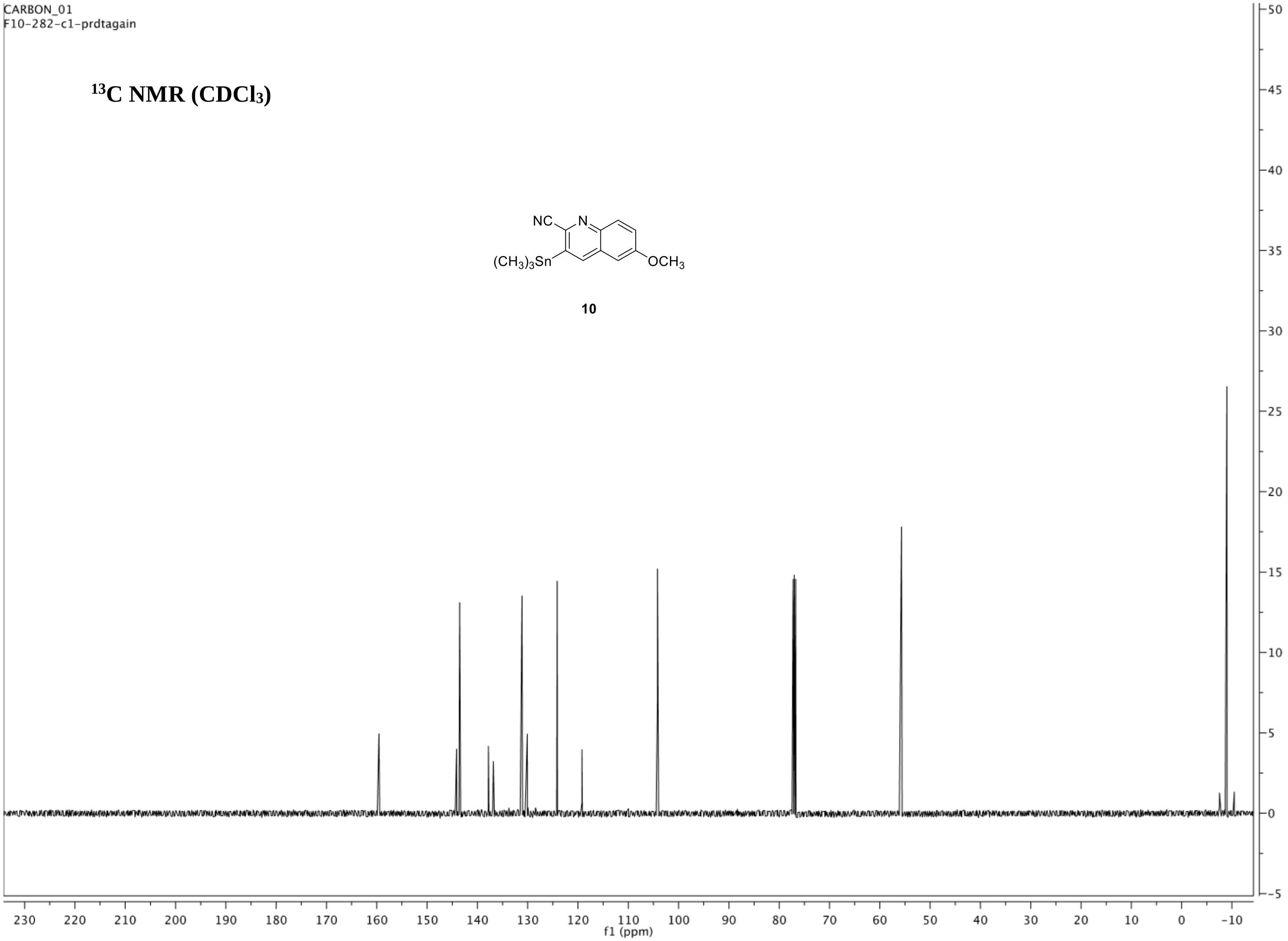
CARBON_01
F10-282-c1-prdtagain
13C NMR (CDCl3)
NC
N
(CH3)3Sn
OCH3
10
f1 (ppm)

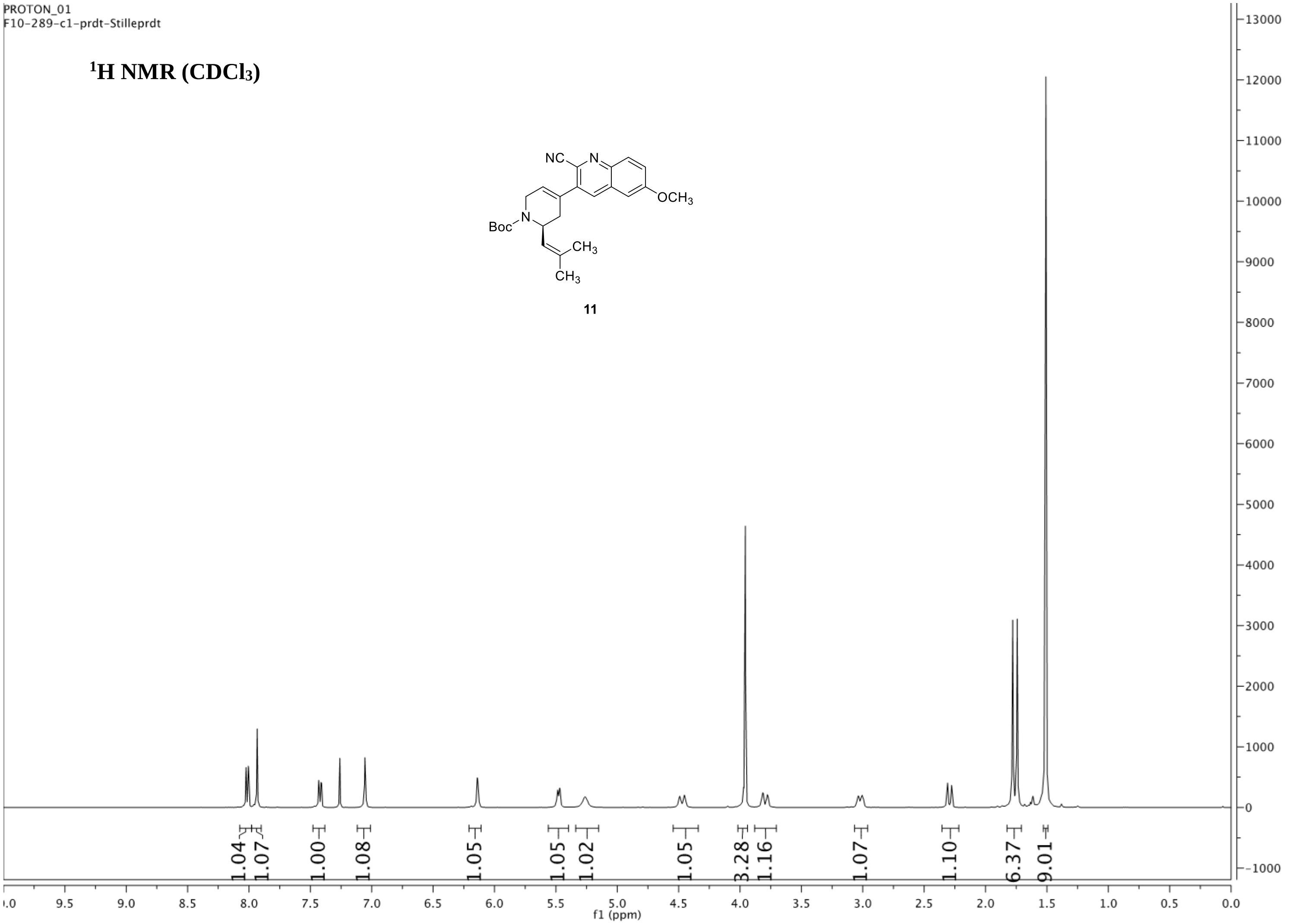
PROTON_01
F10-289-c1-prdt-Stilleprdt
1H NMR (CDCl3)
NC
N
OCH3
Boc
N
CH3
CH3
11
1.04
1.07
1.00
1.08
1.05
1.05
1.02
1.05
3.28
1.16
1.07
1.10
6.37
9.01
f1 (ppm)
9.5
9.0
8.5
8.0
7.5
7.0
6.5
6.0
5.5
5.0
4.5
4.0
3.5
3.0
2.5
2.0
1.5
1.0
0.5
0.0
13000
12000
11000
10000
9000
8000
7000
6000
5000
4000
3000
2000
1000
0
-1000

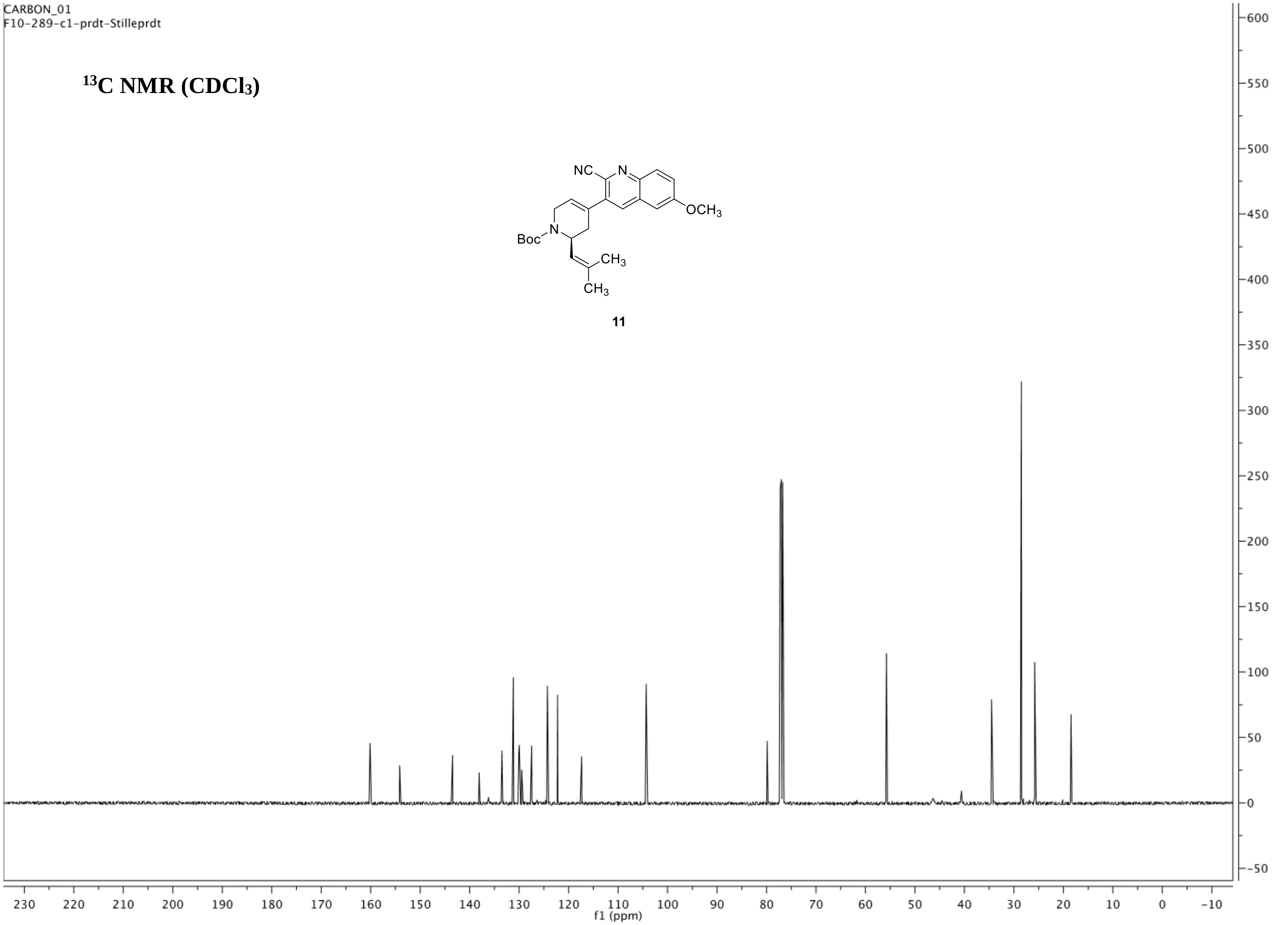
CARBON_01
F10-289-c1-prdt-Stilleprdt
^{13}C NMR ($CDCl_3$)
NC
N
OCH3
Boc
N
CH3
CH3
11
f1 (ppm)

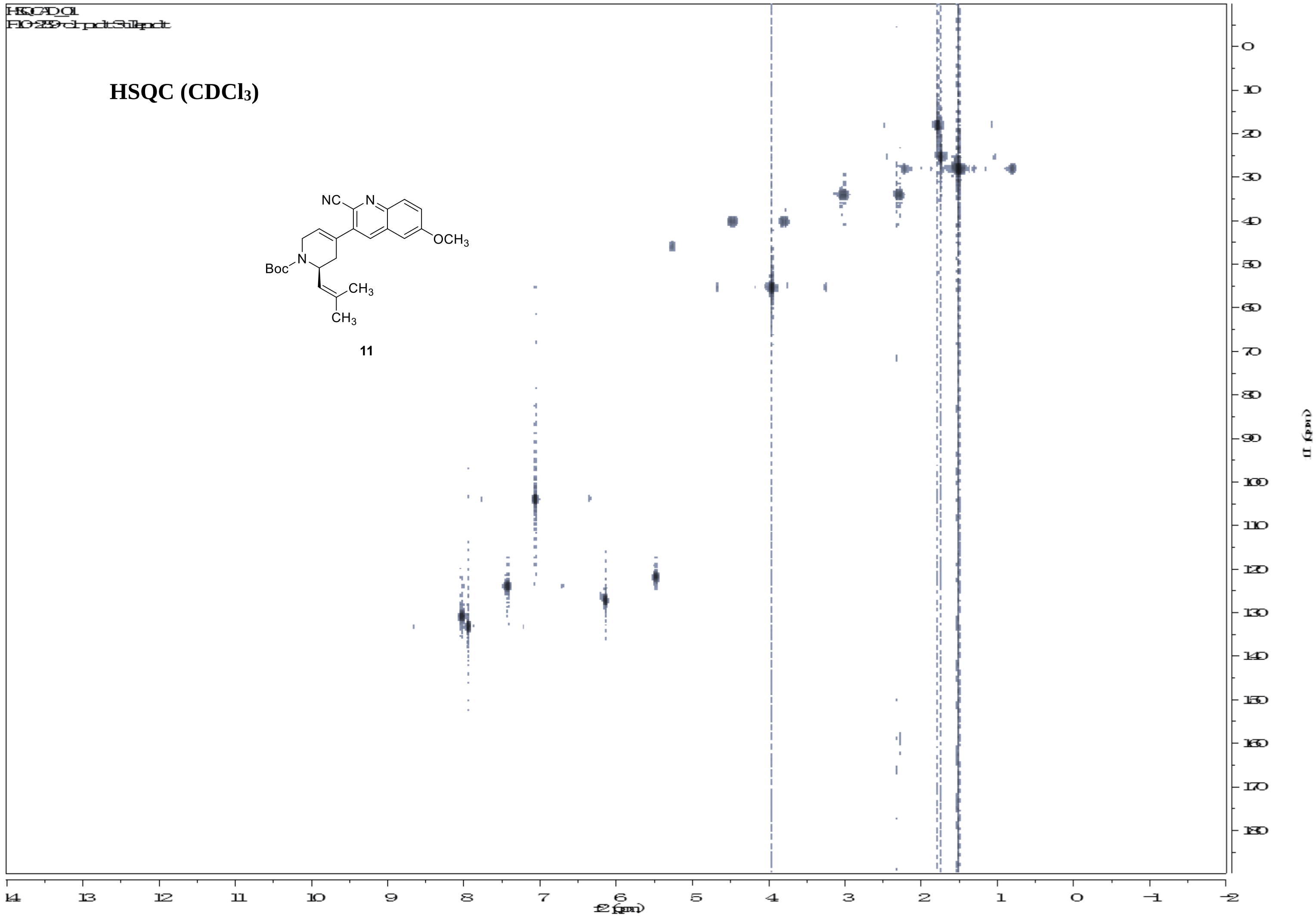
HSQC (CDCl3)
NC
N
OCH3
Boc
N
CH3
CH3
11
f2 (ppm)
f1 (ppm)

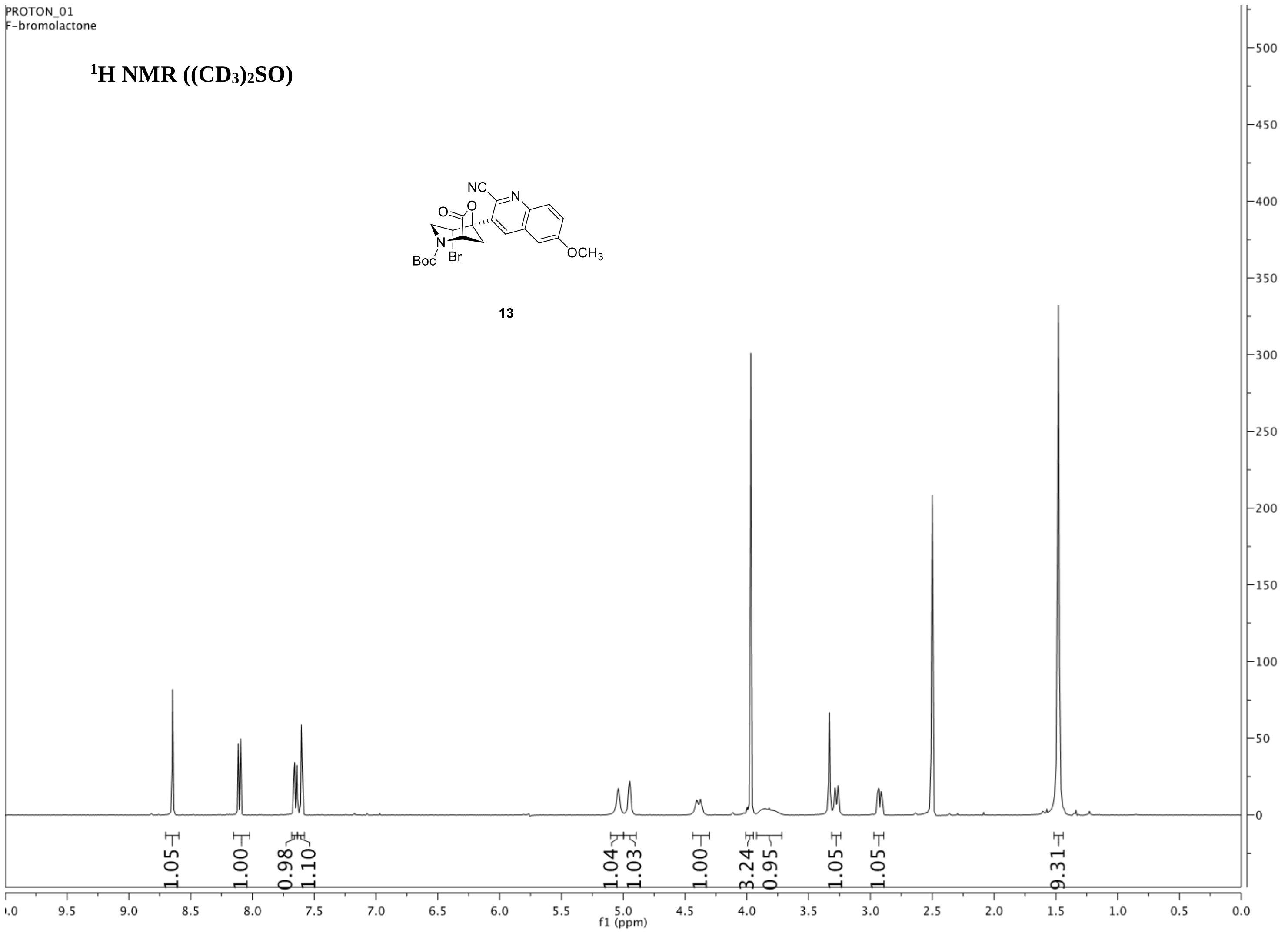
PROTON_01
F-bromolactone
¹H NMR ((CD3)2SO)
13
f1 (ppm)

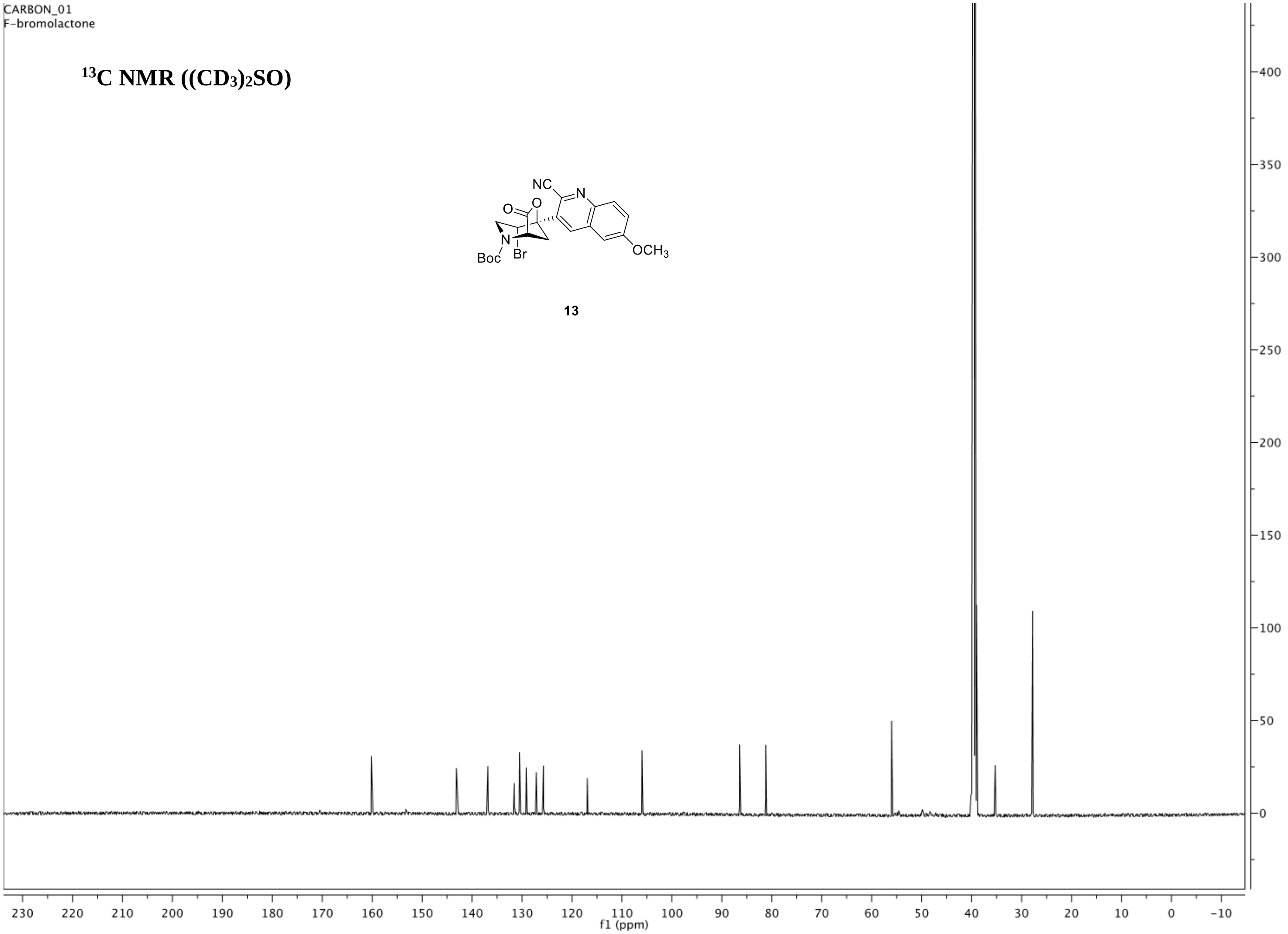
CARBON_01
F-bromolactone
13C NMR ((CD3)2SO)
NC
N
O
O
N
Br
Boc
OCH3
13
f1 (ppm)

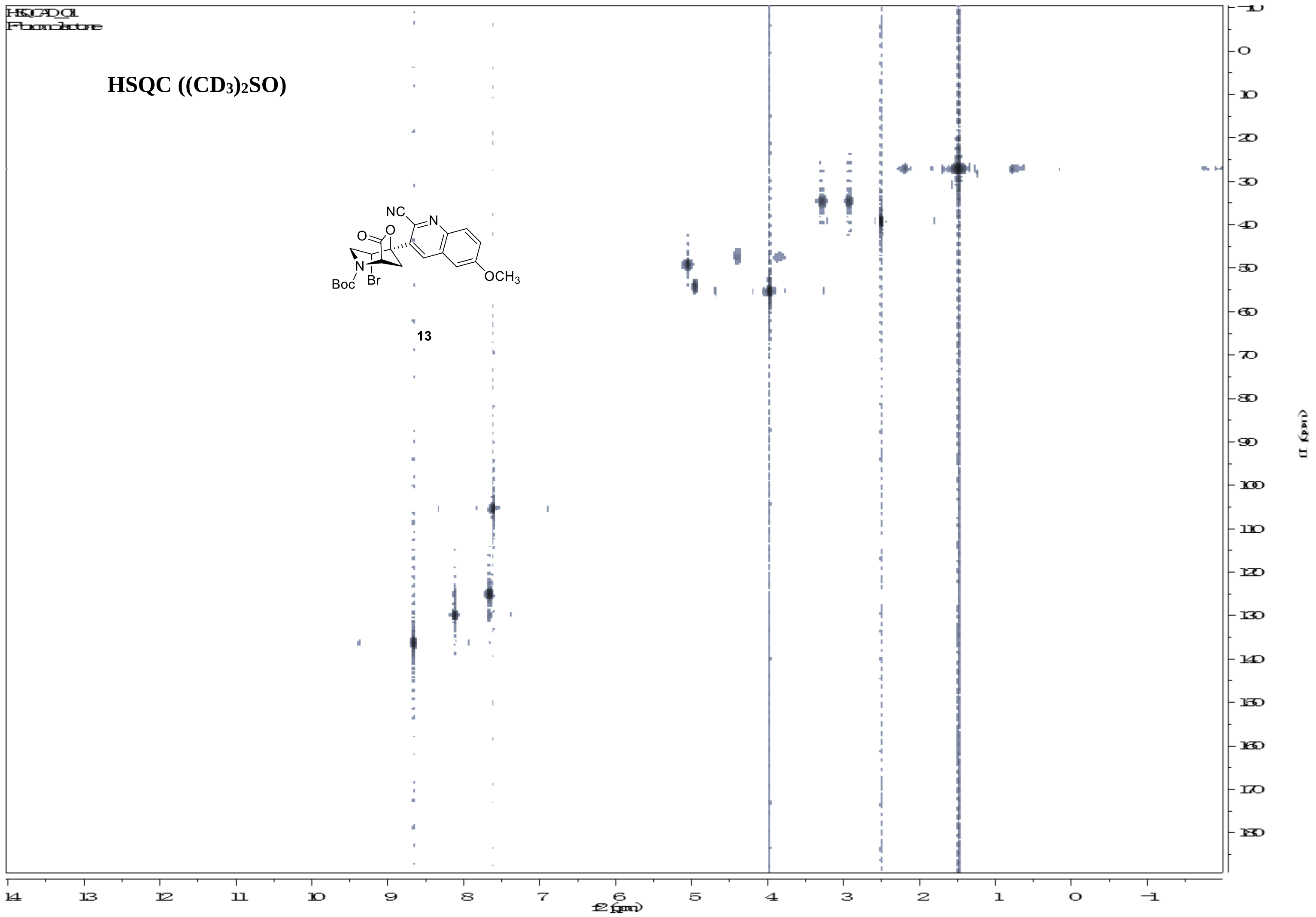
HSQC ((CD3)2SO)
NC
N
O
O
N
Br
Boc
OCH3
13
f2 (ppm)
f1 (ppm)

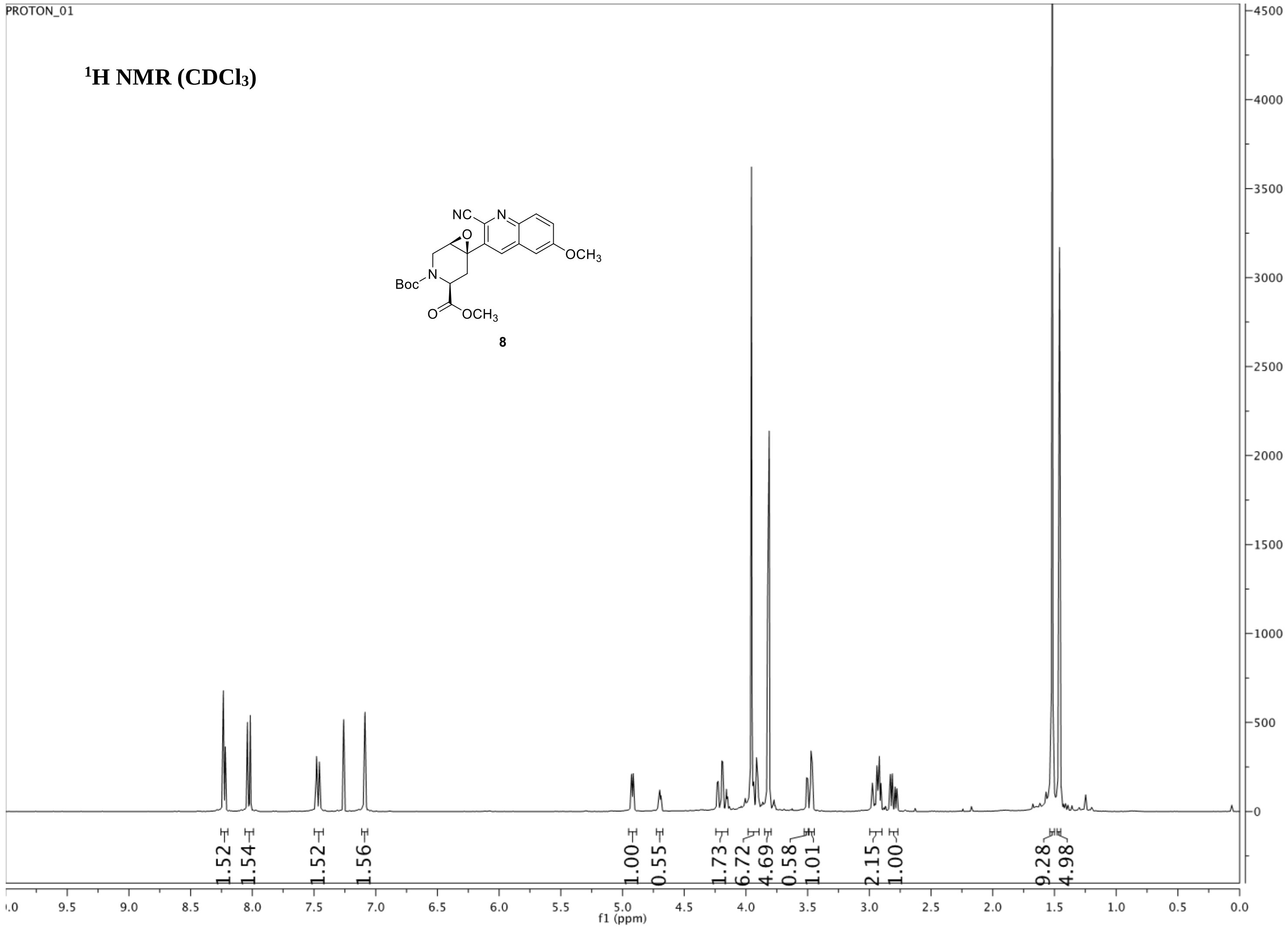
PROTON_01
1H NMR (CDCl3)
NC
N
O
OCH3
Boc
N
O
OCH3
8
1.52
1.54
1.52
1.56
1.00
0.55
1.73
6.72
4.69
0.58
1.01
2.15
1.00
9.28
4.98
f1 (ppm)

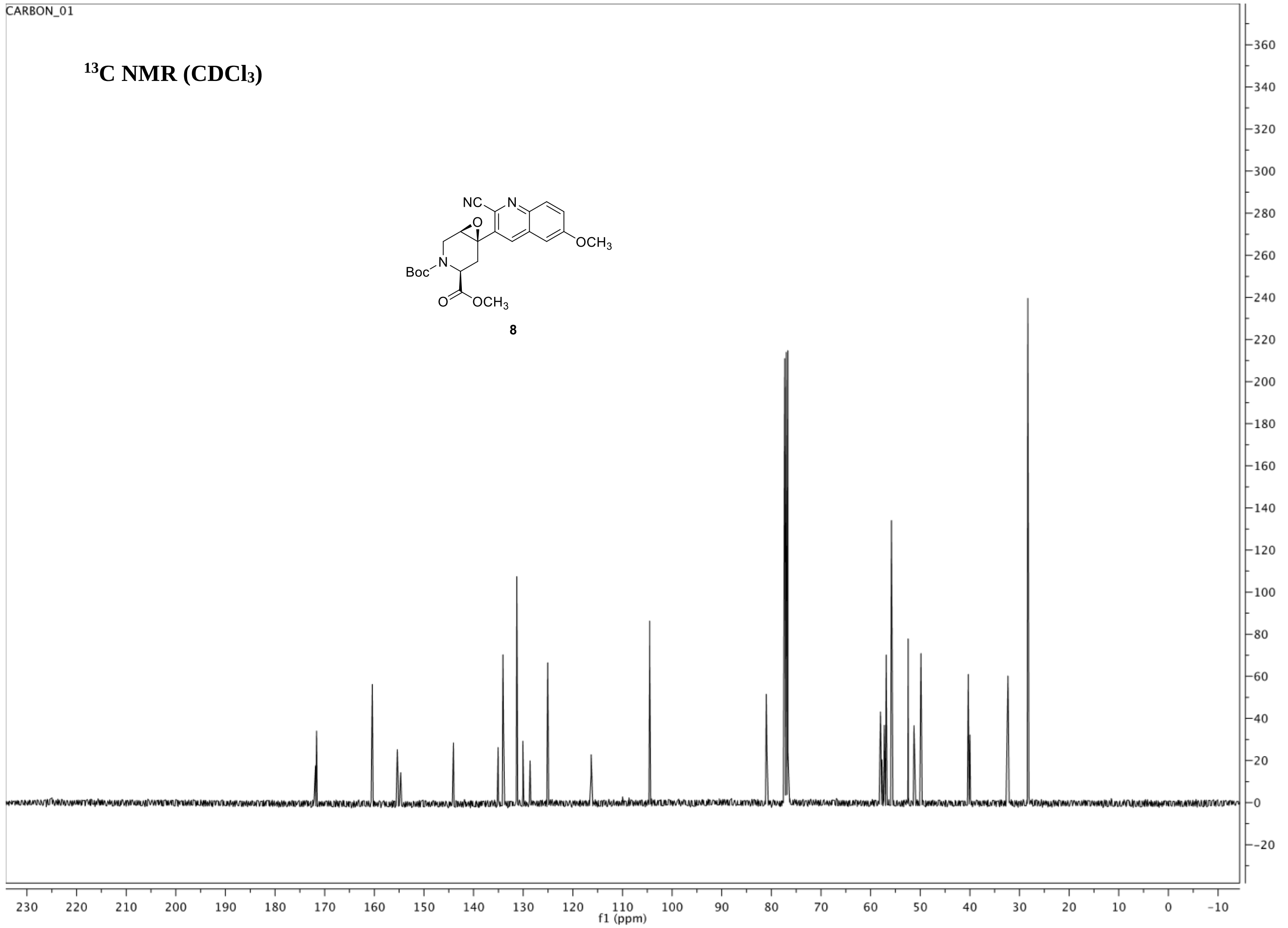
CARBON_01
^{13}C NMR (CDCl$_3$)
NC
N
O
OCH3
Boc
N
O
OCH3
8
f1 (ppm)

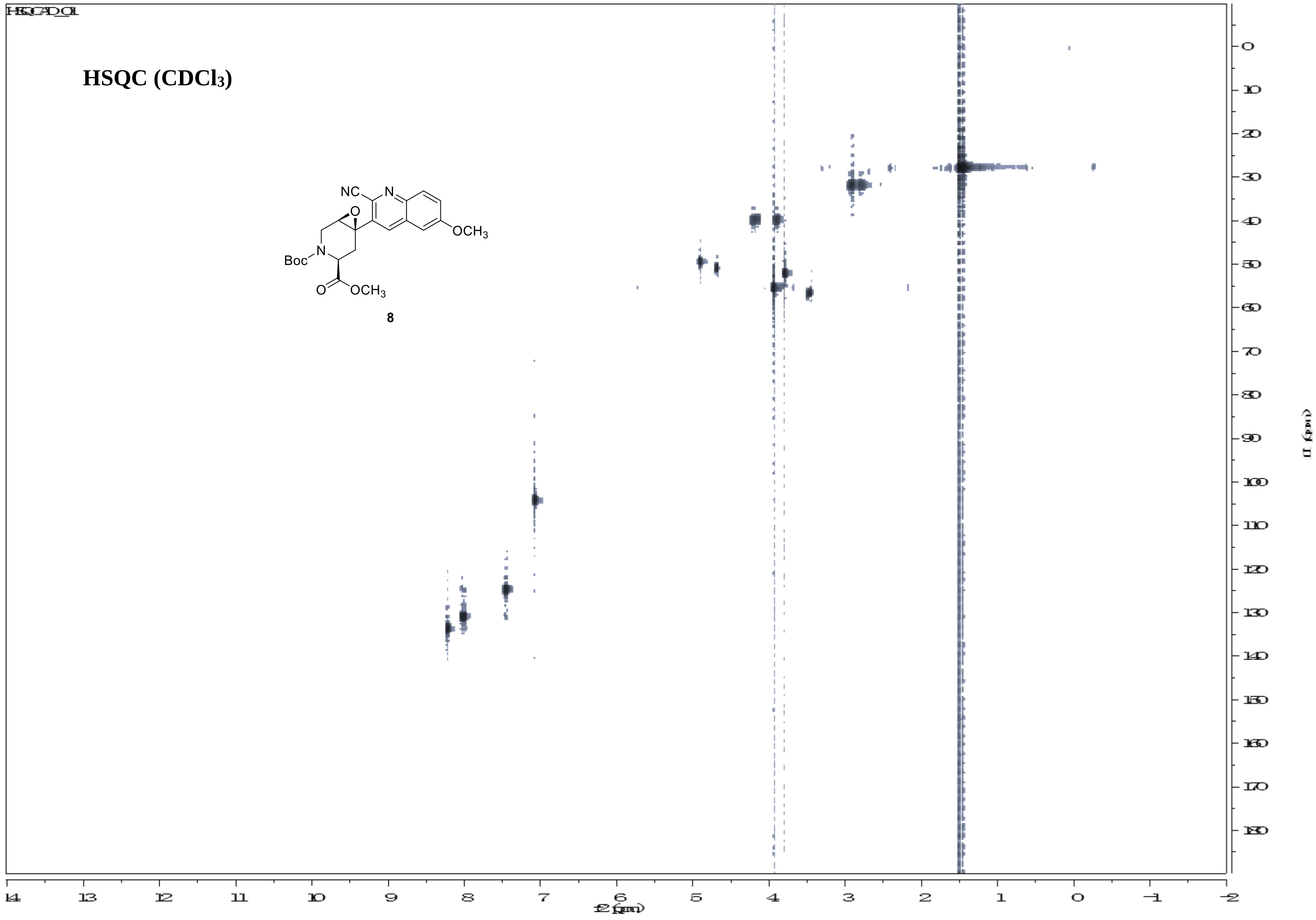
HSQC (CDCl3)
NC
N
O
OCH3
Boc
N
O
OCH3
8
f2 (ppm)
f1 (ppm)

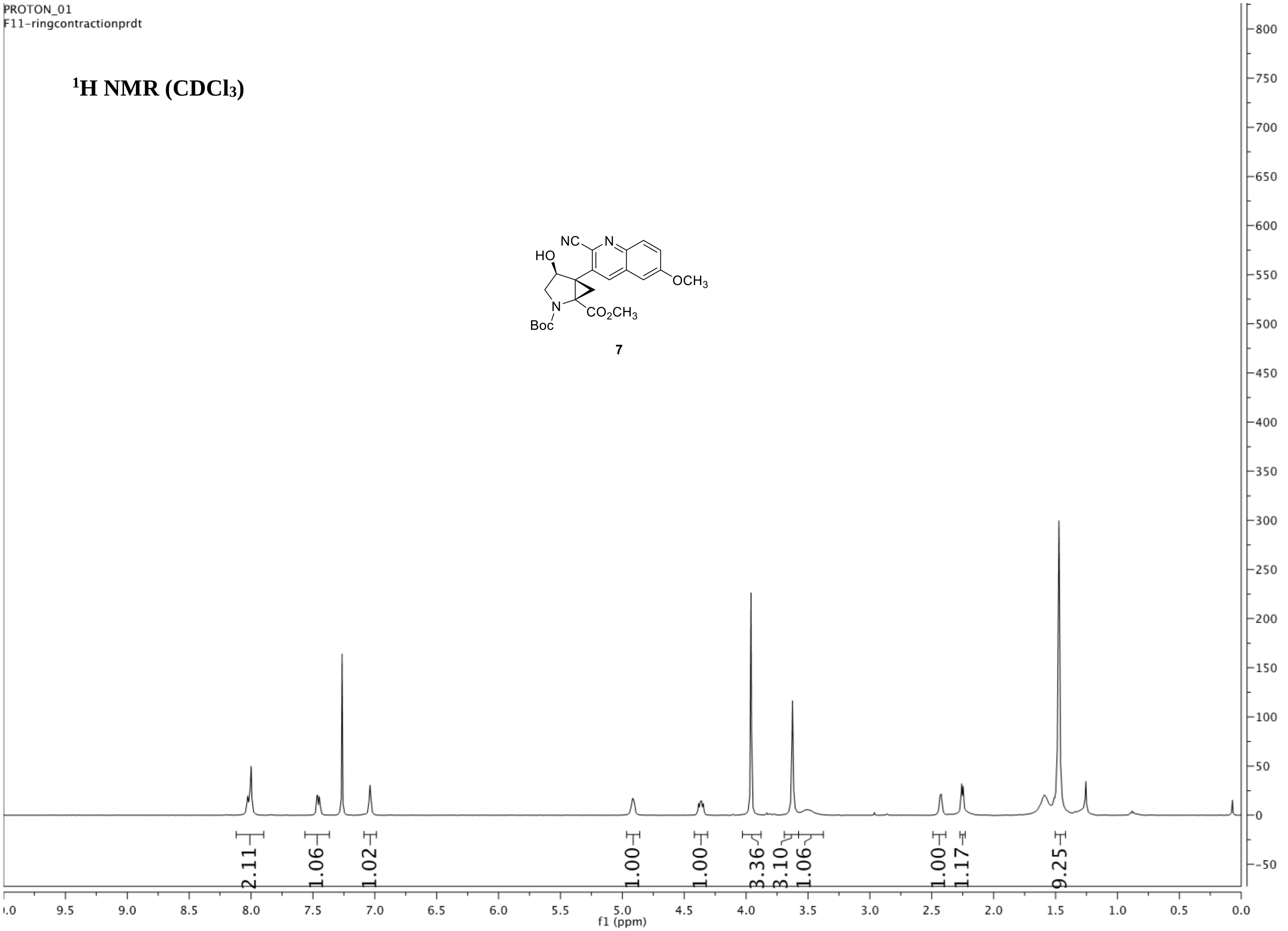
PROTON_01
F11-ringcontractionprdt
¹H NMR (CDCl₃)
7
f1 (ppm)

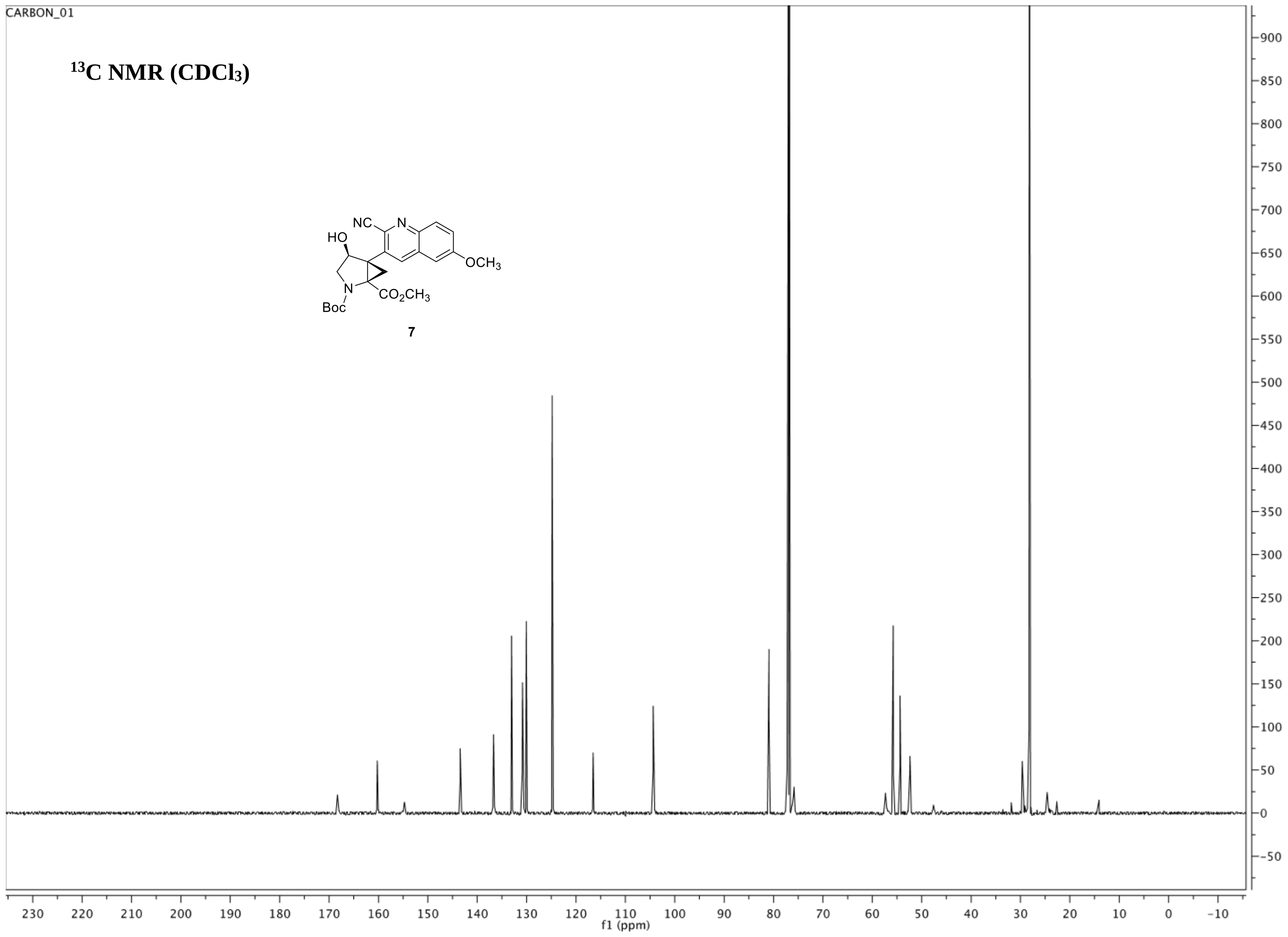
CARBON_01
13C NMR (CDCl3)
HO
NC
N
OCH3
N
Boc
CO2CH3
7
f1 (ppm)

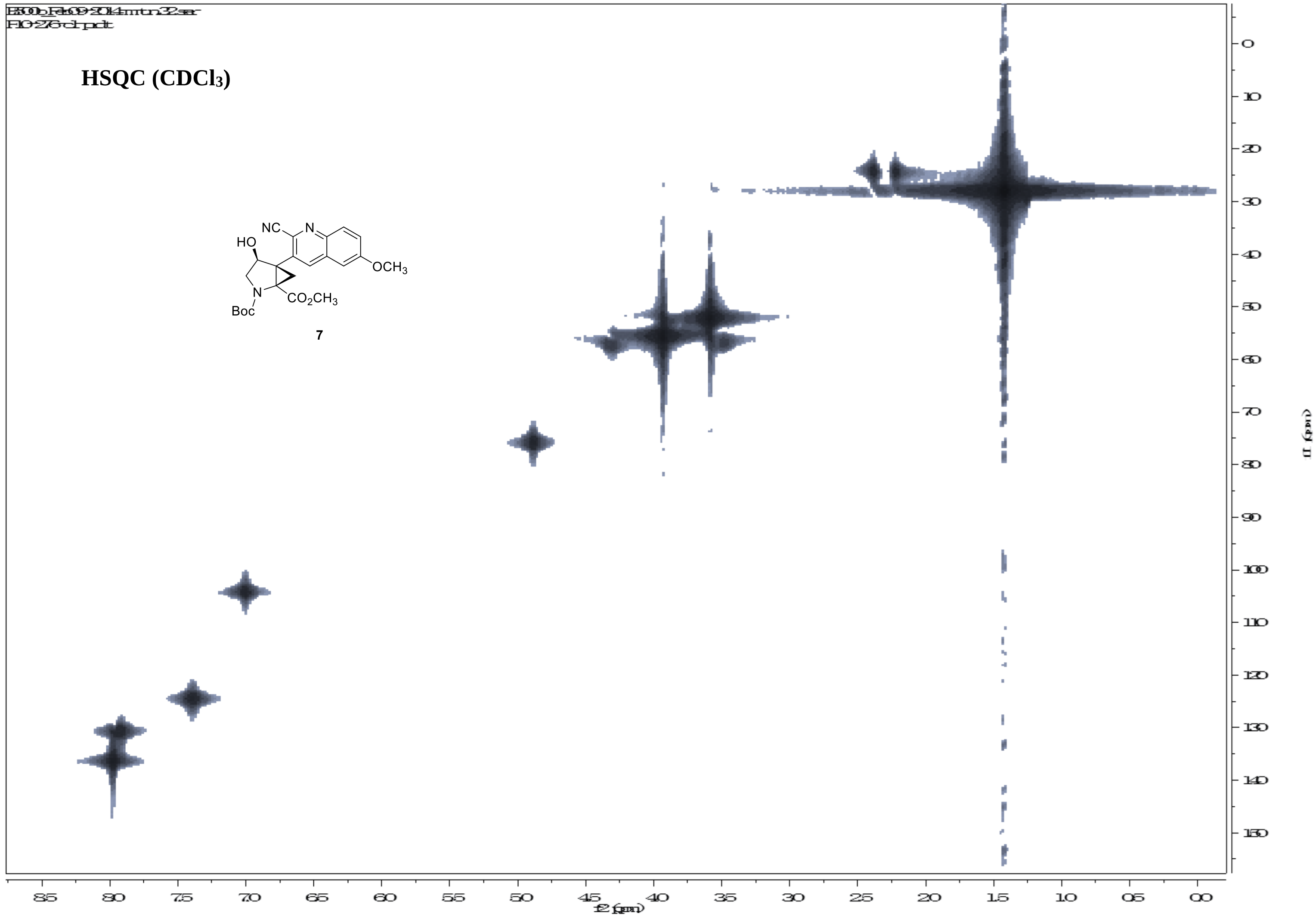
HSQC (CDCl3)
NC
N
HO
OCH3
N
CO2CH3
Boc
7
f2 (ppm)
f1 (ppm)

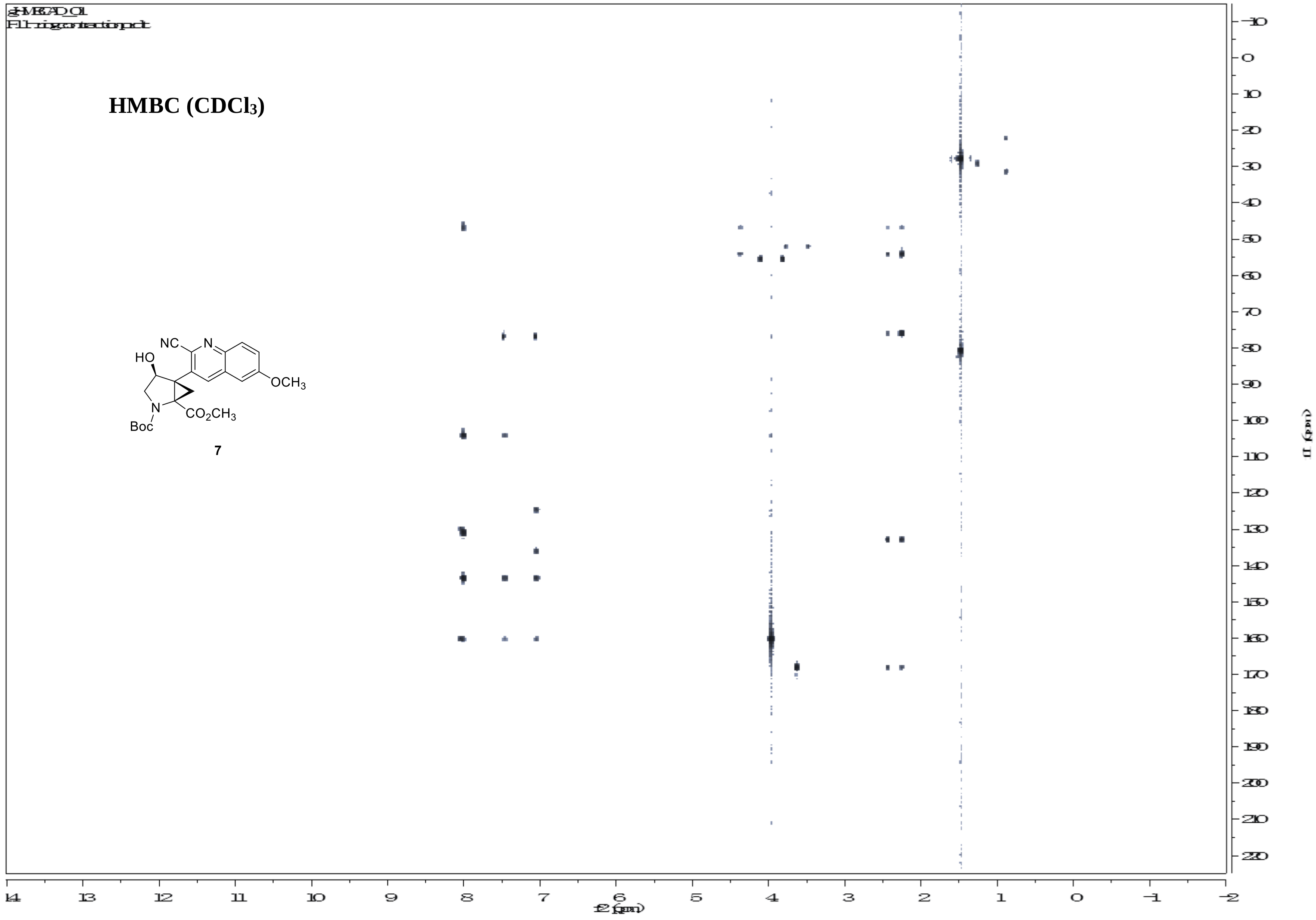
HMBC (CDCl3)
HO
NC
N
OCH3
CO2CH3
Boc
7
f2 (ppm)
f1 (ppm)

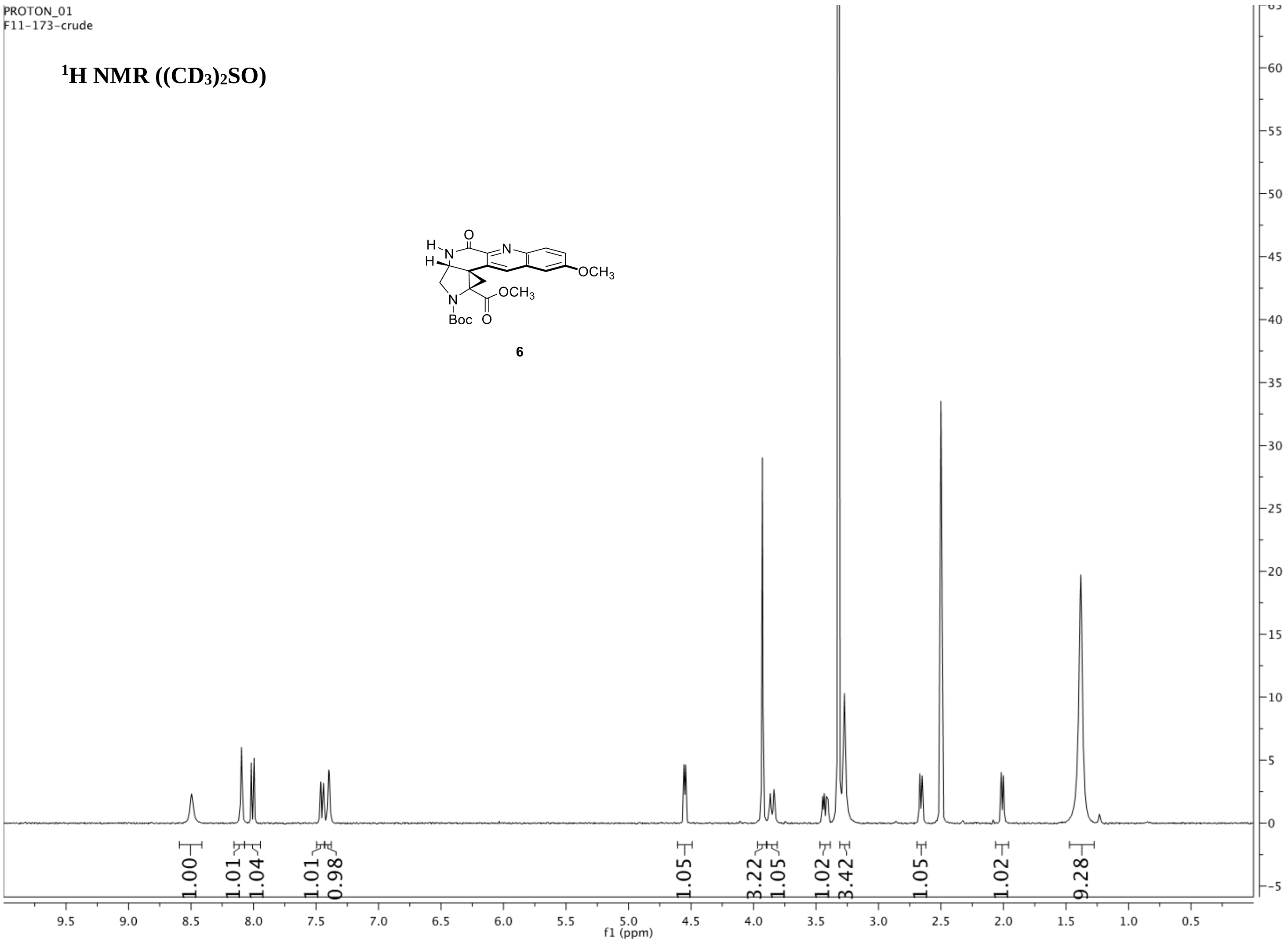
PROTON_01
F11-173-crude
1H NMR ((CD3)2SO)
OCH3
OCH3
Boc
6
1.00
1.01
1.04
1.01
0.98
1.05
3.22
1.05
1.02
3.42
1.05
1.02
9.28
f1 (ppm)

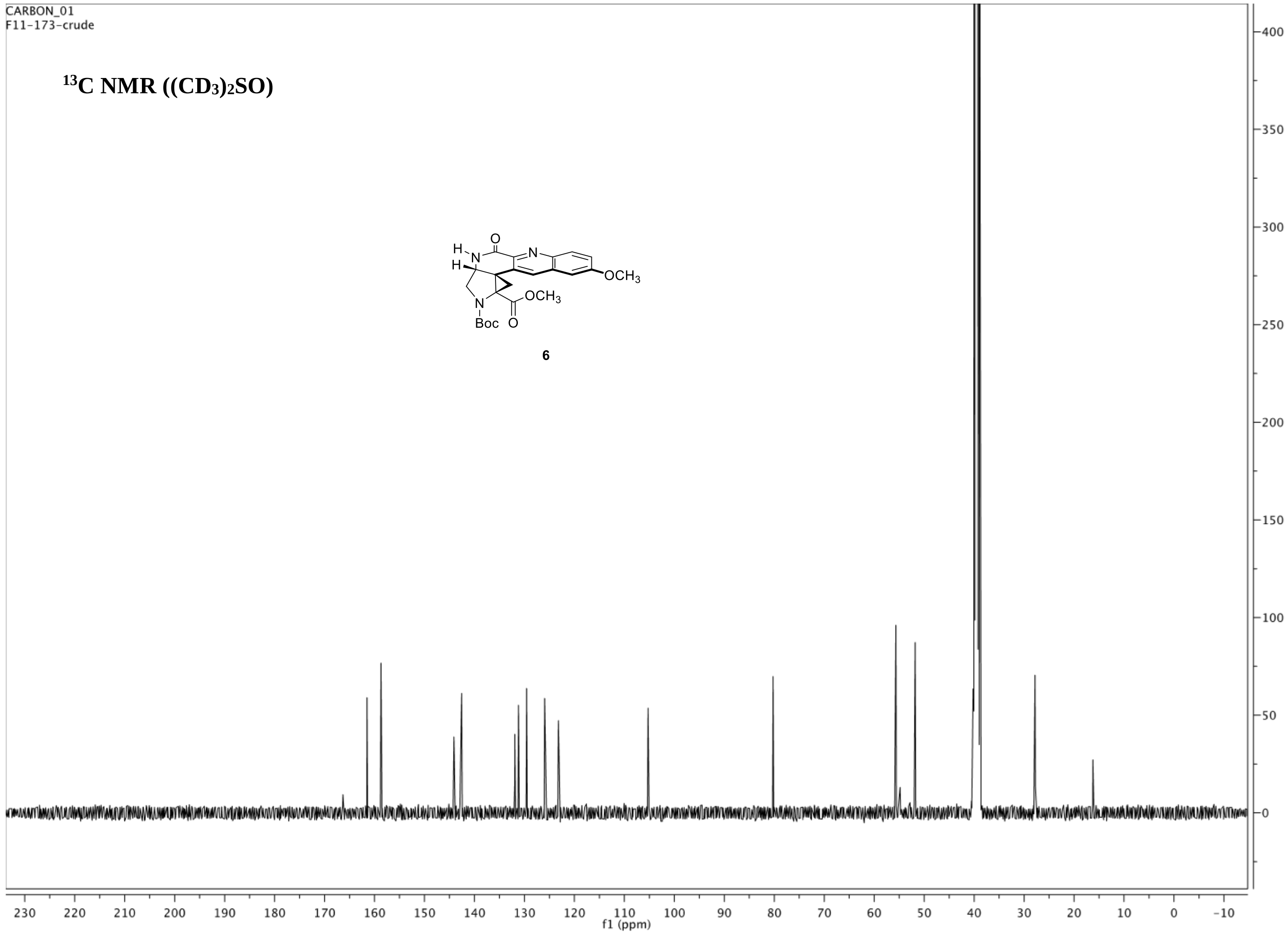
CARBON_01
F11-173-crude
13C NMR ((CD3)2SO)
OCH3
OCH3
Boc
6
f1 (ppm)

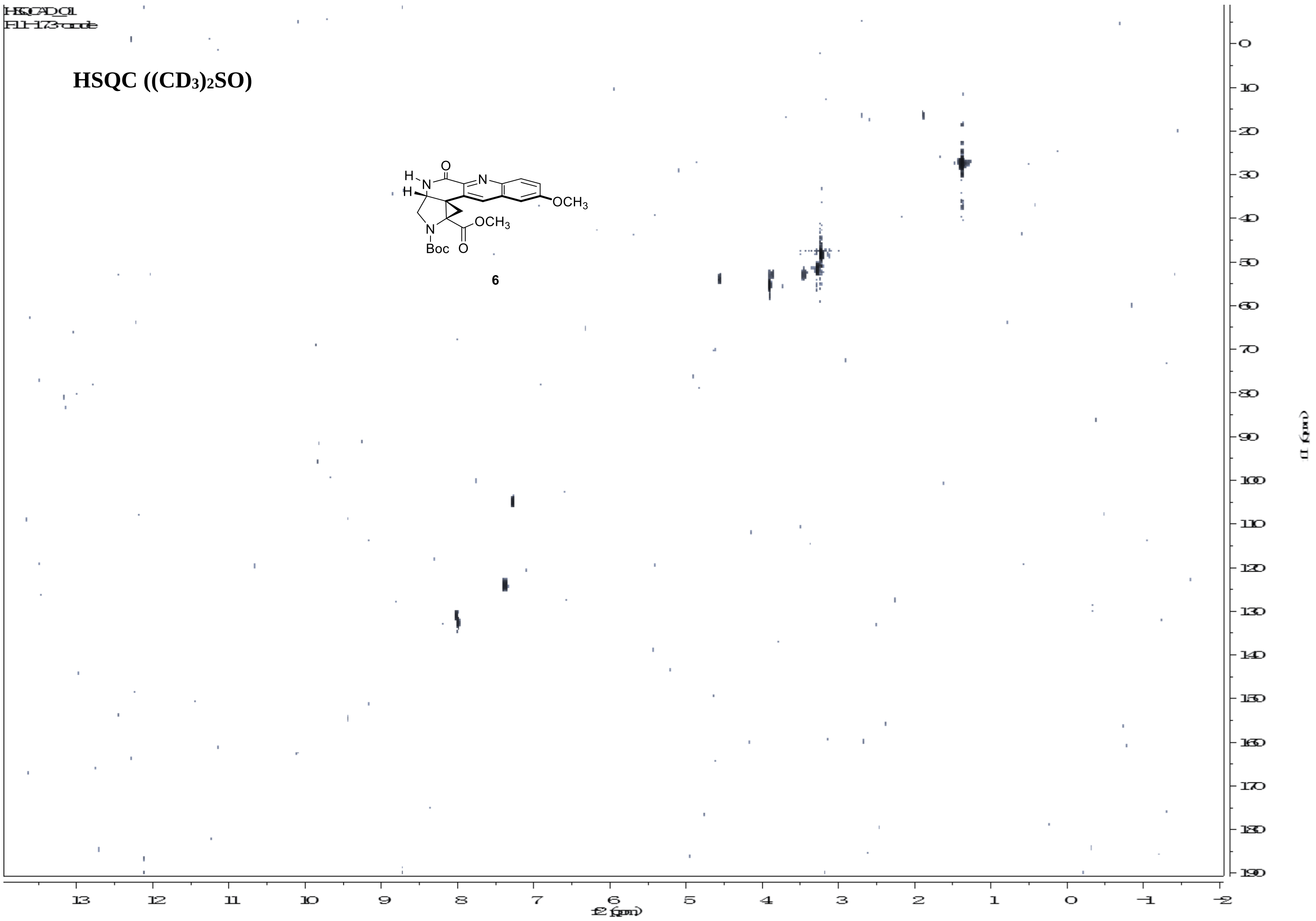
HSQCADQ1
F11-173-crude
HSQC ((CD3)2SO)
OCH3
OCH3
Boc
6
f2 (ppm)
f1 (ppm)

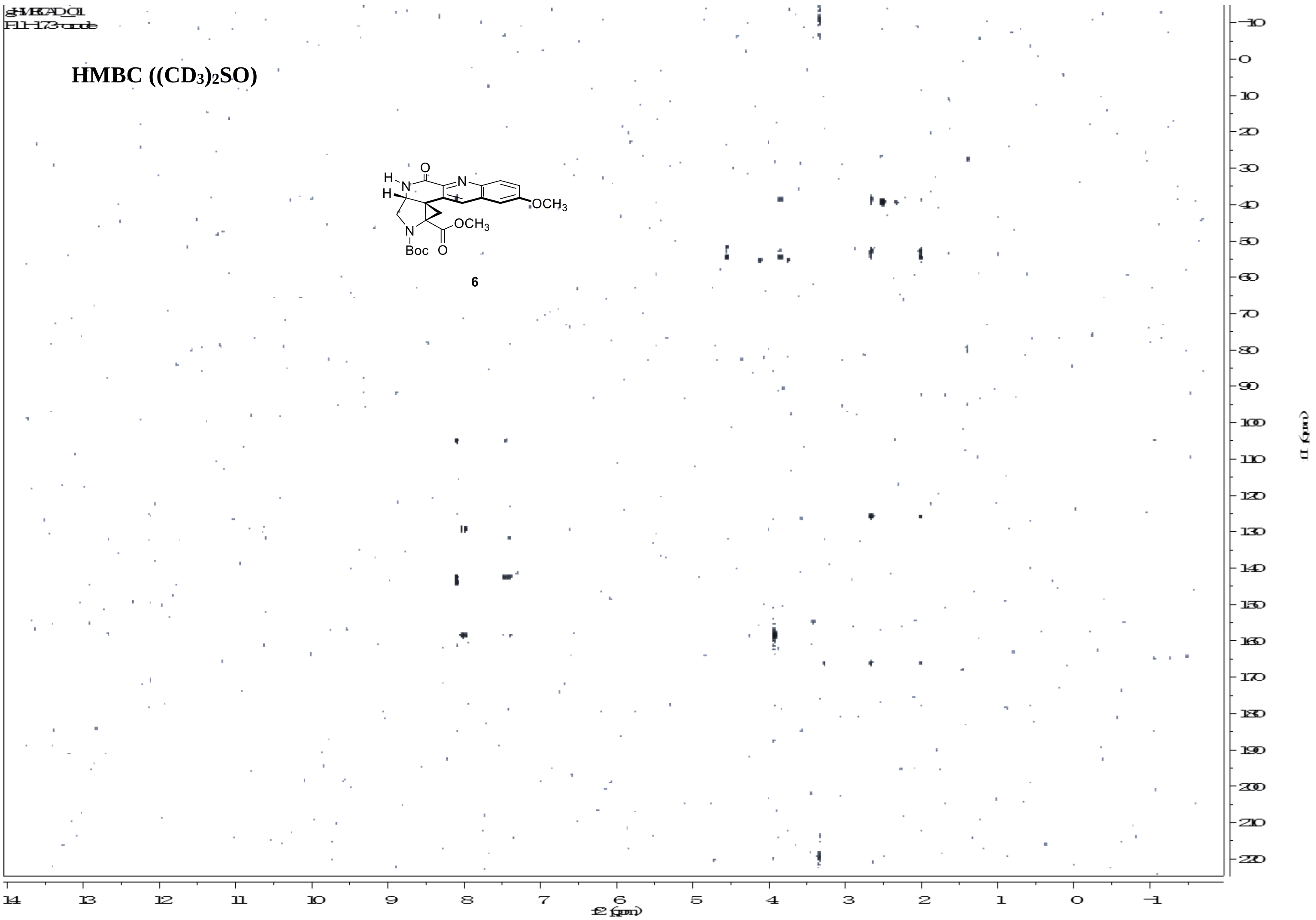
HMBC ((CD3)2SO)
OCH3
OCH3
Boc
6
f2 (ppm)
f1 (ppm)

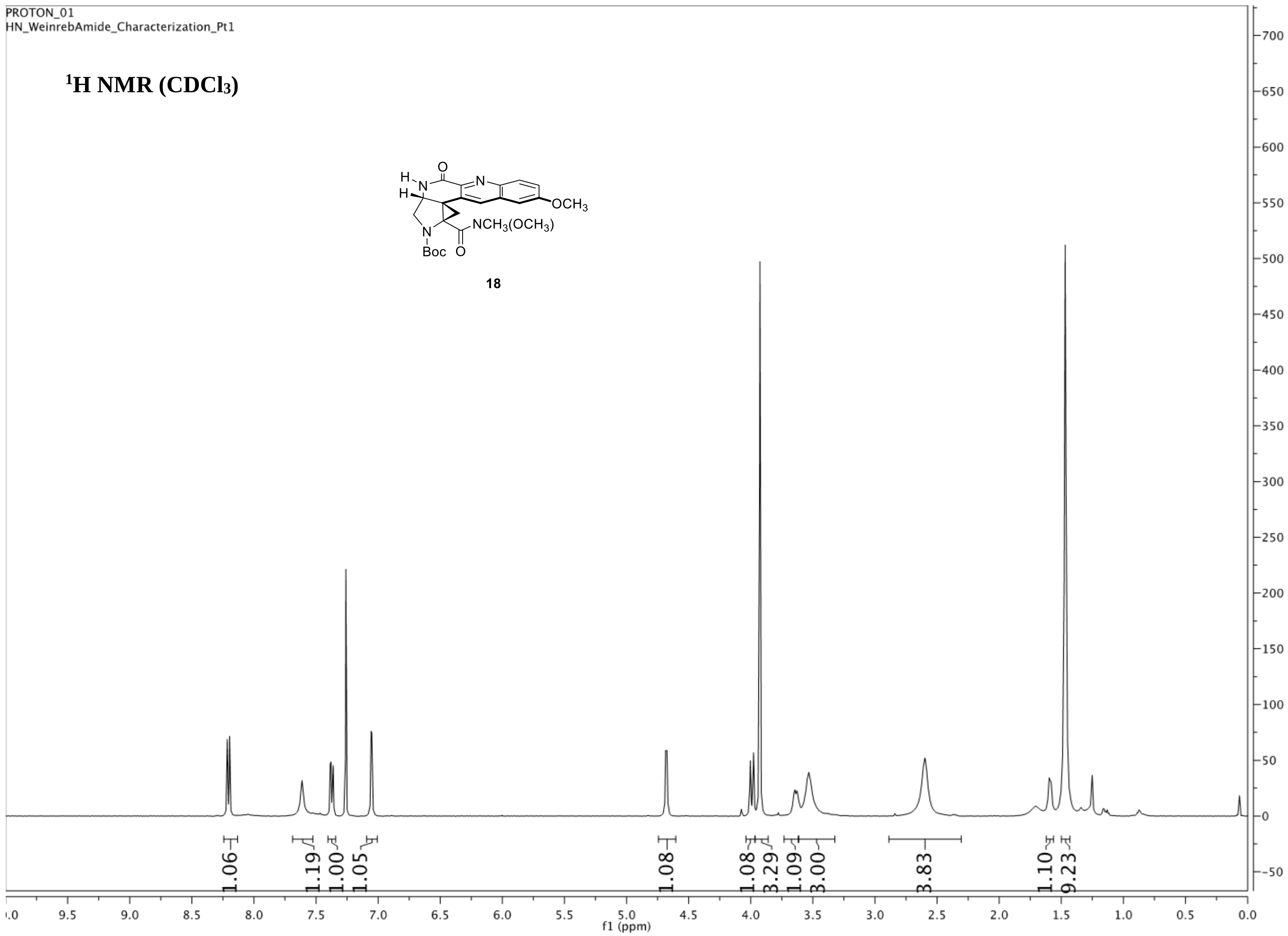
PROTON_01
HN_WeinrebAmide_Characterization_Pt1
1H NMR (CDCl3)
H
N
O
N
H
OCH3
NCH3(OCH3)
N
Boc
O
18
1.06
1.19
1.00
1.05
1.08
1.08
3.29
1.09
3.00
3.83
1.10
9.23
f1 (ppm)

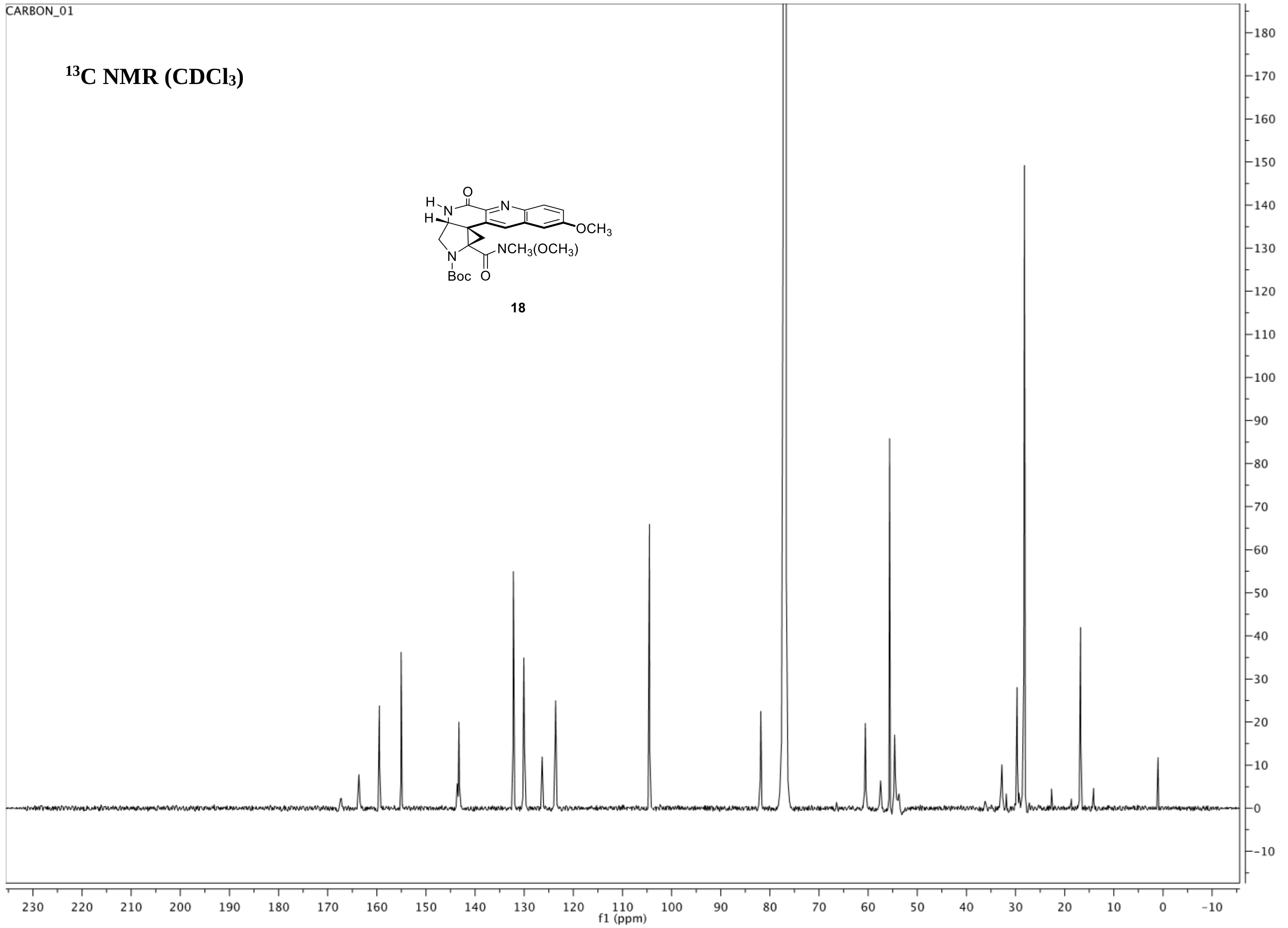
CARBON_01
^{13}C NMR ($CDCl_3$)
18
f1 (ppm)

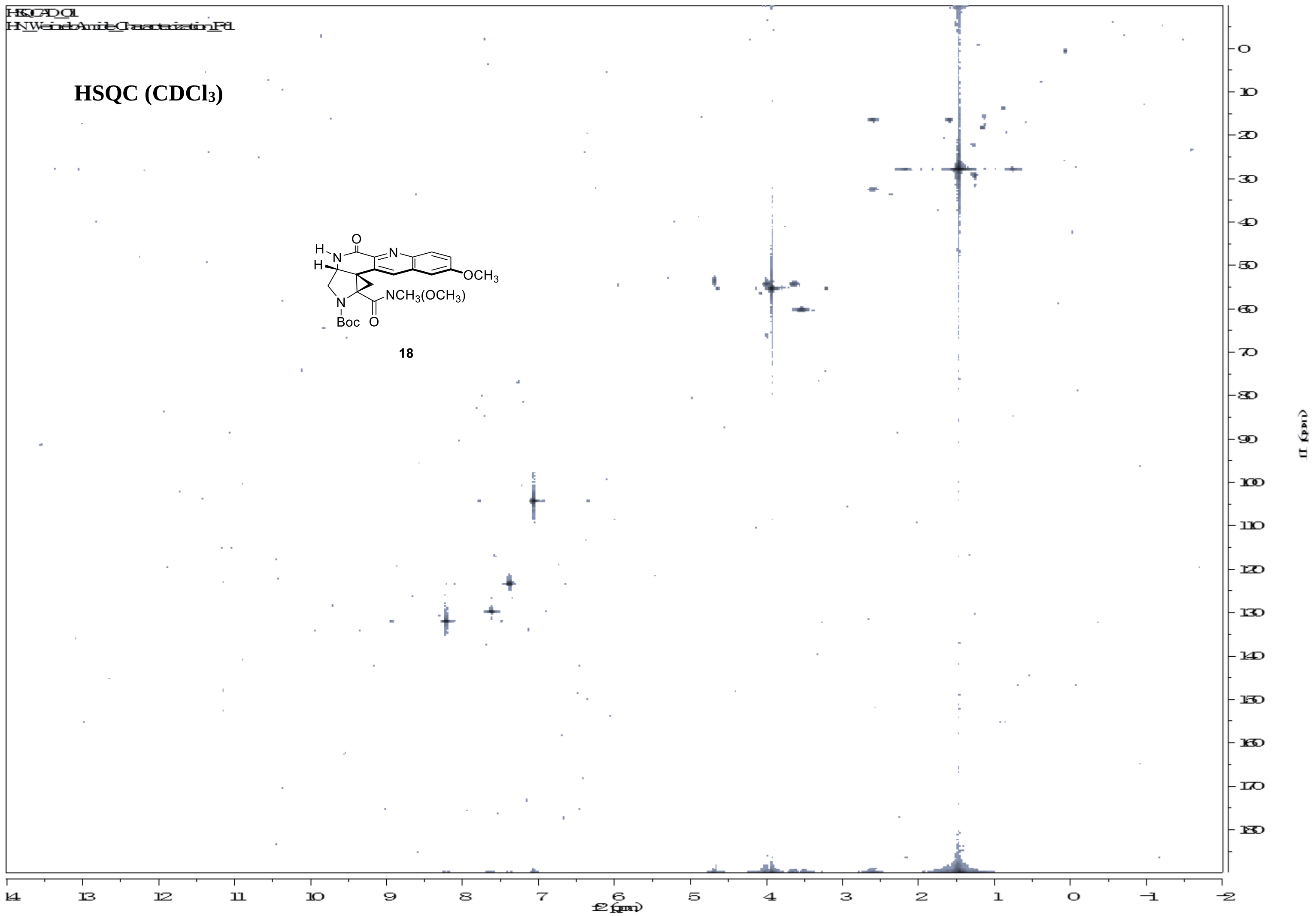
HSQC (CDCl3)
OCH3
NCH3(OCH3)
Boc
18
f2 (ppm)
f1 (ppm)

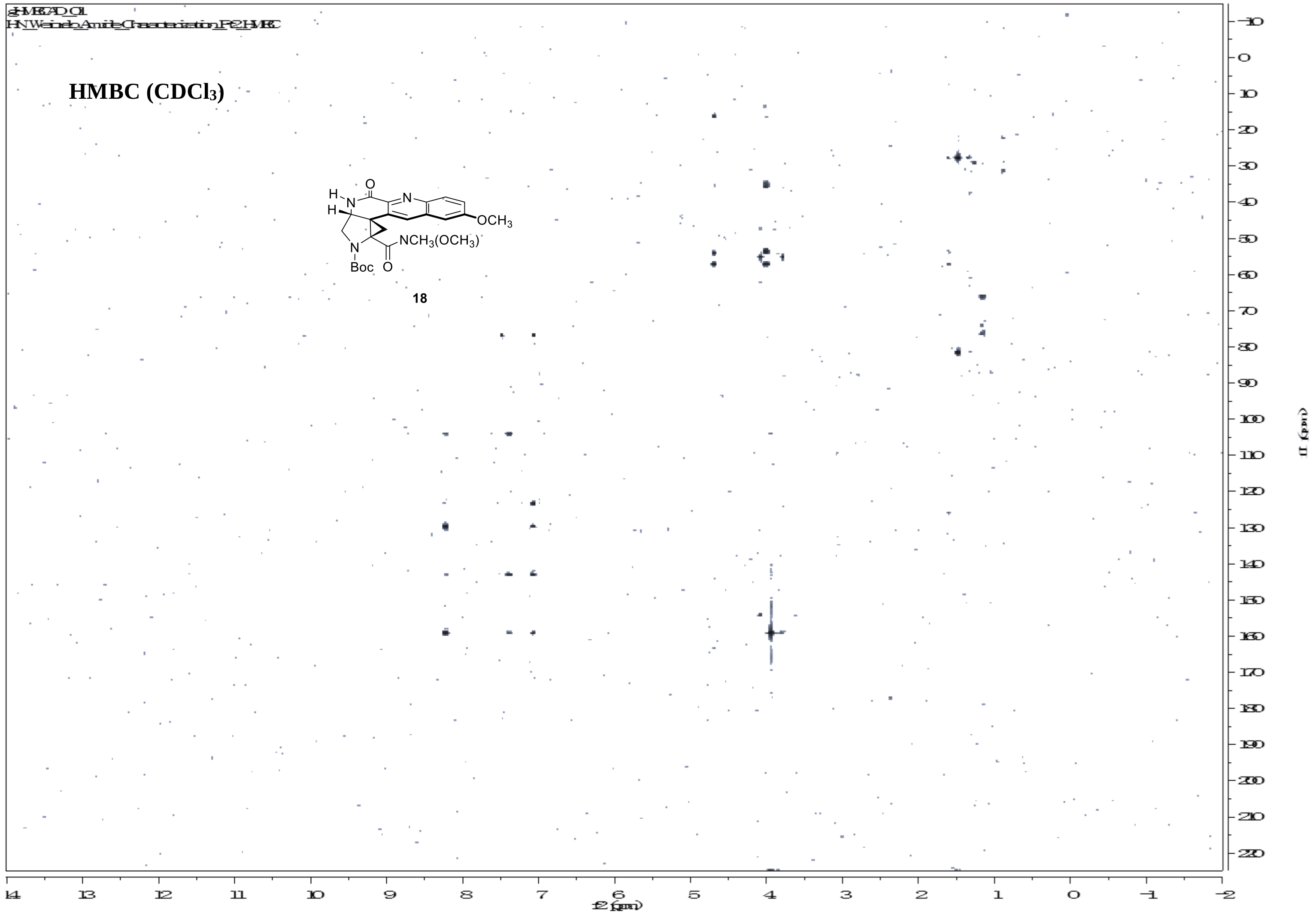
HMBC (CDCl3)
OCH3
NCH3(OCH3)
Boc
18
f2 (ppm)
f1 (ppm)

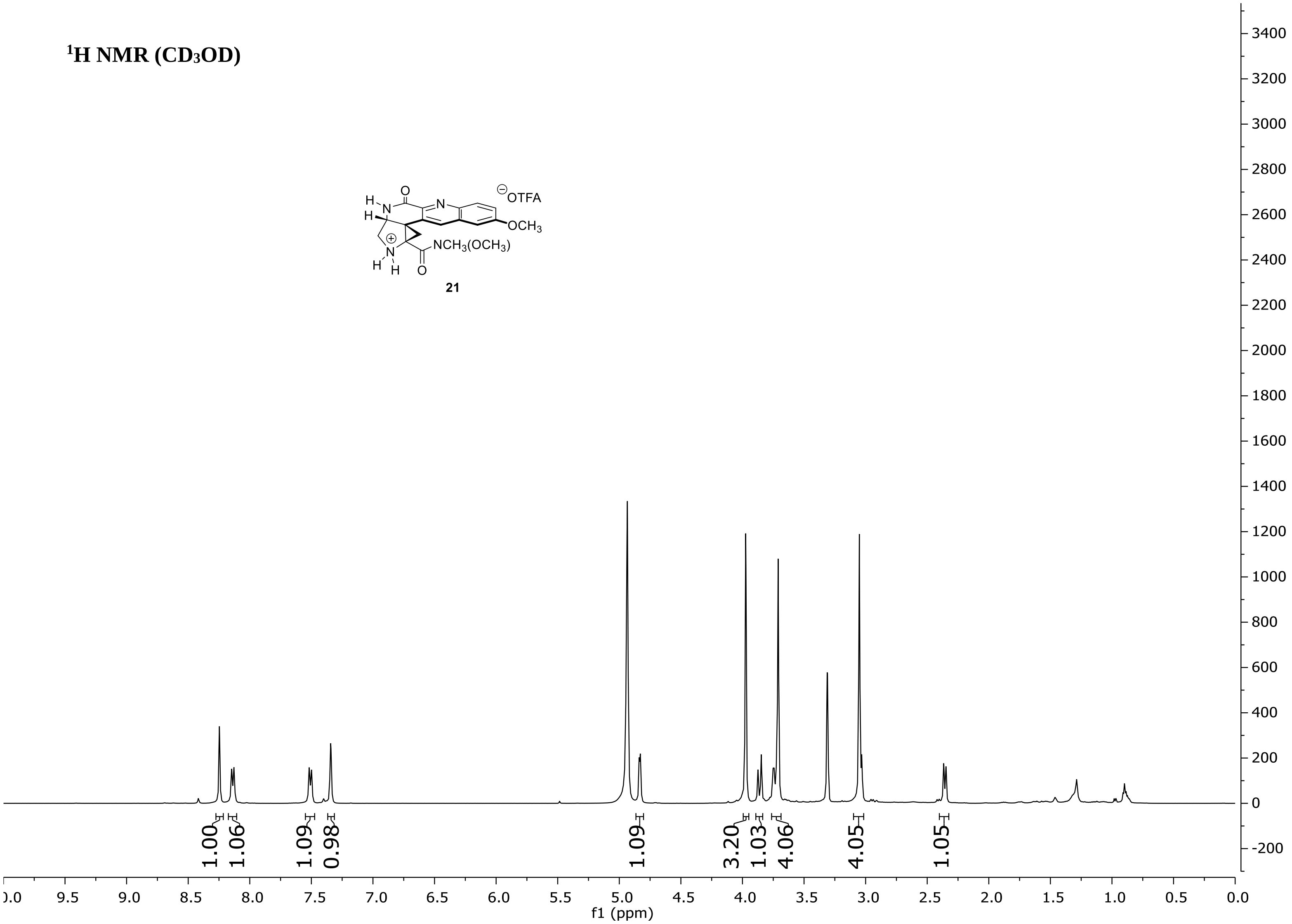

¹H NMR (CD3OD)
OTFA
OCH3
NCH3(OCH3)
21
1.00
1.06
1.09
0.98
1.09
3.20
1.03
4.06
4.05
1.05
f1 (ppm)

^{13}C NMR (CD_3OD)

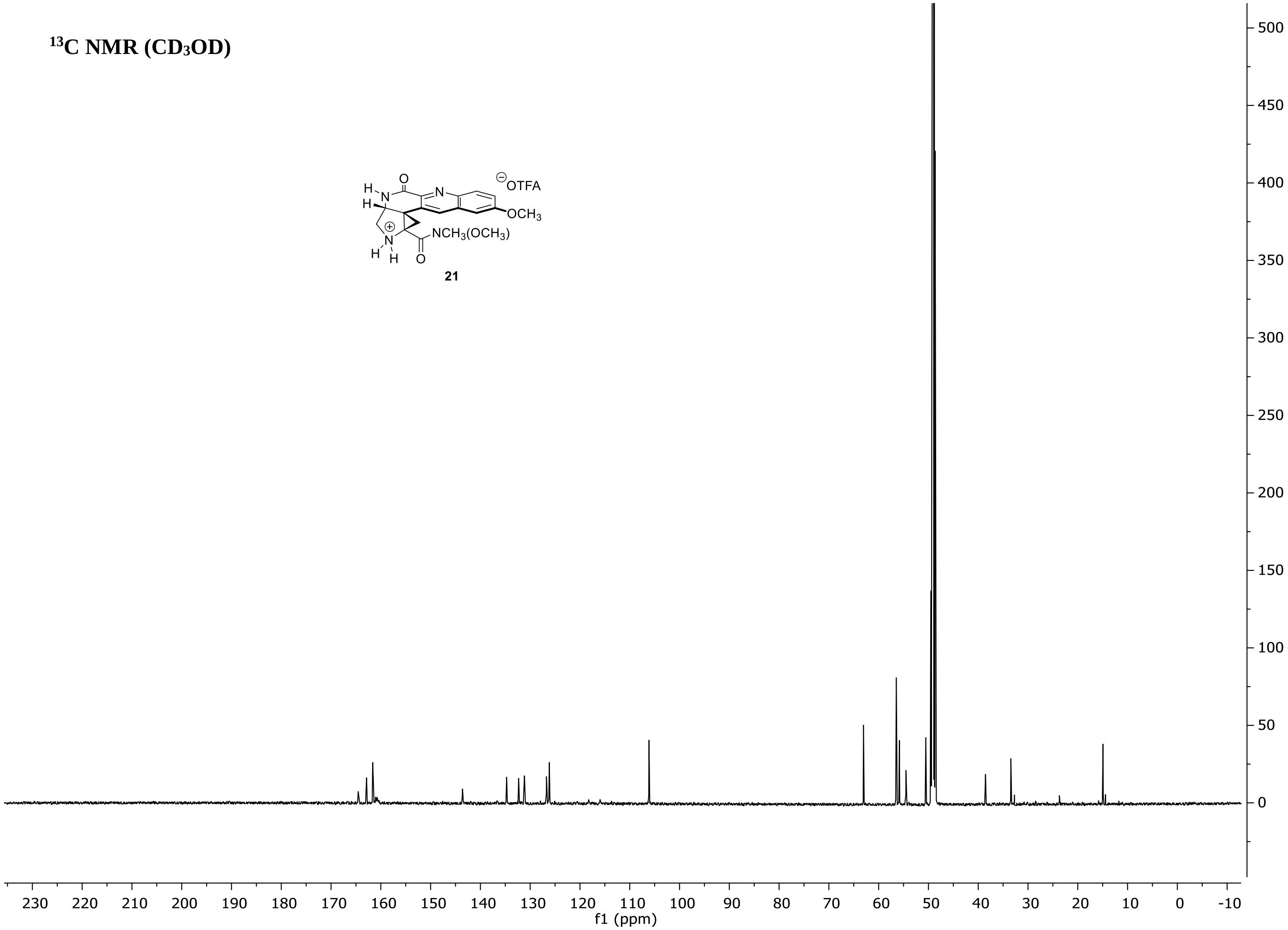

^{19}F NMR (CD_3OD)

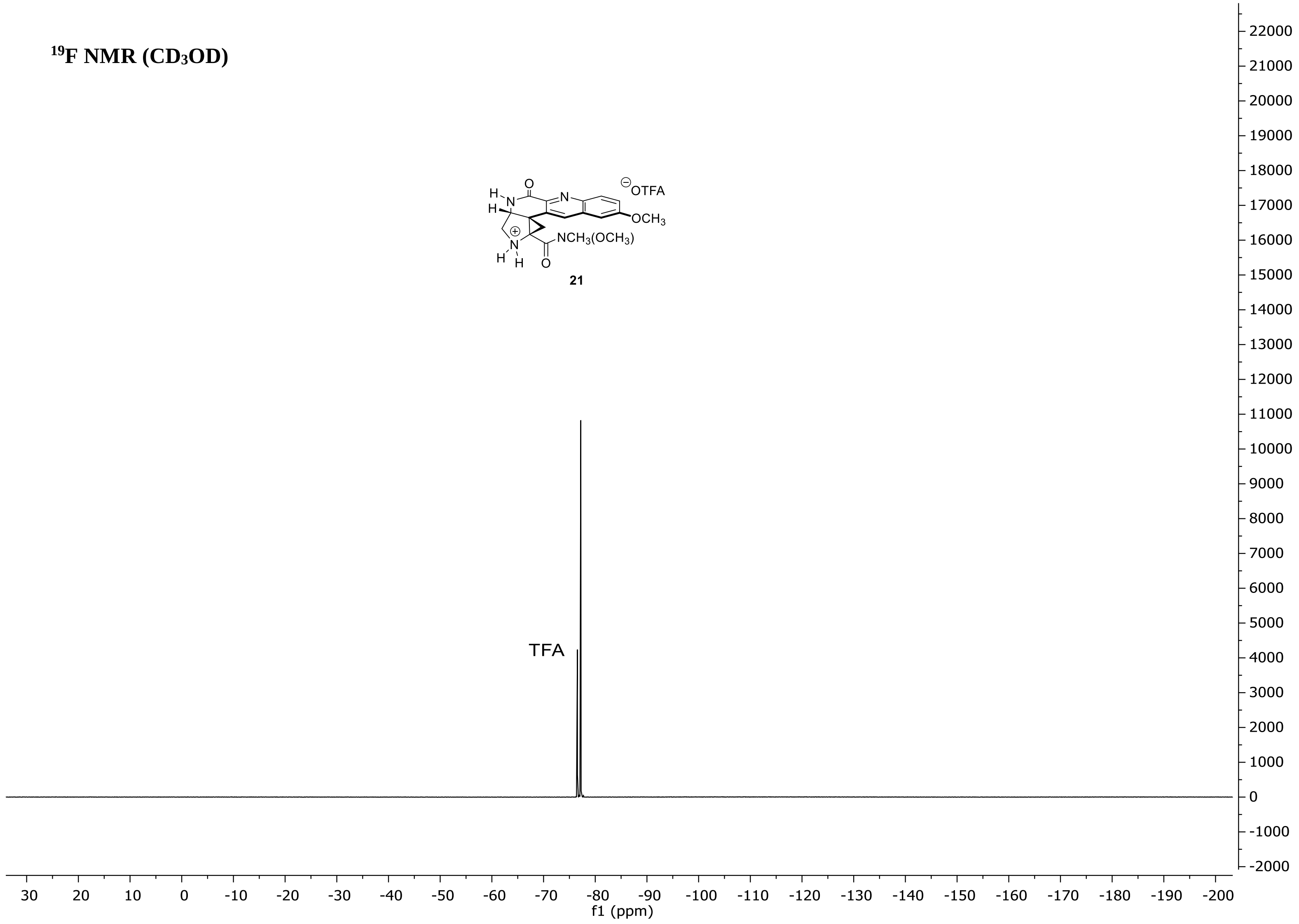

HSQC (CD_3OD)

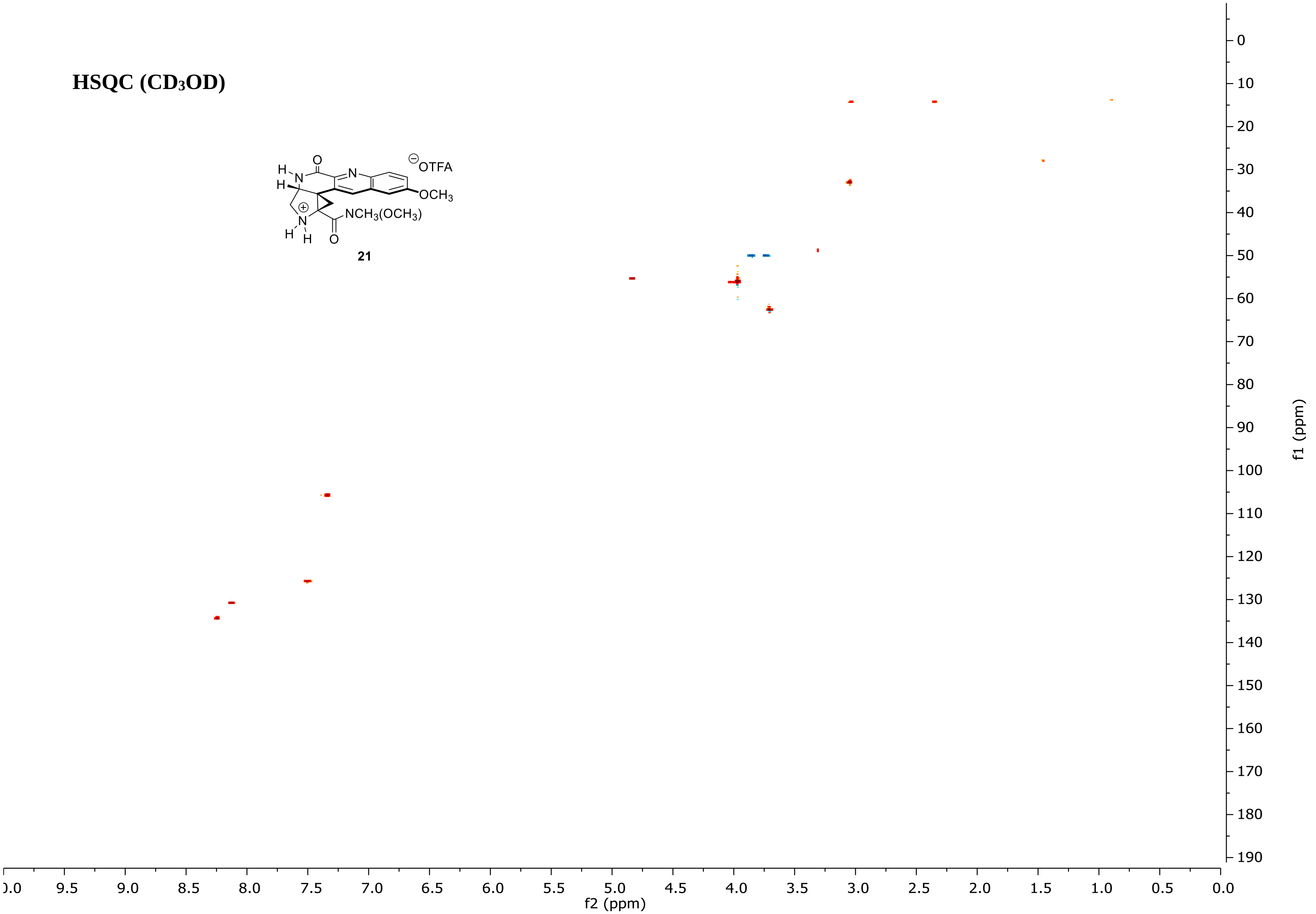

HMBC (CD_3OD)

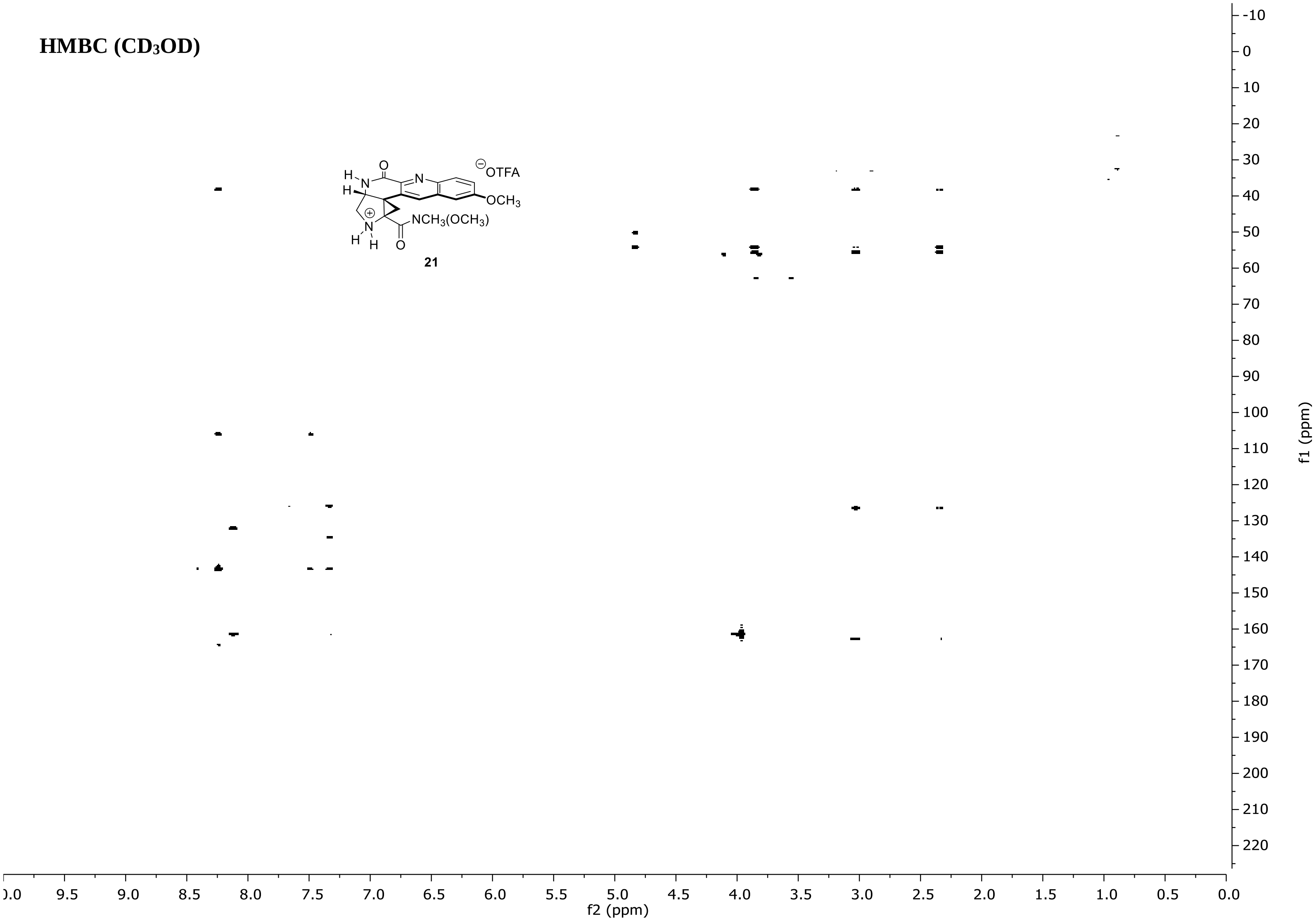

Crystallographic data for the Weinreb amide 18 (Rint = 0.192). Low-temperature diffraction data (ω-scans) were collected on a Rigaku MicroMax-007HF diffractometer coupled to a Saturn994+ CCD detector with Cu Kα (λ = 1.54178 Å) for the structure of 007-14186. The diffraction images were processed and scaled using Rigaku Oxford Diffraction software (CrysAlisPro; Rigaku OD: The Woodlands, TX, 2015). The structure was solved with SHELXT and was refined against F^2 on all data by full-matrix least squares with SHELXL (Sheldrick, G. M. Acta Cryst. 2008, A64, 112–122). All non-hydrogen atoms were refined anisotropically. Hydrogen atoms were included in the model at geometrically calculated positions and refined using a riding model. The isotropic displacement parameters of all hydrogen atoms were fixed to 1.2 times the U value of the atoms to which they are linked (1.5 times for methyl groups). The only exception is H3, which is freely refining and a part of a hydrogen bond between N3 and O3. The crystal structure reported here contains solvent accessible voids in the unit cell. In spite of numerous attempts, no sensible solvent model could be established, and the solvent is assumed to be disordered within these voids. The crystals had been obtained from a ethyl acetate. The program SQUEEZE was used to compensate for the contribution of disordered solvents contained in voids within the crystal lattice from the diffraction intensities. This procedure was applied to the data file and the submitted model is based on the solvent removed data. Based on the total electron density found in the voids, it is likely that 2 molecules of ethyl acetate molecules are present in the unit cell. See "_platon_squeeze_details" in the .cif for more information. The full numbering scheme of compound 007-14186 can be found in the full details of the X-ray structure determination (CIF), which is included as Supporting Information. CCDC number 1536046 (007-14186) contains the supplementary crystallographic data for this paper. These data can be obtained free of charge from The Cambridge Crystallographic Data Center via www.ccdc.cam.ac.uk/data_request/cif.

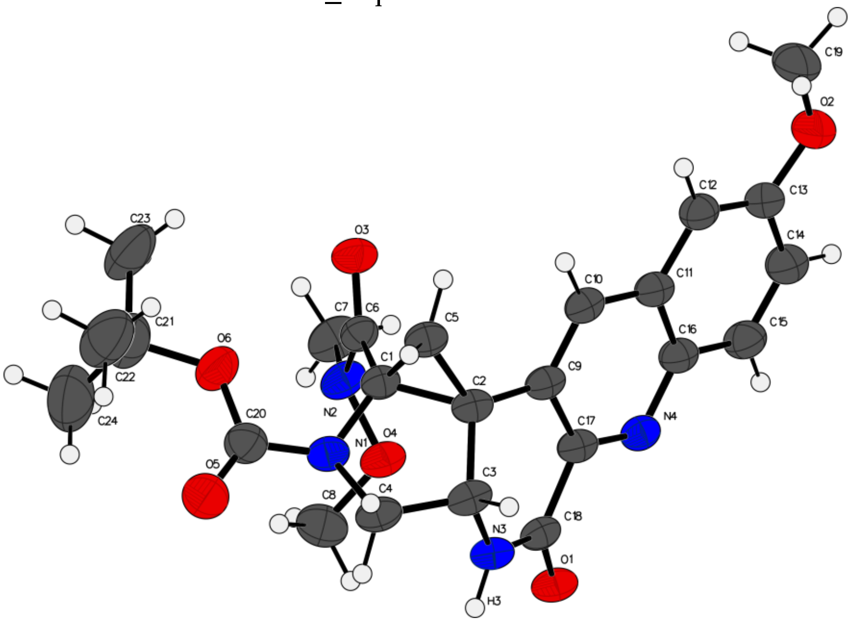

Figure S3. The complete numbering scheme of 007-14186 with 50% thermal ellipsoid probability levels. The hydrogen atoms are shown as circles for clarity.

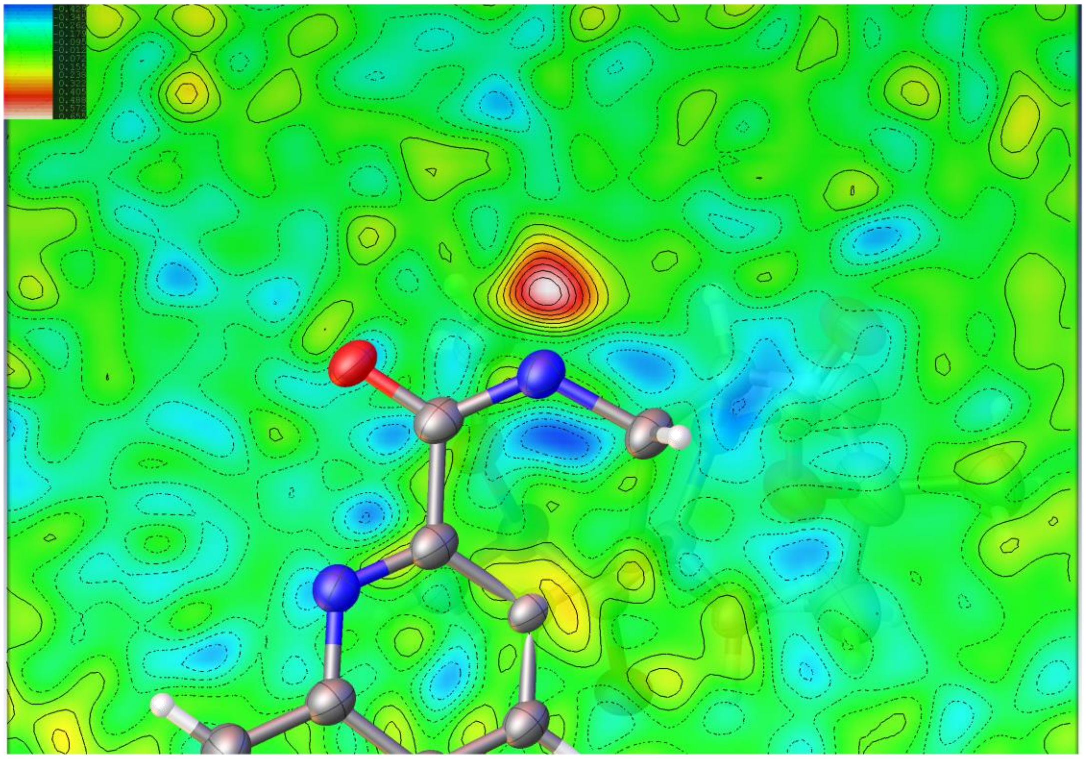

Figure S4. The Fourier difference map of total electron density in 007-14186 (0.8*e*/Å^3 isolines) highlights the plane of the amide {N3 C18 O1}, excluding contributions from H3 so as to show its effective position.

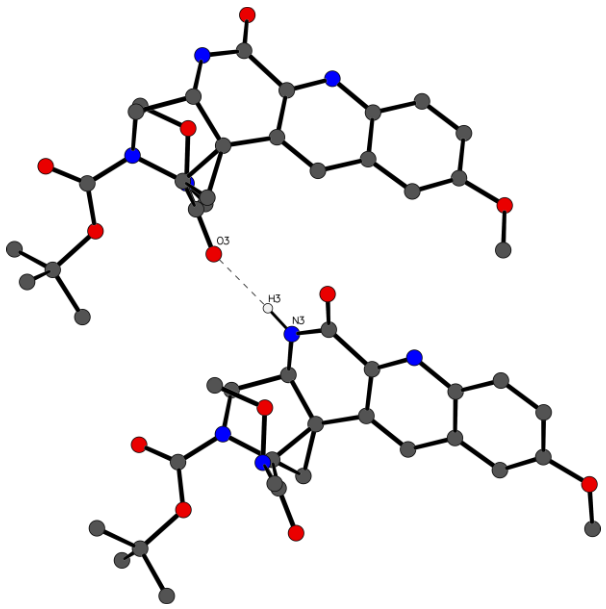

Figure S5. The hydrogen bonding in 007-14186 with atoms and H3 shown as circles for clarity; most hydrogen atoms are omitted. The hydrogen bond interaction is highlighted with a dashed line.

Table S8. Crystal data and structure refinement for 007-14186.

Identification code	007-14186	
Empirical formula	C24 H28 N4 O6	
Formula weight	468.50	
Temperature	93(2) K	
Wavelength	1.54184 Å	
Crystal system	Monoclinic	
Space group	$P2_1/c$	
Unit cell dimensions	a = 16.7641(7) Å	α= 90°.
	b = 11.6113(4) Å	β= 112.159(5)°.
	c = 14.8749(7) Å	γ = 90°.
Volume	2681.6(2) $Å^3$	
Z	4	
Density (calculated)	1.160 Mg/m^3	
Absorption coefficient	0.700 mm^{-1}	
F(000)	992	
Crystal size	0.180 x 0.030 x 0.020 mm^3	
Theta range for data collection	2.846 to 66.847°.	
Index ranges	-19<=h<=19, -13<=k<=13, -17<=l<=17	
Reflections collected	81113	
Independent reflections	4754 [R(int) = 0.1919]	
Completeness to theta = 66.847°	99.9 %	
Absorption correction	Semi-empirical from equivalents	
Max. and min. transmission	1.00000 and 0.40982	
Refinement method	Full-matrix least-squares on F^2	
Data / restraints / parameters	4754 / 0 / 317	
Goodness-of-fit on F^2	1.020	
Final R indices [I>2sigma(I)]	R1 = 0.0604, wR2 = 0.1491	
R indices (all data)	R1 = 0.0950, wR2 = 0.1691	
Largest diff. peak and hole	0.292 and -0.235 e.$Å^{-3}$	

Table S9. Atomic coordinates (x 10^4) and equivalent isotropic displacement parameters (Å^{2}x 10^3) for 007-14186. U(eq) is defined as one third of the trace of the orthogonalized U^{ij} tensor.

	x	y	z	U(eq)
O(1)	5556(1)	3510(2)	1736(1)	40(1)
O(2)	8708(1)	2799(2)	7514(1)	60(1)
O(3)	3878(1)	3182(2)	4765(1)	44(1)
O(4)	4044(1)	4081(2)	2638(1)	42(1)
O(5)	1752(1)	1397(2)	1591(2)	64(1)
O(6)	2175(1)	2277(2)	3074(2)	57(1)
N(1)	3146(1)	1874(2)	2444(2)	37(1)
N(2)	3746(2)	4143(2)	3400(2)	42(1)
N(3)	4725(2)	2026(2)	1835(2)	37(1)
N(4)	6596(1)	3237(2)	3658(2)	35(1)
C(1)	3804(2)	2049(2)	3397(2)	34(1)
C(2)	4636(2)	1527(2)	3384(2)	34(1)
C(3)	4427(2)	1138(2)	2345(2)	38(1)
C(4)	3456(2)	1044(2)	1889(2)	40(1)
C(5)	4162(2)	972(2)	3959(2)	38(1)
C(6)	3820(2)	3157(2)	3918(2)	34(1)
C(7)	3886(2)	5265(2)	3878(2)	53(1)
C(8)	3382(2)	4427(3)	1740(2)	58(1)
C(9)	5498(2)	2049(2)	3881(2)	34(1)
C(10)	5984(2)	1925(2)	4847(2)	35(1)
C(11)	6804(2)	2443(2)	5255(2)	36(1)
C(12)	7347(2)	2319(2)	6240(2)	40(1)
C(13)	8120(2)	2867(3)	6583(2)	46(1)
C(14)	8380(2)	3596(3)	5975(2)	50(1)
C(15)	7869(2)	3724(3)	5021(2)	45(1)
C(16)	7074(2)	3136(2)	4625(2)	36(1)
C(17)	5848(2)	2703(2)	3305(2)	34(1)
C(18)	5366(2)	2786(2)	2233(2)	35(1)
C(19)	8503(2)	2058(4)	8160(2)	75(1)
C(20)	2301(2)	1814(3)	2313(2)	47(1)

C(21)	1310(2)	2359(4)	3119(3)	73(1)
C(22)	908(3)	1170(4)	3031(3)	91(1)
C(23)	1521(3)	2852(4)	4126(3)	87(1)
C(24)	770(3)	3162(5)	2347(4)	102(2)

Table S10. Bond lengths [Å] and angles [°] for 007-14186.

O(1)-C(18)	1.237(3)
O(2)-C(13)	1.366(3)
O(2)-C(19)	1.425(4)
O(3)-C(6)	1.227(3)
O(4)-N(2)	1.402(3)
O(4)-C(8)	1.434(3)
O(5)-C(20)	1.220(3)
O(6)-C(20)	1.340(3)
O(6)-C(21)	1.480(4)
N(1)-C(20)	1.356(3)
N(1)-C(1)	1.445(3)
N(1)-C(4)	1.485(3)
N(2)-C(6)	1.359(3)
N(2)-C(7)	1.460(3)
N(3)-C(18)	1.343(3)
N(3)-C(3)	1.474(3)
N(3)-H(3)	0.90(3)
N(4)-C(17)	1.317(3)
N(4)-C(16)	1.362(3)
C(1)-C(6)	1.497(3)
C(1)-C(5)	1.498(4)
C(1)-C(2)	1.528(3)
C(2)-C(9)	1.481(4)
C(2)-C(5)	1.514(3)
C(2)-C(3)	1.519(4)
C(3)-C(4)	1.513(4)
C(3)-H(3A)	1.0000
C(4)-H(4A)	0.9900
C(4)-H(4B)	0.9900
C(5)-H(5A)	0.9900
C(5)-H(5B)	0.9900
C(7)-H(7A)	0.9800
C(7)-H(7B)	0.9800

C(7)-H(7C)	0.9800
C(8)-H(8A)	0.9800
C(8)-H(8B)	0.9800
C(8)-H(8C)	0.9800
C(9)-C(10)	1.366(4)
C(9)-C(17)	1.426(3)
C(10)-C(11)	1.410(4)
C(10)-H(10)	0.9500
C(11)-C(12)	1.411(4)
C(11)-C(16)	1.432(4)
C(12)-C(13)	1.358(4)
C(12)-H(12)	0.9500
C(13)-C(14)	1.421(4)
C(14)-C(15)	1.361(4)
C(14)-H(14)	0.9500
C(15)-C(16)	1.413(4)
C(15)-H(15)	0.9500
C(17)-C(18)	1.495(4)
C(19)-H(19A)	0.9800
C(19)-H(19B)	0.9800
C(19)-H(19C)	0.9800
C(21)-C(24)	1.491(6)
C(21)-C(23)	1.516(5)
C(21)-C(22)	1.520(6)
C(22)-H(22A)	0.9800
C(22)-H(22B)	0.9800
C(22)-H(22C)	0.9800
C(23)-H(23A)	0.9800
C(23)-H(23B)	0.9800
C(23)-H(23C)	0.9800
C(24)-H(24A)	0.9800
C(24)-H(24B)	0.9800
C(24)-H(24C)	0.9800

C(13)-O(2)-C(19)	116.9(2)

N(2)-O(4)-C(8)	110.7(2)
C(20)-O(6)-C(21)	122.3(2)
C(20)-N(1)-C(1)	121.2(2)
C(20)-N(1)-C(4)	116.9(2)
C(1)-N(1)-C(4)	109.8(2)
C(6)-N(2)-O(4)	115.5(2)
C(6)-N(2)-C(7)	120.9(2)
O(4)-N(2)-C(7)	113.5(2)
C(18)-N(3)-C(3)	127.1(2)
C(18)-N(3)-H(3)	117.5(18)
C(3)-N(3)-H(3)	115.2(18)
C(17)-N(4)-C(16)	118.3(2)
N(1)-C(1)-C(6)	119.1(2)
N(1)-C(1)-C(5)	115.3(2)
C(6)-C(1)-C(5)	120.2(2)
N(1)-C(1)-C(2)	107.2(2)
C(6)-C(1)-C(2)	120.6(2)
C(5)-C(1)-C(2)	60.01(16)
C(9)-C(2)-C(5)	120.9(2)
C(9)-C(2)-C(3)	115.9(2)
C(5)-C(2)-C(3)	118.6(2)
C(9)-C(2)-C(1)	123.7(2)
C(5)-C(2)-C(1)	59.03(16)
C(3)-C(2)-C(1)	105.6(2)
N(3)-C(3)-C(4)	109.7(2)
N(3)-C(3)-C(2)	108.8(2)
C(4)-C(3)-C(2)	105.9(2)
N(3)-C(3)-H(3A)	110.8
C(4)-C(3)-H(3A)	110.8
C(2)-C(3)-H(3A)	110.8
N(1)-C(4)-C(3)	104.3(2)
N(1)-C(4)-H(4A)	110.9
C(3)-C(4)-H(4A)	110.9
N(1)-C(4)-H(4B)	110.9
C(3)-C(4)-H(4B)	110.9

H(4A)-C(4)-H(4B)	108.9
C(1)-C(5)-C(2)	60.97(17)
C(1)-C(5)-H(5A)	117.7
C(2)-C(5)-H(5A)	117.7
C(1)-C(5)-H(5B)	117.7
C(2)-C(5)-H(5B)	117.7
H(5A)-C(5)-H(5B)	114.8
O(3)-C(6)-N(2)	121.1(2)
O(3)-C(6)-C(1)	122.1(2)
N(2)-C(6)-C(1)	116.8(2)
N(2)-C(7)-H(7A)	109.5
N(2)-C(7)-H(7B)	109.5
H(7A)-C(7)-H(7B)	109.5
N(2)-C(7)-H(7C)	109.5
H(7A)-C(7)-H(7C)	109.5
H(7B)-C(7)-H(7C)	109.5
O(4)-C(8)-H(8A)	109.5
O(4)-C(8)-H(8B)	109.5
H(8A)-C(8)-H(8B)	109.5
O(4)-C(8)-H(8C)	109.5
H(8A)-C(8)-H(8C)	109.5
H(8B)-C(8)-H(8C)	109.5
C(10)-C(9)-C(17)	118.1(2)
C(10)-C(9)-C(2)	124.4(2)
C(17)-C(9)-C(2)	117.6(2)
C(9)-C(10)-C(11)	120.4(2)
C(9)-C(10)-H(10)	119.8
C(11)-C(10)-H(10)	119.8
C(10)-C(11)-C(12)	123.1(2)
C(10)-C(11)-C(16)	117.1(2)
C(12)-C(11)-C(16)	119.8(2)
C(13)-C(12)-C(11)	119.5(3)
C(13)-C(12)-H(12)	120.2
C(11)-C(12)-H(12)	120.2
C(12)-C(13)-O(2)	125.2(3)

C(12)-C(13)-C(14)	121.2(3)
O(2)-C(13)-C(14)	113.7(3)
C(15)-C(14)-C(13)	120.4(3)
C(15)-C(14)-H(14)	119.8
C(13)-C(14)-H(14)	119.8
C(14)-C(15)-C(16)	120.3(3)
C(14)-C(15)-H(15)	119.8
C(16)-C(15)-H(15)	119.8
N(4)-C(16)-C(15)	119.1(2)
N(4)-C(16)-C(11)	122.2(2)
C(15)-C(16)-C(11)	118.7(2)
N(4)-C(17)-C(9)	123.8(2)
N(4)-C(17)-C(18)	116.4(2)
C(9)-C(17)-C(18)	119.7(2)
O(1)-C(18)-N(3)	121.8(2)
O(1)-C(18)-C(17)	121.6(2)
N(3)-C(18)-C(17)	116.6(2)
O(2)-C(19)-H(19A)	109.5
O(2)-C(19)-H(19B)	109.5
H(19A)-C(19)-H(19B)	109.5
O(2)-C(19)-H(19C)	109.5
H(19A)-C(19)-H(19C)	109.5
H(19B)-C(19)-H(19C)	109.5
O(5)-C(20)-O(6)	126.5(3)
O(5)-C(20)-N(1)	123.2(3)
O(6)-C(20)-N(1)	110.3(2)
O(6)-C(21)-C(24)	109.0(3)
O(6)-C(21)-C(23)	101.6(3)
C(24)-C(21)-C(23)	111.9(4)
O(6)-C(21)-C(22)	110.4(3)
C(24)-C(21)-C(22)	112.4(4)
C(23)-C(21)-C(22)	111.0(4)
C(21)-C(22)-H(22A)	109.5
C(21)-C(22)-H(22B)	109.5
H(22A)-C(22)-H(22B)	109.5

C(21)-C(22)-H(22C)	109.5
H(22A)-C(22)-H(22C)	109.5
H(22B)-C(22)-H(22C)	109.5
C(21)-C(23)-H(23A)	109.5
C(21)-C(23)-H(23B)	109.5
H(23A)-C(23)-H(23B)	109.5
C(21)-C(23)-H(23C)	109.5
H(23A)-C(23)-H(23C)	109.5
H(23B)-C(23)-H(23C)	109.5
C(21)-C(24)-H(24A)	109.5
C(21)-C(24)-H(24B)	109.5
H(24A)-C(24)-H(24B)	109.5
C(21)-C(24)-H(24C)	109.5
H(24A)-C(24)-H(24C)	109.5
H(24B)-C(24)-H(24C)	109.5

Symmetry transformations used to generate equivalent atoms:

Table S11. Anisotropic displacement parameters ($Å^2$x 10^3) for 007-14186. The anisotropic displacement factor exponent takes the form: $-2\pi^2[h^2 a^{*2}U^{11} + ... + 2 h k a^* b^* U^{12}]$

	U^{11}	U^{22}	U^{33}	U^{23}	U^{13}	U^{12}
O(1)	58(1)	34(1)	36(1)	4(1)	26(1)	-2(1)
O(2)	48(1)	92(2)	40(1)	11(1)	16(1)	-3(1)
O(3)	58(1)	43(1)	35(1)	-6(1)	20(1)	3(1)
O(4)	57(1)	36(1)	40(1)	3(1)	27(1)	2(1)
O(5)	52(1)	83(2)	54(1)	-23(1)	16(1)	-12(1)
O(6)	48(1)	73(2)	57(1)	-23(1)	27(1)	-8(1)
N(1)	46(1)	34(1)	34(1)	-5(1)	18(1)	-4(1)
N(2)	58(1)	30(1)	46(1)	-2(1)	31(1)	4(1)
N(3)	51(1)	34(1)	31(1)	-1(1)	20(1)	-1(1)
N(4)	44(1)	31(1)	36(1)	-1(1)	20(1)	1(1)
C(1)	41(1)	31(1)	32(1)	-1(1)	17(1)	0(1)
C(2)	47(1)	25(1)	32(1)	2(1)	19(1)	1(1)
C(3)	56(2)	27(1)	38(2)	1(1)	27(1)	1(1)
C(4)	56(2)	32(1)	37(1)	-7(1)	24(1)	-7(1)
C(5)	50(2)	32(1)	36(1)	0(1)	21(1)	-1(1)
C(6)	38(1)	34(1)	34(1)	-5(1)	16(1)	-1(1)
C(7)	78(2)	29(2)	64(2)	-7(1)	41(2)	0(1)
C(8)	69(2)	54(2)	47(2)	11(2)	19(2)	2(2)
C(9)	48(1)	25(1)	34(1)	2(1)	22(1)	6(1)
C(10)	45(1)	28(1)	37(2)	3(1)	22(1)	5(1)
C(11)	46(1)	31(1)	34(1)	3(1)	19(1)	7(1)
C(12)	45(1)	42(2)	37(2)	7(1)	20(1)	7(1)
C(13)	44(2)	62(2)	34(2)	5(1)	17(1)	7(1)
C(14)	47(2)	61(2)	42(2)	1(2)	19(1)	-3(1)
C(15)	48(2)	49(2)	41(2)	2(1)	22(1)	-4(1)
C(16)	44(1)	34(1)	35(2)	3(1)	19(1)	4(1)
C(17)	47(1)	27(1)	33(1)	2(1)	20(1)	5(1)
C(18)	46(1)	31(1)	34(1)	-2(1)	23(1)	2(1)
C(19)	56(2)	123(3)	43(2)	28(2)	16(2)	-4(2)
C(20)	46(2)	51(2)	43(2)	-7(1)	17(1)	-6(1)

C(21)	50(2)	89(3)	83(3)	-31(2)	31(2)	-8(2)
C(22)	72(2)	119(4)	98(3)	-38(3)	49(2)	-31(2)
C(23)	69(2)	115(4)	96(3)	-49(3)	53(2)	-18(2)
C(24)	67(2)	123(4)	115(4)	-19(3)	32(3)	23(3)

Table S12. Hydrogen coordinates (x 10^4) and isotropic displacement parameters ($Å^2$x 10^3) for 007-14186.

	x	y	z	U(eq)
H(3)	4473(17)	2010(20)	1180(20)	39(8)
H(3A)	4703	378	2329	45
H(4A)	3241	1253	1193	48
H(4B)	3265	252	1953	48
H(5A)	3855	241	3710	45
H(5B)	4424	1032	4676	45
H(7A)	4506	5403	4206	79
H(7B)	3613	5278	4355	79
H(7C)	3634	5868	3392	79
H(8A)	3590	4334	1211	86
H(8B)	3235	5236	1784	86
H(8C)	2870	3947	1613	86
H(10)	5770	1486	5246	42
H(12)	7173	1856	6660	48
H(14)	8912	3996	6237	60
H(15)	8049	4210	4619	54
H(19A)	8404	1276	7892	112
H(19B)	7982	2338	8240	112
H(19C)	8983	2050	8792	112
H(22A)	753	885	2367	137
H(22B)	389	1217	3185	137
H(22C)	1322	642	3486	137
H(23A)	1948	2361	4605	130
H(23B)	997	2885	4268	130
H(23C)	1757	3630	4156	130
H(24A)	1088	3878	2376	154
H(24B)	237	3334	2447	154
H(24C)	629	2802	1709	154

Table S13. Torsion angles [°] for 007-14186.

C(8)-O(4)-N(2)-C(6)	125.0(3)
C(8)-O(4)-N(2)-C(7)	-89.2(3)
C(20)-N(1)-C(1)-C(6)	-64.7(3)
C(4)-N(1)-C(1)-C(6)	154.1(2)
C(20)-N(1)-C(1)-C(5)	89.5(3)
C(4)-N(1)-C(1)-C(5)	-51.7(3)
C(20)-N(1)-C(1)-C(2)	153.9(2)
C(4)-N(1)-C(1)-C(2)	12.7(3)
N(1)-C(1)-C(2)-C(9)	141.6(2)
C(6)-C(1)-C(2)-C(9)	0.9(4)
C(5)-C(1)-C(2)-C(9)	-108.6(3)
N(1)-C(1)-C(2)-C(5)	-109.8(2)
C(6)-C(1)-C(2)-C(5)	109.5(3)
N(1)-C(1)-C(2)-C(3)	4.6(3)
C(6)-C(1)-C(2)-C(3)	-136.2(2)
C(5)-C(1)-C(2)-C(3)	114.4(2)
C(18)-N(3)-C(3)-C(4)	141.6(2)
C(18)-N(3)-C(3)-C(2)	26.2(3)
C(9)-C(2)-C(3)-N(3)	-42.6(3)
C(5)-C(2)-C(3)-N(3)	161.1(2)
C(1)-C(2)-C(3)-N(3)	98.3(2)
C(9)-C(2)-C(3)-C(4)	-160.4(2)
C(5)-C(2)-C(3)-C(4)	43.3(3)
C(1)-C(2)-C(3)-C(4)	-19.5(3)
C(20)-N(1)-C(4)-C(3)	-167.9(2)
C(1)-N(1)-C(4)-C(3)	-24.8(3)
N(3)-C(3)-C(4)-N(1)	-90.4(2)
C(2)-C(3)-C(4)-N(1)	26.8(3)
N(1)-C(1)-C(5)-C(2)	96.0(2)
C(6)-C(1)-C(5)-C(2)	-110.1(3)
C(9)-C(2)-C(5)-C(1)	113.3(3)
C(3)-C(2)-C(5)-C(1)	-91.7(2)
O(4)-N(2)-C(6)-O(3)	154.1(2)

C(7)-N(2)-C(6)-O(3)	11.0(4)
O(4)-N(2)-C(6)-C(1)	-27.5(3)
C(7)-N(2)-C(6)-C(1)	-170.6(2)
N(1)-C(1)-C(6)-O(3)	131.2(3)
C(5)-C(1)-C(6)-O(3)	-21.8(4)
C(2)-C(1)-C(6)-O(3)	-92.6(3)
N(1)-C(1)-C(6)-N(2)	-47.2(3)
C(5)-C(1)-C(6)-N(2)	159.8(2)
C(2)-C(1)-C(6)-N(2)	89.0(3)
C(5)-C(2)-C(9)-C(10)	10.6(4)
C(3)-C(2)-C(9)-C(10)	-145.1(2)
C(1)-C(2)-C(9)-C(10)	81.8(3)
C(5)-C(2)-C(9)-C(17)	-170.9(2)
C(3)-C(2)-C(9)-C(17)	33.4(3)
C(1)-C(2)-C(9)-C(17)	-99.7(3)
C(17)-C(9)-C(10)-C(11)	0.7(3)
C(2)-C(9)-C(10)-C(11)	179.2(2)
C(9)-C(10)-C(11)-C(12)	-178.1(2)
C(9)-C(10)-C(11)-C(16)	2.8(4)
C(10)-C(11)-C(12)-C(13)	-178.6(3)
C(16)-C(11)-C(12)-C(13)	0.5(4)
C(11)-C(12)-C(13)-O(2)	-178.8(3)
C(11)-C(12)-C(13)-C(14)	2.2(4)
C(19)-O(2)-C(13)-C(12)	2.6(5)
C(19)-O(2)-C(13)-C(14)	-178.2(3)
C(12)-C(13)-C(14)-C(15)	-2.6(5)
O(2)-C(13)-C(14)-C(15)	178.2(3)
C(13)-C(14)-C(15)-C(16)	0.2(4)
C(17)-N(4)-C(16)-C(15)	-178.6(2)
C(17)-N(4)-C(16)-C(11)	2.0(4)
C(14)-C(15)-C(16)-N(4)	-176.9(3)
C(14)-C(15)-C(16)-C(11)	2.4(4)
C(10)-C(11)-C(16)-N(4)	-4.3(4)
C(12)-C(11)-C(16)-N(4)	176.5(2)
C(10)-C(11)-C(16)-C(15)	176.4(2)

C(12)-C(11)-C(16)-C(15)	-2.8(4)
C(16)-N(4)-C(17)-C(9)	1.9(4)
C(16)-N(4)-C(17)-C(18)	-176.9(2)
C(10)-C(9)-C(17)-N(4)	-3.3(4)
C(2)-C(9)-C(17)-N(4)	178.1(2)
C(10)-C(9)-C(17)-C(18)	175.4(2)
C(2)-C(9)-C(17)-C(18)	-3.2(3)
C(3)-N(3)-C(18)-O(1)	-178.9(2)
C(3)-N(3)-C(18)-C(17)	2.0(4)
N(4)-C(17)-C(18)-O(1)	-15.4(3)
C(9)-C(17)-C(18)-O(1)	165.9(2)
N(4)-C(17)-C(18)-N(3)	163.7(2)
C(9)-C(17)-C(18)-N(3)	-15.1(3)
C(21)-O(6)-C(20)-O(5)	-1.3(5)
C(21)-O(6)-C(20)-N(1)	178.4(3)
C(1)-N(1)-C(20)-O(5)	-161.5(3)
C(4)-N(1)-C(20)-O(5)	-22.8(4)
C(1)-N(1)-C(20)-O(6)	18.9(4)
C(4)-N(1)-C(20)-O(6)	157.5(2)
C(20)-O(6)-C(21)-C(24)	-65.6(4)
C(20)-O(6)-C(21)-C(23)	176.2(3)
C(20)-O(6)-C(21)-C(22)	58.3(4)

Symmetry transformations used to generate equivalent atoms:

Table S14. Hydrogen bonds for 007-14186 [Å and °].

D-H...A	d(D-H)	d(H...A)	d(D...A)	<(DHA)
N(3)-H(3)...O(3)#1	0.90(3)	1.98(3)	2.874(3)	174(3)

Symmetry transformations used to generate equivalent atoms:

#1 x,-y+1/2,z-1/2

Crystallographic Data for the Weinreb amide 18 (Rint = 0.298).

Table S15. Crystal data and structure refinement for 007-14186.

Identification code	007-14186	
Empirical formula	$C_{24}H_{28}N_4O_6$	
Formula weight	468.50	
Temperature	93(2) K	
Wavelength	1.54187 Å	
Crystal system	Monoclinic	
Space group	$P\ 2_1/c$	
Unit cell dimensions	a = 16.7552(12) Å	α = 90°
	b = 11.6023(3) Å	β = 112.152(8)°
	c = 14.8679(4) Å	γ = 90°
Volume	2677.0(3) $Å^3$	
Z	4	
Density (calculated)	1.162 Mg/m^3	
Absorption coefficient	0.701 mm^{-1}	
F(000)	992	
Crystal size	0.180 x 0.030 x 0.020 mm^3	
Θ range for data collection	2.848 to 68.239°.	
Index ranges	$-20 \le h \le 20$, $-13 \le k \le 13$, $-17 \le l \le 17$	
Reflections collected	69124	
Independent reflections	4890 [R(int) = 0.2983]	
Completeness to θ = 67.687°	100.0 %	
Absorption correction	Semi-empirical from equivalents	
Max. and min. transmission	0.986 and 0.652	
Refinement method	Full-matrix least-squares on F^2	
Data / restraints / parameters	4890 / 0 / 317	
Goodness-of-fit on F^2	0.798	
Final R indices [I>2σ(I)]	R1 = 0.0612, wR2 = 0.1203	
R indices (all data)	R1 = 0.1627, wR2 = 0.1550	
Largest diff. peak and hole	0.294 and -0.197 e.$Å^{-3}$	

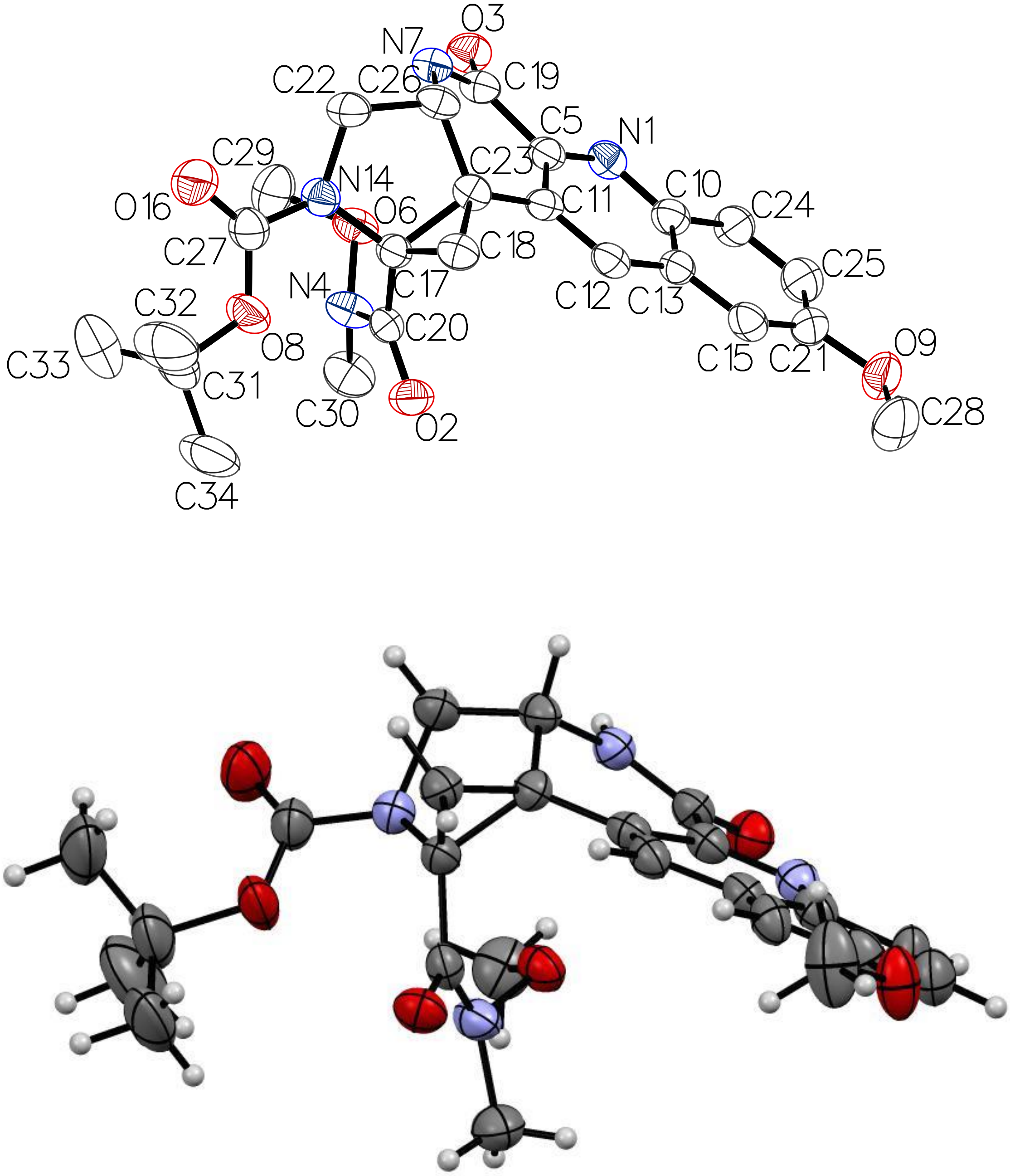

Figure S6. The full numbering scheme of **007-14186**. All atoms shown are depicted with 50% thermal contours. The hydrogen atoms are removed for clarity. Atom C17 has a chirality of *R*; C23 has *S*; C26 has *R*.

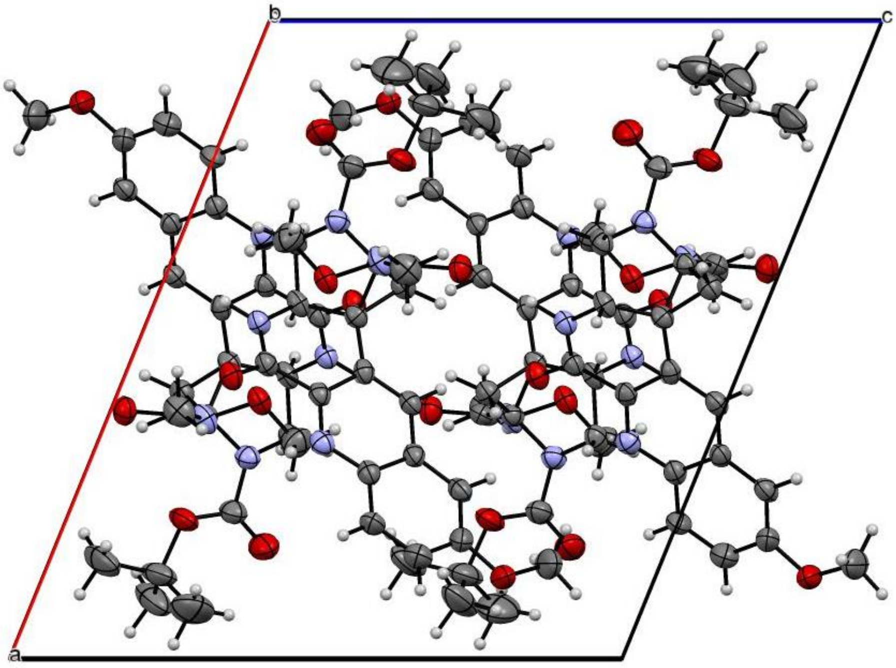

Packing diagram along the crystallographic *a*-axis

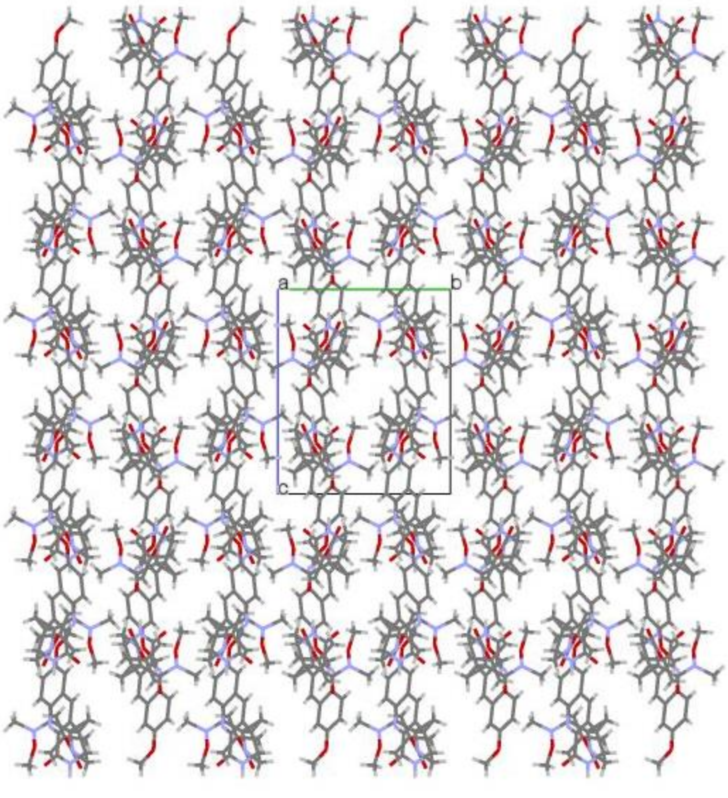

Packing diagram along the crystallographic *b*-axis

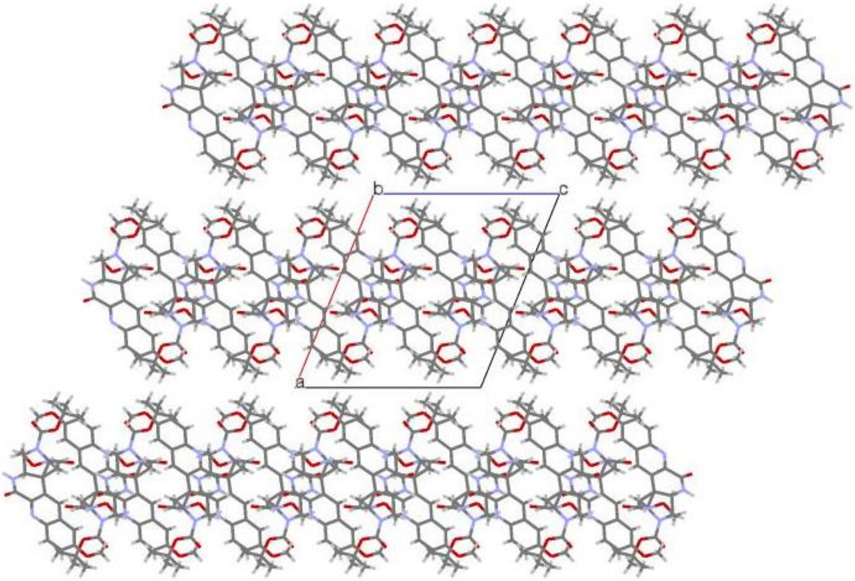

Packing diagram along the crystallographic *c*-axis

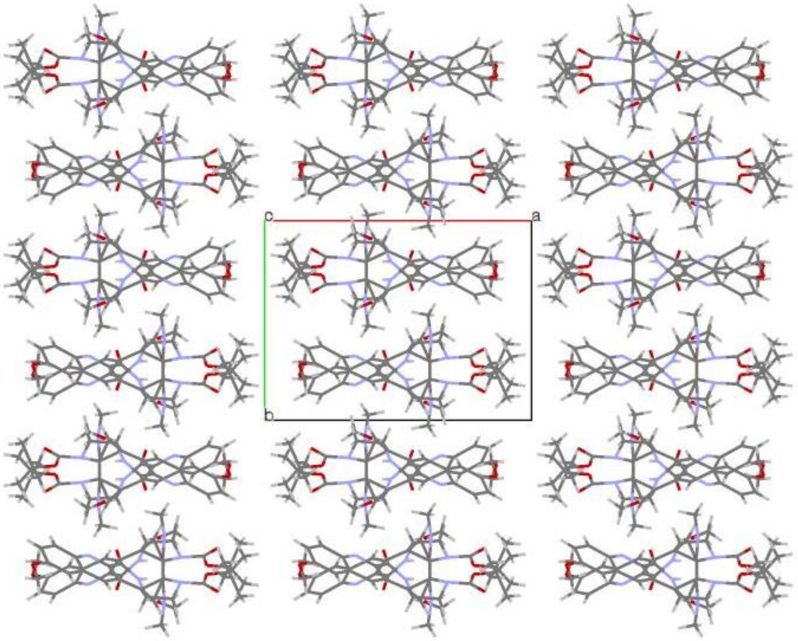

Table S16. Atomic coordinates (Å x 10^4) and equivalent isotropic displacement parameters (Å^{2}x 10^3) for 007-14186. U(eq) is defined as one third of the trace of the orthogonalized U^{ij} tensor.

	x	y	z	U(eq)
N(1)	3400(2)	6769(3)	1339(2)	38(1)
O(2)	6121(2)	6814(2)	234(2)	45(1)
O(3)	4445(2)	6489(2)	3268(2)	43(1)
N(4)	6247(2)	5865(3)	1594(2)	43(1)
C(5)	4157(2)	7303(3)	1697(3)	34(1)
O(6)	5960(2)	5915(2)	2366(2)	43(1)
N(7)	5276(2)	7969(3)	3166(2)	36(1)
O(8)	7826(2)	7718(3)	1924(2)	57(1)
O(9)	1292(2)	7198(3)	-2511(2)	60(1)
C(10)	2931(2)	6863(3)	375(3)	37(1)
C(11)	4510(2)	7959(3)	1124(3)	33(1)
C(12)	4015(2)	8070(3)	157(3)	36(1)
C(13)	3198(2)	7550(3)	-248(3)	35(1)
N(14)	6855(2)	8123(3)	2554(2)	38(1)
C(15)	2656(2)	7673(3)	-1229(3)	40(1)
O(16)	8254(2)	8597(3)	3412(2)	66(1)
C(17)	6198(2)	7946(3)	1604(3)	34(1)
C(18)	5837(2)	9033(3)	1047(3)	38(1)
C(19)	4632(2)	7209(3)	2769(3)	37(1)
C(20)	6182(2)	6851(3)	1082(3)	35(1)
C(21)	1878(3)	7132(4)	-1581(3)	47(1)
C(22)	6546(2)	8956(3)	3116(3)	41(1)
C(23)	5369(2)	8469(3)	1620(3)	33(1)
C(24)	2136(2)	6281(4)	-22(3)	45(1)
C(25)	1624(3)	6405(4)	-983(3)	52(1)
C(26)	5570(2)	8861(3)	2650(3)	36(1)
C(27)	7711(3)	8192(4)	2683(3)	47(1)
C(28)	1496(3)	7939(5)	-3168(3)	78(2)
C(29)	6617(3)	5575(4)	3260(3)	60(1)
C(30)	6117(3)	4733(3)	1126(3)	55(1)
C(31)	8685(3)	7632(5)	1885(4)	72(2)
C(32)	9083(3)	8831(5)	1963(4)	94(2)

C(33)	9222(4)	6828(6)	2653(5)	109(2)
C(34)	8473(3)	7142(5)	867(4)	94(2)

Table S17. Bond lengths [Å] and angles [°] for 007-14186.

N(1)-C(5)	1.329(4)
N(1)-C(10)	1.354(4)
O(2)-C(20)	1.227(4)
O(3)-C(19)	1.235(4)
N(4)-C(20)	1.356(5)
N(4)-O(6)	1.405(3)
N(4)-C(30)	1.463(5)
C(5)-C(11)	1.427(5)
C(5)-C(19)	1.492(5)
O(6)-C(29)	1.425(4)
N(7)-C(19)	1.345(5)
N(7)-C(26)	1.479(4)
N(7)-H(7)	0.97(4)
O(8)-C(27)	1.332(5)
O(8)-C(31)	1.466(5)
O(9)-C(21)	1.364(4)
O(9)-C(28)	1.435(5)
C(10)-C(24)	1.410(5)
C(10)-C(13)	1.417(5)
C(11)-C(12)	1.368(5)
C(11)-C(23)	1.472(5)
C(12)-C(13)	1.407(5)
C(12)-H(12)	0.9500
C(13)-C(15)	1.405(5)
N(14)-C(27)	1.375(5)
N(14)-C(17)	1.441(4)
N(14)-C(22)	1.492(4)
C(15)-C(21)	1.360(5)
C(15)-H(15)	0.9500
O(16)-C(27)	1.217(4)
C(17)-C(20)	1.485(5)
C(17)-C(18)	1.504(5)
C(17)-C(23)	1.523(5)
C(18)-C(23)	1.507(5)
C(18)-H(18A)	0.9900

C(18)-H(18B)	0.9900
C(21)-C(25)	1.404(6)
C(22)-C(26)	1.521(5)
C(22)-H(22A)	0.9900
C(22)-H(22B)	0.9900
C(23)-C(26)	1.509(5)
C(24)-C(25)	1.368(5)
C(24)-H(24)	0.9500
C(25)-H(25)	0.9500
C(26)-H(26)	1.0000
C(28)-H(28A)	0.9800
C(28)-H(28B)	0.9800
C(28)-H(28C)	0.9800
C(29)-H(29A)	0.9800
C(29)-H(29B)	0.9800
C(29)-H(29C)	0.9800
C(30)-H(30A)	0.9800
C(30)-H(30B)	0.9800
C(30)-H(30C)	0.9800
C(31)-C(33)	1.486(7)
C(31)-C(34)	1.527(6)
C(31)-C(32)	1.528(7)
C(32)-H(32A)	0.9800
C(32)-H(32B)	0.9800
C(32)-H(32C)	0.9800
C(33)-H(33A)	0.9800
C(33)-H(33B)	0.9800
C(33)-H(33C)	0.9800
C(34)-H(1A)	0.9800
C(34)-H(1B)	0.9800
C(34)-H(1C)	0.9800

C(5)-N(1)-C(10)	118.1(3)
C(20)-N(4)-O(6)	116.3(3)
C(20)-N(4)-C(30)	121.7(3)
O(6)-N(4)-C(30)	113.0(3)
N(1)-C(5)-C(11)	123.9(3)

N(1)-C(5)-C(19)	116.0(3)
C(11)-C(5)-C(19)	120.1(4)
N(4)-O(6)-C(29)	111.6(3)
C(19)-N(7)-C(26)	126.8(3)
C(19)-N(7)-H(7)	119(2)
C(26)-N(7)-H(7)	115(2)
C(27)-O(8)-C(31)	121.3(3)
C(21)-O(9)-C(28)	117.3(3)
N(1)-C(10)-C(24)	119.0(3)
N(1)-C(10)-C(13)	122.5(4)
C(24)-C(10)-C(13)	118.5(4)
C(12)-C(11)-C(5)	117.1(3)
C(12)-C(11)-C(23)	125.3(3)
C(5)-C(11)-C(23)	117.6(3)
C(11)-C(12)-C(13)	121.0(3)
C(11)-C(12)-H(12)	119.5
C(13)-C(12)-H(12)	119.5
C(15)-C(13)-C(12)	123.0(3)
C(15)-C(13)-C(10)	119.7(4)
C(12)-C(13)-C(10)	117.3(3)
C(27)-N(14)-C(17)	121.1(3)
C(27)-N(14)-C(22)	116.7(3)
C(17)-N(14)-C(22)	110.0(3)
C(21)-C(15)-C(13)	120.2(4)
C(21)-C(15)-H(15)	119.9
C(13)-C(15)-H(15)	119.9
N(14)-C(17)-C(20)	119.4(3)
N(14)-C(17)-C(18)	114.8(3)
C(20)-C(17)-C(18)	120.3(3)
N(14)-C(17)-C(23)	107.0(3)
C(20)-C(17)-C(23)	120.8(3)
C(18)-C(17)-C(23)	59.7(2)
C(17)-C(18)-C(23)	60.8(2)
C(17)-C(18)-H(18A)	117.7
C(23)-C(18)-H(18A)	117.7
C(17)-C(18)-H(18B)	117.7
C(23)-C(18)-H(18B)	117.7

H(18A)-C(18)-H(18B)	114.8
O(3)-C(19)-N(7)	121.5(4)
O(3)-C(19)-C(5)	122.4(4)
N(7)-C(19)-C(5)	116.0(4)
O(2)-C(20)-N(4)	120.4(4)
O(2)-C(20)-C(17)	123.1(4)
N(4)-C(20)-C(17)	116.5(3)
C(15)-C(21)-O(9)	125.7(4)
C(15)-C(21)-C(25)	120.6(4)
O(9)-C(21)-C(25)	113.7(4)
N(14)-C(22)-C(26)	103.8(3)
N(14)-C(22)-H(22A)	111.0
C(26)-C(22)-H(22A)	111.0
N(14)-C(22)-H(22B)	111.0
C(26)-C(22)-H(22B)	111.0
H(22A)-C(22)-H(22B)	109.0
C(11)-C(23)-C(18)	120.7(3)
C(11)-C(23)-C(26)	115.5(3)
C(18)-C(23)-C(26)	118.6(3)
C(11)-C(23)-C(17)	123.9(3)
C(18)-C(23)-C(17)	59.5(2)
C(26)-C(23)-C(17)	106.3(3)
C(25)-C(24)-C(10)	120.6(4)
C(25)-C(24)-H(24)	119.7
C(10)-C(24)-H(24)	119.7
C(24)-C(25)-C(21)	120.2(4)
C(24)-C(25)-H(25)	119.9
C(21)-C(25)-H(25)	119.9
N(7)-C(26)-C(23)	109.0(3)
N(7)-C(26)-C(22)	109.0(3)
C(23)-C(26)-C(22)	105.9(3)
N(7)-C(26)-H(26)	110.9
C(23)-C(26)-H(26)	110.9
C(22)-C(26)-H(26)	110.9
O(16)-C(27)-O(8)	127.9(4)
O(16)-C(27)-N(14)	122.5(4)
O(8)-C(27)-N(14)	109.5(4)

O(9)-C(28)-H(28A)	109.5
O(9)-C(28)-H(28B)	109.5
H(28A)-C(28)-H(28B)	109.5
O(9)-C(28)-H(28C)	109.5
H(28A)-C(28)-H(28C)	109.5
H(28B)-C(28)-H(28C)	109.5
O(6)-C(29)-H(29A)	109.5
O(6)-C(29)-H(29B)	109.5
H(29A)-C(29)-H(29B)	109.5
O(6)-C(29)-H(29C)	109.5
H(29A)-C(29)-H(29C)	109.5
H(29B)-C(29)-H(29C)	109.5
N(4)-C(30)-H(30A)	109.5
N(4)-C(30)-H(30B)	109.5
H(30A)-C(30)-H(30B)	109.5
N(4)-C(30)-H(30C)	109.5
H(30A)-C(30)-H(30C)	109.5
H(30B)-C(30)-H(30C)	109.5
O(8)-C(31)-C(33)	109.3(4)
O(8)-C(31)-C(34)	101.5(3)
C(33)-C(31)-C(34)	112.1(5)
O(8)-C(31)-C(32)	109.9(4)
C(33)-C(31)-C(32)	113.1(5)
C(34)-C(31)-C(32)	110.3(4)
C(31)-C(32)-H(32A)	109.5
C(31)-C(32)-H(32B)	109.5
H(32A)-C(32)-H(32B)	109.5
C(31)-C(32)-H(32C)	109.5
H(32A)-C(32)-H(32C)	109.5
H(32B)-C(32)-H(32C)	109.5
C(31)-C(33)-H(33A)	109.5
C(31)-C(33)-H(33B)	109.5
H(33A)-C(33)-H(33B)	109.5
C(31)-C(33)-H(33C)	109.5
H(33A)-C(33)-H(33C)	109.5
H(33B)-C(33)-H(33C)	109.5
C(31)-C(34)-H(1A)	109.5

C(31)-C(34)-H(1B)	109.5
H(1A)-C(34)-H(1B)	109.5
C(31)-C(34)-H(1C)	109.5
H(1A)-C(34)-H(1C)	109.5
H(1B)-C(34)-H(1C)	109.5

Symmetry transformations used to generate equivalent atoms:

Table S18. Anisotropic displacement parameters ($Å^2$x 10^3) for 007-14186. The anisotropic displacement factor exponent takes the form: $-2\pi^2[h^2 a^{*2}U^{11} + ... + 2 h k a^* b^* U^{12}]$

	U^{11}	U^{22}	U^{33}	U^{23}	U^{13}	U^{12}
N(1)	39(2)	36(2)	42(2)	2(2)	18(2)	4(2)
O(2)	54(2)	48(2)	38(2)	-5(1)	21(1)	3(1)
O(3)	50(2)	42(2)	42(2)	6(1)	24(1)	-1(1)
N(4)	55(2)	34(2)	52(2)	-1(2)	34(2)	2(2)
C(5)	36(2)	35(2)	35(2)	0(2)	18(2)	6(2)
O(6)	51(2)	45(2)	45(2)	1(1)	30(1)	3(1)
N(7)	39(2)	36(2)	36(2)	-3(2)	19(2)	-1(2)
O(8)	35(2)	80(2)	62(2)	-23(2)	26(1)	-6(2)
O(9)	36(2)	102(3)	39(2)	12(2)	11(1)	-5(2)
C(10)	40(2)	38(2)	37(2)	4(2)	19(2)	5(2)
C(11)	40(2)	32(2)	33(2)	0(2)	19(2)	5(2)
C(12)	37(2)	37(2)	41(2)	3(2)	22(2)	4(2)
C(13)	36(2)	40(2)	33(2)	4(2)	16(2)	7(2)
N(14)	35(2)	39(2)	41(2)	-5(2)	16(2)	-3(2)
C(15)	38(2)	45(3)	44(3)	8(2)	23(2)	3(2)
O(16)	46(2)	90(2)	58(2)	-22(2)	17(2)	-13(2)
C(17)	30(2)	36(2)	36(2)	-7(2)	13(2)	-2(2)
C(18)	41(2)	37(2)	42(2)	-3(2)	21(2)	-2(2)
C(19)	40(2)	40(3)	37(2)	-4(2)	21(2)	0(2)
C(20)	27(2)	45(3)	35(2)	-1(2)	14(2)	0(2)
C(21)	38(3)	71(3)	34(2)	3(2)	14(2)	7(2)
C(22)	46(3)	42(3)	41(2)	-10(2)	22(2)	-5(2)
C(23)	35(2)	30(2)	38(2)	1(2)	17(2)	-3(2)
C(24)	35(2)	59(3)	43(3)	-2(2)	18(2)	-10(2)
C(25)	40(3)	65(3)	52(3)	-1(2)	19(2)	-4(2)
C(26)	45(2)	29(2)	43(2)	1(2)	28(2)	-1(2)
C(27)	35(2)	56(3)	51(3)	-8(2)	18(2)	-7(2)
C(28)	57(3)	129(5)	48(3)	32(3)	20(2)	-2(3)
C(29)	59(3)	63(3)	51(3)	14(2)	11(2)	5(2)
C(30)	62(3)	39(3)	70(3)	-6(2)	33(3)	-3(2)
C(31)	35(3)	101(4)	87(4)	-23(3)	29(3)	-4(3)
C(32)	70(4)	128(5)	109(4)	-47(4)	61(3)	-40(3)

C(33)	59(4)	145(6)	121(5)	-7(4)	30(4)	36(4)
C(34)	63(3)	136(5)	104(5)	-57(4)	55(3)	-16(3)

Table S19. Hydrogen coordinates (Å x 10^4) and isotropic displacement parameters (Å^{2}x 10 ^{3}) for 007-14186.

	x	y	z	U(eq)
H(7)	5600(20)	7950(30)	3860(30)	48(12)
H(12)	4227	8505	-247	43
H(15)	2833	8136	-1648	48
H(18A)	5573	8981	330	46
H(18B)	6145	9764	1301	46
H(22A)	6739	9749	3058	50
H(22B)	6758	8740	3811	50
H(24)	1953	5800	380	54
H(25)	1094	5997	-1246	62
H(26)	5291	9620	2662	43
H(28A)	1028	7911	-3809	117
H(28B)	2033	7680	-3222	117
H(28C)	1566	8731	-2921	117
H(29A)	6764	4764	3220	90
H(29B)	6409	5673	3789	90
H(29C)	7129	6053	3385	90
H(30A)	6403	4143	1613	83
H(30B)	6362	4732	623	83
H(30C)	5499	4567	831	83
H(32A)	8663	9356	1512	141
H(32B)	9597	8788	1799	141
H(32C)	9245	9118	2628	141
H(33A)	9320	7157	3293	164
H(33B)	9777	6709	2588	164
H(33C)	8924	6088	2586	164
H(1A)	8239	6362	833	141
H(1B)	8998	7113	725	141
H(1C)	8046	7635	390	141

Table S20. Hydrogen bonds for 007-14186 [Å and °].

D-H...A	d(D-H)	d(H...A)	d(D...A)	<(DHA)
N(7)-H(7)...O(2)#1	0.97(4)	1.91(4)	2.869(4)	169(3)

Symmetry transformations used to generate equivalent atoms:

#1 x,-y+3/2,z+1/2

Bibliography.

[1] S. Zhu, **2010**, US 20100087469 A1

[2] Z. E. D. Torres, E. R. Silveira, L. F. R. E. Silva, E. S. Lima, M. C. de Vasconcellos, D. E. D. Uchoa, R. Braz Filho, A. M. Pohlit, *Molecules* **2013**, *18*, 6281-6297.

[3] The modified functional was invoked using WC04 internal option (IOp) statements and the B3LYP functional.

[4] The modified functional was invoked using WC04 internal option (IOp) statements and the B3LYP functional.

[5] The modified functional was invoked using WC04 internal option (IOp) statements and the B3LYP functional.

[6] The modified functional was invoked using WC04 internal option (IOp) statements and the B3LYP functional.

[7] J. D. Johnson, R. A. Dennull, L. Gerena, M. Lopez-Sanchez, N. E. Roncal, N. C. Waters, *Antimicrob. Agents Chemother.* **2007**, *51*, 1926-1933.

[8] P. H. Willoughby, M. J. Jansma, T. R. Hoye, *Nat. Protocols* **2014**, *9*, 643-660.

[9] W. L. Jorgensen, J. Tirado–Rives, *J. Comput. Chem.* **2005**, *26*, 1689-1700.

[10] M. J. T. Frisch, G. W.; Schlegel, H. B.; Scuseria, G. E.; Robb, M. A.; Cheeseman, J. R.; Scalmani, G.; Barone, V.; Mennucci, B.; Petersson, G. A.; Nakatsuji, H.; Caricato, M.; Li, X.; Hratchian, H. P.; Izmaylov, A. F.; Bloino, J.; Zheng, G.; Sonnenberg, J. L.; Hada, M.; Ehara, M.; Toyota, K.; Fukuda, R.; Hasegawa, J.; Ishida, M.; Nakajima, T.; Honda, Y.; Kitao, O.; Nakai, H.; Vreven, T.; Montgomery, J. A., Jr.; Peralta, J. E.; Ogliaro, F.; Bearpark, M.; Heyd, J. J.;

Brothers, E.; Kudin, K. N.; Staroverov, V. N.; Kobayashi, R.; Normand, J.; Raghavachari, K.; Rendell, A.; Burant, J. C.; Iyengar, S. S.; Tomasi, J.; Cossi, M.; Rega, N.; Millam, J. M.; Klene, M.; Knox, J. E.; Cross, J. B.; Bakken, V.; Adamo, C.; Jaramillo, J.; Gomperts, R.; Stratmann, R. E.; Yazyev, O.; Austin, A. J.; Cammi, R.; Pomelli, C.; Ochterski, J. W.; Martin, R. L.; Morokuma, K.; Zakrzewski, V. G.; Voth, G. A.; Salvador, P.; Dannenberg, J. J.; Dapprich, S.; Daniels, A. D.; Farkas, Ö.; Foresman, J. B.; Ortiz, J. V.; Cioslowski, J.; Fox, D. J., Gaussian Inc., Wallingford CT, **2009**.

[11] W. C. Still, M. Kahn, A. Mitra, *J. Org. Chem.* **1978**, *43*, 2923–2925.

[12] A. B. Pangborn, M. A. Giardello, R. H. Grubbs, R. K. Rosen, F. J. Timmers, *Organometallics* **1996**, *15*, 1518–1520.

[13] H. J. Dauben, L. L. Mccoy, *J. Am. Chem. Soc.* **1959**, *81*, 4863–4873.

[14] R. M. R. Sarmiento, M. H. Nettekoven, S. Taylor, J.-M. Plancher, H. Richter, O. Roche, *Bioorg. Med. Chem. Lett.* **2009**, *19*, 4495–4500.

[15] J. C. S. Woo, E. Fenster, G. R. Dake, *J. Org. Chem.* **2004**, *69*, 8984–8986.

[16] W. Trager, J. B. Jensen, *Science* **1976**, *193*, 673-675.
